# Designer peptide–DNA cytoskeletons regulate the function of synthetic cells

**DOI:** 10.1038/s41557-024-01509-w

**Published:** 2024-04-23

**Authors:** Margaret L. Daly, Kengo Nishi, Stephen J. Klawa, Kameryn Y. Hinton, Yuan Gao, Ronit Freeman

**Affiliations:** Department of Applied Physical Sciences, University of North Carolina, Chapel Hill, NC USA

**Keywords:** Self-assembly, Organizing materials with DNA

## Abstract

The bottom-up engineering of artificial cells requires a reconfigurable cytoskeleton that can organize at distinct locations and dynamically modulate its structural and mechanical properties. Here, inspired by the vast array of actin-binding proteins and their ability to reversibly crosslink or bundle filaments, we have designed a library of peptide–DNA crosslinkers varying in length, valency and geometry. Peptide filaments conjoint through DNA hybridization give rise to tactoid-shaped bundles with tunable aspect ratios and mechanics. When confined in cell-sized water-in-oil droplets, the DNA crosslinker design guides the localization of cytoskeletal structures at the cortex or within the lumen of the synthetic cells. The tunable spatial arrangement regulates the passive diffusion of payloads within the droplets and complementary DNA handles allow for the reversible recruitment and release of payloads on and off the cytoskeleton. Heat-induced reconfiguration of peptide–DNA architectures triggers shape deformations of droplets, regulated by DNA melting temperatures. Altogether, the modular design of peptide–DNA architectures is a powerful strategy towards the bottom-up assembly of synthetic cells.

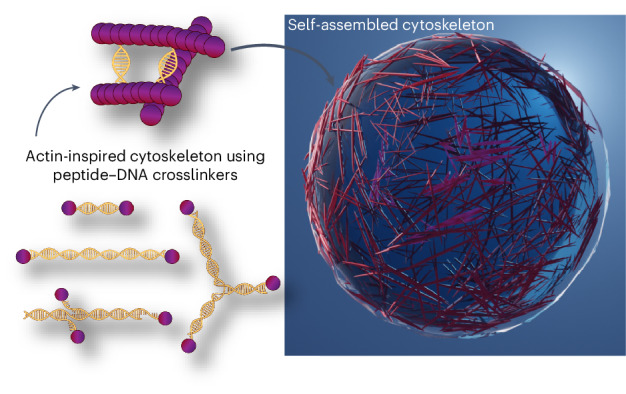

## Abstract (web summary)

Advances in the development of cytoskeletal-like materials with modular structures and mechanics are pivotal for the engineering of synthetic cells. Now actin-mimetic supramolecular peptide networks have been designed using programmable peptide–DNA crosslinkers, giving rise to tunable tactoid-shaped bundles and mechanical properties that control spatial localization, the diffusion of payloads and shape changes within artificial cells.

## Main

Engineering synthetic cytoskeletons is essential for the bottom-up construction of artificial cells. The cellular cytoskeleton consists of hierarchical and dynamic polymers that function as scaffolding components of cells and drive vital processes, including cell division, motility and morphogenesis^1^. These functionalities are governed by the spatial organization of the cytoskeletal components inside cells and in contact with membranes. The cytoskeleton’s ability to transition between various architectures, ranging from filament networks to aligned bundles or spindles, is regulated by numerous associating proteins^2,3^. These proteins regulate the nucleation, elongation, branching, severing, capping, bundling and crosslinking of filaments to shape cells^1,4,5^.

In vitro systems using purified proteins have expanded our understanding of how cytoskeletal filaments and their associating proteins shape cells. Purified proteins reconstituted within or on cell-sized vesicles and droplets have been used to explore the effect of actin organization on cell shape^6^. For example, the crosslinking of actin with fascin, actinin or filamin in giant unilamellar vesicles was investigated to understand how different bundled actin assemblies induce membrane deformation^7–10^.

Recently, engineering of the synthetic cytoskeleton has been proposed as a pathway to generate artificial cells^11^. While various building blocks form filaments and networks in the bulk, including polymers^12^, small molecules^13^, carbon nanotubes^14^, peptides^13,15^ and DNA^16–18^, assembly in cell-like confinement has so far mainly relied on DNA material^19–23^. The bundling of DNA nanofilaments in protocells has been achieved by adding crowding agents^20^ or salt^23^, which is challenging to reverse or fine-tune. Another synthetic challenge is to recruit structures to the periphery or lumen of droplets. While cholesterol^20,23^ or lipid tail^24^ modifications have been used to localize structures to membranes, a more controlled spatial localization would enable cytoskeletal–membrane mechanical and biochemical crosstalk towards constructing deformable and responsive artificial cells.

Peptides are a less used, yet promising building block for the construction of synthetic cytoskeletons. The rational design of peptides assembling into diverse structures across length scales has been extensively studied in the bulk^25,26^. Yet, only a handful of peptide-based systems have been realized in cell-like confinement^24,27–31^. Peptide filament assembly in water-in-oil droplets has been triggered by pH or salt^24,28,29^, and short self-assembled peptides have been shown to stabilize water-in-oil droplets^30,32,33^. Unleashing the full potential of peptide-based self-assembled systems in confinement will introduce unique properties and capabilities into synthetic cells.

**Fig. 1:**
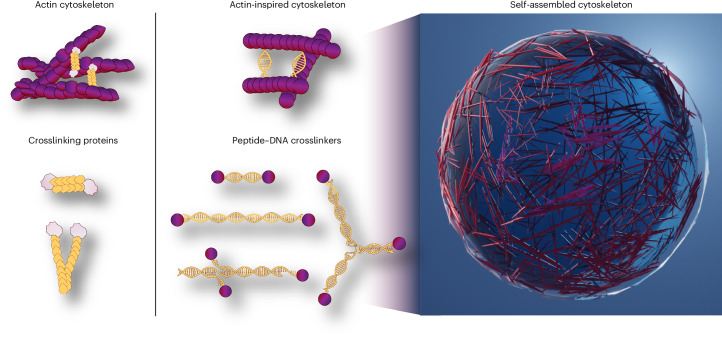
Peptide–DNA nanotechnology for the construction of synthetic cytoskeletons. Peptide–DNA filaments crosslinked via programmable complementary DNA interactions (middle), mimicking actin (left), and its associated proteins. The tunable organization of the peptide–DNA cytoskeleton in cell-sized confinement (right) guides the functions of artificial cells.

In this study, we married peptide self-assembly with DNA programmability to realize a synthetic cytoskeleton in droplets (Fig. 1). Inspired by actin-binding proteins, we rationally designed peptide–DNA crosslinkers with varying sequence, length, valency and geometry. We show here how filamentous peptides conjoined through DNA hybridization form tactoid-shaped bundles and networks with tunable aspect ratios and mechanics. When confined within cell-sized water-in-oil droplets, distinct structures are driven to spatially localize in the cortex or lumen, depending on the crosslinker attributes, and the extent of bundling tunes the mobility of intradroplet payloads from water-like to arrested. Finally, we show how different crosslinkers orchestrate changes in the cellular shape of lipid-encased droplets. Our programmable peptide–DNA nanotechnology approach is a powerful platform towards the construction of functional, fully artificial cells.

## Results and discussion

### Peptide–DNA constructs crosslinking cytoskeletal filaments

Cytoskeletal actin filaments associate with actin-binding crosslinkers of different geometries and flexibilities to form mesoscale functional structures, such as stress fibres, filopodia and the cell cortex^1^. For instance, fascin organizes actin filaments into bundles to regulate cell migration, while filamin crosslinks actin to form networks in the cell cortex^3,34^.

Given the essential role of actin-binding proteins in regulating various cytoskeletal arrangements, we hypothesized that mimetic crosslinkers will extend the functionality of synthetic filamentous systems to yield reconfigurable cytoskeletal superstructures. To test this, we used the emerging class of peptide–DNA materials, uniquely integrating peptide self-assembly with DNA programmability^35,36^. Previously, Stupp and co-workers showed that lipid–peptides decorated with complementary oligonucleotides guide the reversible formation of microscale bundles^37^. As a platform to generalize peptide–DNA nanotechnology, our group subsequently showed that mixing short amyloid peptides bearing complementary oligonucleotides drives a reversible fibre-to-bundle transition^38^. Owing to the wealth of possible DNA secondary structures with adjustable valencies and geometries beyond the simplest double helix (‘duplex’), DNA offers a rich, programmable parameter space that is not so readily achievable with other polymeric, small molecule or ionic crosslinking systems^39^. Using DNA to bundle peptide filaments, akin to actin and its binding proteins, provides a general methodology for fine-tuning peptide hierarchical organization and is a powerful approach towards building an artificial cytoskeleton.

We generated a library of peptide–DNA monomers^38^ consisting of fibre-forming dipeptides (fluoren-9-ylmethoxycarbonyl-Phe-Phe-OH (Fmoc-FF-OH))^40,41^ with complementary DNA sequences of various lengths and junction geometries (Supplementary Figs. 1–4, Supplementary Video 1 and Supplementary Table 1). Duplexes of different lengths (A′ with 8 base pairs (bps) and A-A′ with 14 bps) mimic linear actin crosslinkers such as fascin and actinin, respectively, while changing the junction geometry from linear to branched mimics crosslinkers such as filamin or heavy meromyosin. The copolymerization of peptides with hybridizing peptide–DNAs yields weakly to strongly crosslinked peptide constructs (Fig. 1, Supplementary Figs. 5 and 6 and Supplementary Table 2).

**Fig. 2:**
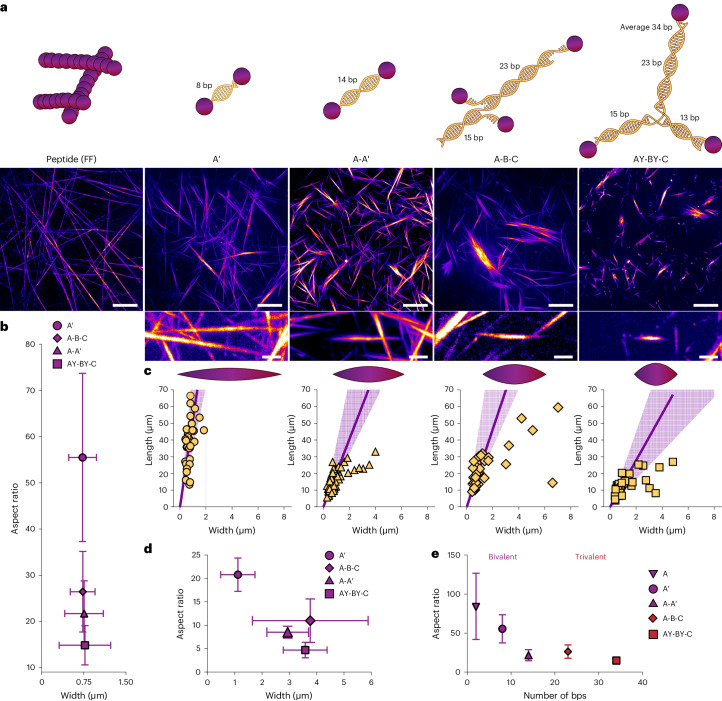
Tunable mimetic cytoskeleton arrangement using peptide–DNA. **a**, Hierarchical filament organization using various DNA crosslinkers. Top: schematics of DNA-crosslinked peptide fibres and the DNA crosslinkers. The number of base pairs in the peptide–DNA duplexes is noted (for AY-BY-C, the average number of base pairs between peptides (34) is also noted). Middle: wide-field confocal microscopy images of the corresponding assemblies (1 mol% of each crosslinker). Scale bars, 20 μm. Bottom: magnified views of the spindle-like structures, referred to as thin bundles (no image is shown of the peptide alone because no spindles were formed). Scale bars, 3 μm. **b**, Aspect ratios of the thin bundles (width < 1 μm) depicted in **a** (bottom), plotted against width. The data are presented as the mean ± standard deviation (s.d.). **c**, Lengths of all of the bundles versus their widths. The purple lines show the linear fits and the shaded regions show the range of thin-bundle measurements. The images above the plots are representative bundle shapes. **d**, Aspect ratio versus width for large bundles. The data are presented as the mean ± s.d. **e**, Aspect ratios of thin bundles versus the number of bps between peptides in each DNA crosslinker (A, 2 bp; A′, 8 bp; A-A′, 14 bp; A-B-C, 23 bp; AY-BY-C, average of 34 bp). The data are presented as the mean ± s.d. A minimum of *n* = 34 bundles were analysed for each crosslinker in **b**–**e**. Source data

To evaluate the emergent effect of DNA crosslinkers on the structural organization of peptides, we prepared solutions of 0.1 wt% peptide (1.87 mM) containing 1 mol% of the DNA crosslinker. The various peptide and peptide–DNA mixtures were annealed (in 1% dimethylsulfoxide (DMSO)–water) to optimize DNA hybridization (Methods) and imaged via confocal microscopy (Fig. 2, Supplementary Discussion 1 and Supplementary Fig. 7). Without DNA crosslinkers, Fmoc-FF-OH peptides (‘FF’) form microscale-long thin filaments, as expected^38,40^. As DNA crosslinkers with linear (A′ and A-A′) or branched (A-B-C and AY-BY-C) junctions were introduced, hierarchical bundles with an increasing degree of alignment evolved (Fig. 2a, top and middle). Quantification of the length, width and aspect ratio (length/width) of the bundles revealed two distinct hierarchical states: thin bundles and large bundles (or bundles of bundles; Fig. 2b–d and Supplementary Fig. 8). The thin bundles adopt spindle-like tactoid structures^42^ (Fig. 2a, bottom, and Supplementary Fig. 9) with remarkable similarity to reconstituted actin and microtubule bundles^43,44^. As the linear crosslinker length increases from 8 bps (A′) to 14 bps (A-A′), the aspect ratio decreases. The spindle aspect ratio decreases further upon introducing multi-stranded DNA crosslinkers with increasing numbers of base pairs between the peptides (A-B-C, 23 bps; AY-BY-C, average of 34 bps; Fig. 2b,e). Confocal microscopy intensity line scans across large-bundle widths in A-A′ and A-B-C (Supplementary Fig. 10) show multiple peaks, each with a size similar to that of thin-bundle widths (~1 μm), indicating that the large bundles consist of multiple spindle-like thin bundles. The large-bundle aspect ratio also decreases when transitioning from linear (A′ and A-A′) to multi-stranded DNA crosslinkers (AY-BY-C; Fig. 2d).

**Extended Data Fig. 1:**
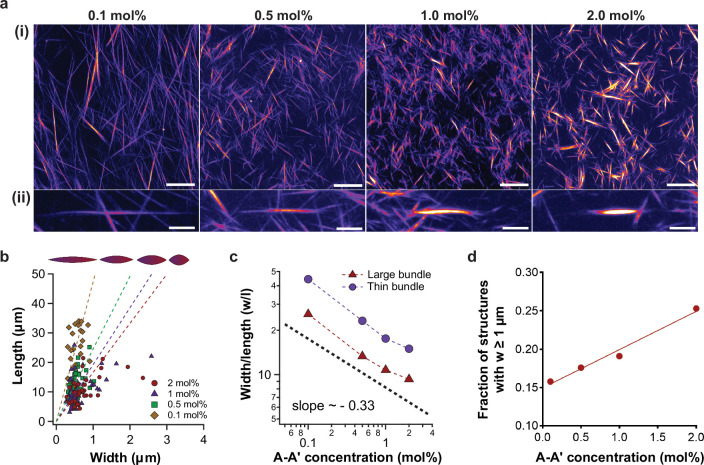
Varying DNA-crosslinker (A-A′) concentration. (**a**) Maximum intensity projection confocal images of A-A′ assemblies containing 0.1, 0.5, 1.0 and 2.0 mol% of DNA-crosslinker (left to right, respectively). Scale bars: (i) 20 μm, (ii) 5 μm. (**b**) Lengths of A-A′ bundles plotted against their widths with various A-A′ concentrations. (Red circle: 2 mol%, Blue triangle: 1 mol%, Green square: 0.5 mol%, Yellow diamond: 0.1 mol%) Dashed lines represent linear fitting results for thin bundles, which correspond to their average aspect ratios. (**c**) Average aspect ratios (diameter/length) of thin spindle-like bundles and large bundles plotted against the mole percent of DNA-crosslinker. A black dotted line is used as a visual representation of the power-law function with a slope of ~ −0.33 for reference. (**d**) Fraction of structures with widths equal to or above 1 μm plotted against the mole percent of DNA-crosslinker and fit with a linear regression (y=0.04980x+0.1497, R^2^=0.9806). The calculation protocol is detailed in Supplementary Fig. 11. Source data

Next, we investigated the effect of crosslinker fraction (0.1, 0.5, 1 and 2 mol%) on bundle formation and their dimensions (Extended Data Fig. 1a and Supplementary Fig. 11). As the DNA concentration increases, both thin and large bundles exhibit power law decreases in aspect ratio, with an exponent of around −0.33 (Extended Data Fig. 1b,c). Crosslinked F-actin tactoids demonstrate a similar weak power law behaviour, indicating that our approach successfully mimics natural cytoskeletons^43,44^. In addition, a higher DNA crosslinker concentration generates more bundles and a larger network mesh size (Extended Data Fig. 1d and Supplementary Figs. 11 and 12). To show that the formation of DNA-crosslinked bundles is not limited to specific DNA sequences, we tested an alternative DNA duplex consisting of poly(A)·poly(T) (A_m_-A′_m_). We adjusted its length to 25 bps (compared with 14 bps in A-A′) to match the melting temperature of A-A′ (that is, its binding strength; Supplementary Table 2 and Supplementary Fig. 13). Confocal microscopy revealed tactoid-shaped bundles with similar aspect ratios to A-A′, but more loosely packed, as expected for a longer duplex (Supplementary Figs. 8, 13 and 14). These results demonstrate that an array of tunable peptide–DNA crosslinkers replicates the structural arrangements of the natural cytoskeleton using simplistic molecular components.

### Mechanistic insights into peptide–DNA tactoid formation

**Extended Data Fig. 2:**
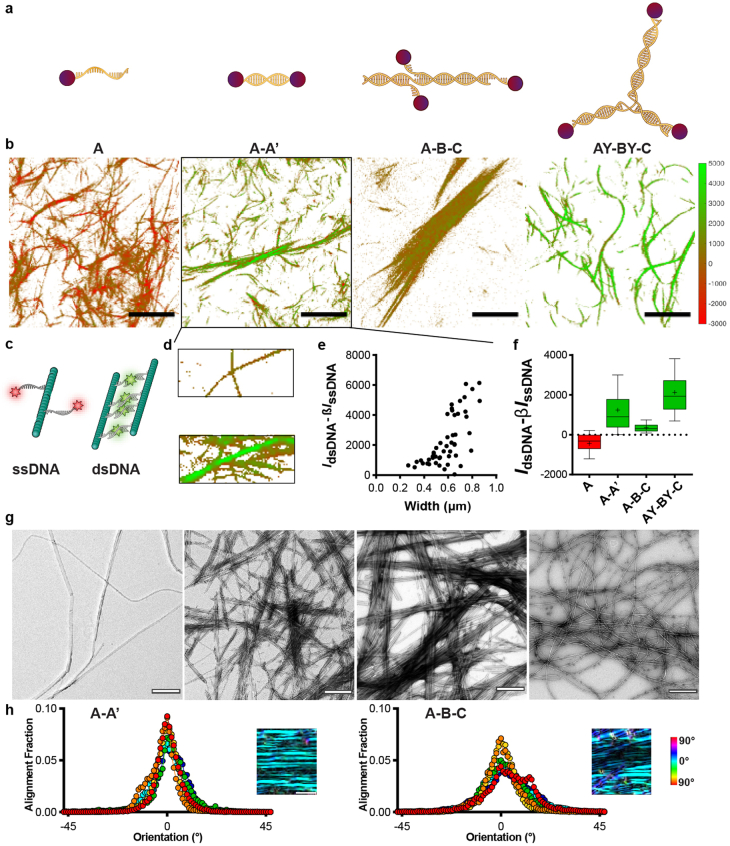
Crosslinker density and filament alignment within bundled networks. (**a**) Schemes of peptide–DNA crosslinkers. (**b–f**) Acridine orange (AO) staining of peptide–DNA materials (1 mole% crosslinker) for the analysis of DNA hybridization (AO fluoresces red in the presence of ssDNA and green in the presence of dsDNA). (**b**) The spatial distributions of *I*_dsDNA_- *βI*_ssDNA_ values, where *I*_dsDNA_ and *I*_ssDNA_ represent the fluorescence intensities of dsDNA and ssDNA, respectively. *β* denotes the ratio of fluorescence intensities of peptides between green and red channels. *I*_dsDNA_- *βI*_ssDNA_ values were estimated from the green and red fluorescence images of the respective structures (Supplementary Figs. 16, 17) as discussed in Supplementary Discussion 2 and Supplementary Methods section. Scale bars: 15 μm. (**c**) Scheme of acridine orange emission from peptide-ssDNA filaments (red) and bundles (green). (**d**) Enlarged images from (b), showing thinner species with ssDNA content and thicker species with high dsDNA content in A-A′. (**e**) Average *I*_dsDNA_- *βI*_ssDNA_ values within a given bundle in A-A′ plotted against the width of the bundle. (**f**) Box-and-whisker plots of *I*_dsDNA_- *βI*_ssDNA_ values derived from the histogram of pixel values generated in the color maps (*n* = total pixels from 3 images; 336597 for A, 122395 fo C'-C, 337660 for B-C, 273243 for A-A', 260904 for A-C, 212742 for A-B-C, 15524 for AY-BY-C). Center line is median, cross is the mean, the box extends to the 25th and 75th percentiles, and whiskers extend to the 10th and 90th percentiles. Statistics were performed with a Kruskal-Wallis test (all comparisons: **** p < 1 × 10^−15^). (**g**) TEM images of peptide–DNA assemblies (A, A-A′, A-B-C, AY-BY-C). Scale bars: 1 μm. (**h**) Histograms of the relative alignment angles of fibres within bundles from TEM images (*n* = 6 bundles) for A-A′ (left) and A-B-C (right). Insets of representative TEM images for A-A′ and A-B-C are falsely colored with the angle of alignment. Source data

We wished to explore why tactoids were formed in DMSO–water, while previously reported DNA-bundled peptides in pure water did not exhibit tactoid shapes^37,38^. Filament length was previously shown to be an important parameter for the assembly of actin into bundles (longer filaments) or tactoids (shorter filaments)^43^. As supramolecular peptide polymerization is highly sensitive to solvent^45^, we hypothesized that assembly in aqueous buffers instead of DMSO–water would enhance the hydrophobic collapse of FF peptides, resulting in longer filaments^46^. Indeed, confocal imaging of FF assemblies in water revealed longer filaments than in DMSO–water (median length: 63 μm in water versus 35 μm in DMSO–water; Supplementary Fig. 15), and the co-assembly of FF with single-stranded peptide–DNA (A) also yielded longer filaments in water (26 μm in DMSO–water versus 38 μm in water; Supplementary Fig. 15). Introducing DNA crosslinkers into water yielded long bundles of aligned filaments that did not exhibit tactoid shapes (Extended Data Fig. 2 and Supplementary Figs. 16 and 17). Larger peptide–DNA bundles correlated with higher DNA densities (Extended Data Fig. 2a–f) and exhibited high alignment with short and stiff crosslinkers (Extended Data Fig. 2g,h and Supplementary Fig. 18). These results indicate that, similar to crosslinked actin, longer peptide filaments give rise to long bundles, while shorter filaments produce tactoids (Supplementary Discussion 2 and Supplementary Fig. 19). We therefore wondered whether sonicating peptide–DNAs assembled in water would shorten filaments to produce tactoids. Accordingly, we assembled A-A′ in water to yield long bundles, which we then sonicated for 20 min (Supplementary Fig. 20). Confocal imaging revealed tactoid-shaped bundles with a decreased average length of 5.4 μm (initial length of 42 μm). Altogether, the comparison of assemblies in water and DMSO–water demonstrates that filament length is key to DNA-crosslinked tactoid formation, as has been shown previously for actin^43^.

Finally, we explored the role of DNA hybridization in bundle formation by monitoring the annealing of A-A′ (melting temperature, *T*_m_ = 64 °C) and FF by confocal imaging and circular dichroism (CD) spectroscopy. Confocal imaging revealed that heating A-A′ at 95 °C for 25 min followed by cooling to 50 °C (below *T*_m_) resulted in the dissociation of the initial aggregates, followed by the formation of tactoids (Supplementary Fig. 21a). An annealing time of at least 10 min was needed at 95 °C to fully dissolve the aggregates of A-A′ and promote tactoids. Conversely, the annealing of FF produced long filaments independent of the annealing time, suggesting that DNA crosslinker is essential for tactoid formation. CD analysis confirmed DNA hybridization upon cooling A-A′, as evidenced by the characteristic duplex peak at 280 nm (Supplementary Fig. 21b–d). Analysis of the CD spectra of FF, A-A′ and AY-BY-C at 280 nm confirmed the formation of DNA crosslinks, with the temperature profiles matching their respective melting temperatures.

Taken together, these results demonstrate that DNA crosslinker length and valency tune bundle aspect ratio and alignment, while filament length guides the formation of bundles (longer filaments) or tactoids (shorter filaments).

### Reversible mechanical properties of bundled architectures

**Fig. 3:**
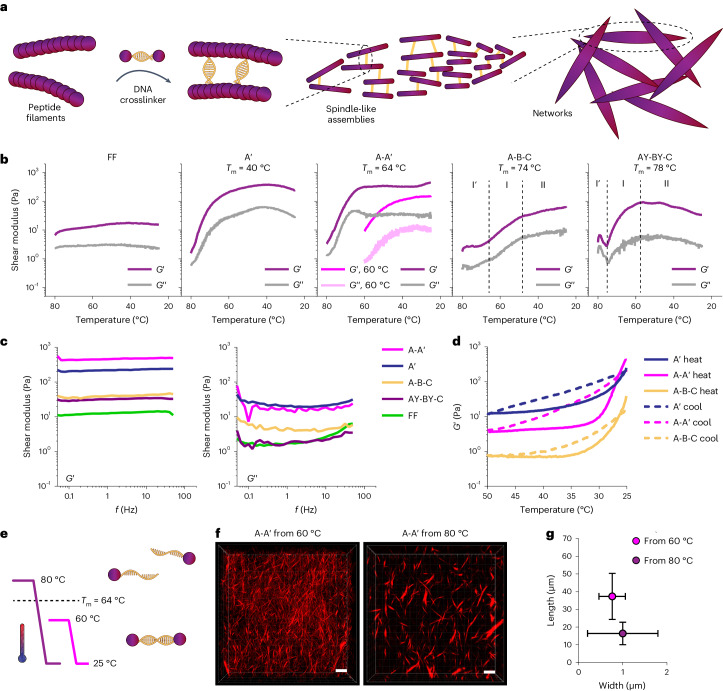
Mechanics of peptide–DNA cytoskeletal networks. **a**, Illustration of the hierarchical assembly of peptide–DNA filaments into a spindle-like network. **b**, Storage (*G*′) and loss (*G*″) moduli of the peptide–DNA networks during the annealing process versus temperature for a cooling rate of −1 °C min^−1^ from 80 to 25 °C when 1 mol% peptide–DNA crosslinker was introduced into peptide networks. For A-B-C and AY-BY-C, we observe three distinct phases in the annealing process: region I′ is a lag phase, followed by a monotonic increase in the shear modulus from around 65 °C for A-B-C and 70 °C for AY-BY-C in region I, reaching a plateau at relatively low temperatures in region II. For A-A′, annealing was also performed at a cooling rate of −1 °C min^−1^ from 60 to 25 °C. **c**, Storage (*G*′) and loss (*G*″) moduli of the peptide–DNA cytoskeletal materials versus frequency. **d**, Reversibility of the storage modulus (*G*′) of A′, A-A′ and A-B-C cycled from 25 °C to 50 °C to 25 °C. **e**, Schematic of slow cooling from above (80 °C) or below (60 °C) the DNA melting temperature of A-A′. **f**,**g**, Confocal microscopy images (**f**) and aspect ratio plotted as length versus width (**g**) for A-A′ annealed from 60 and 80 °C at a cooling rate of −1 °C min^−1^. Scale bars in **f**, 30 μm. In **g**, *n* = 53 and 40 bundles were analysed after annealing from 80 and 60 °C, respectively. Source data

The mechanical properties of cytoskeletal actin bundles vary by orders of magnitude depending on the bundle length, diameter, crosslinking protein type and concentration^47^. To examine our synthetic cytoskeleton mechanics, we first performed frequency sweep tests on bulk networks prepared in DMSO–water (Fig. 3a,c and Supplementary Figs. 22 and 23). The linearly crosslinked gels A′ and A-A′ exhibited a higher complex shear modulus (*G*_ω_) than the bundled networks formed by the trivalent crosslinkers A-B-C and AY-BY-C under the same peptide and crosslinker concentrations, as well as a more than tenfold higher *G*_ω_ compared with peptide-only gels (FF; Fig. 3c). These observations can be attributed to the competition between the crosslinks in the bundles and networks, as previously observed for native cytoskeleton^48,49^. The linear peptide–DNA crosslinkers generate thin bundles, yielding denser networks (smaller mesh size, *ξ*), while the trivalent crosslinkers produce larger bundles, generating looser networks (larger mesh size; Supplementary Figs. 24 and 25).

As DNA crosslinking is sensitive to its melting temperature, we monitored *G*_ω_ at distinct annealing temperatures. While FF showed no temperature dependence, A′ and A-A′ showed monotonic increases in *G*_ω_ during cooling, suggesting effective crosslinking. Furthermore, A-A′ exhibited a lower *G*_ω_ (threefold) when cooled from 60 °C (<*T*_m_) to 25 °C at a rate of −1 °C min^−1^ than when cooled from 80 °C (>*T*_m_; Fig. 3b). Imaging revealed that cooling from 80 °C yielded bundled networks with smaller (twofold) aspect-ratio bundles (Fig. 3b,e–g), indicating enhanced crosslinking. These results suggest that the hybridization temperature of the peptide–DNA crosslinkers is an effective tool for programming different structural and mechanical states from the same building blocks. Changing the crosslinker from bivalent to trivalent (A-B-C and AY-BY-C) and monitoring *G*_ω_ during annealing (80–25 °C) revealed three distinct rheological regimes (Fig. 3b). In the first phase of cooling (region I′), *G*_ω_ was stable. As the temperature was decreased to 75 °C (AY-BY-C) or 65 °C (A-B-C), *G*_ω_ increased (region I) until reaching a plateau (region II). We postulate that this rheological profile reflects a multistep evolution of large bundles (region I′, steady *G*_ω_) and a crosslinked network (region I) during cooling. As simulated in Supplementary Fig. 6, AY-BY-C has three duplex regions and a higher *T*_m_, while A-B-C has two duplex regions and a lower *T*_m_, indicating that the partial binding of trivalent crosslinkers generates large bundles, slowing the increase in *G*_ω_.

As DNA hybridization is thermally reversible, we tested how heating affects the mechanics of pre-assembled DNA-crosslinked networks. Heating A′ to 40 °C (Supplementary Fig. 26) or 50 °C at a rate of +1 °C min^−1^, followed by cooling to 25 °C at −1 °C min^−1^ (Fig. 3d and Supplementary Fig. 27) revealed a significant decrease in *G*_ω_ as the temperature increased, with excellent reproducibility upon cooling, while FF exhibited no temperature dependence (Supplementary Fig. 26). This indicates that the thermal dependence of A′ is due to the formation (during cooling) and dissociation (during heating) of DNA crosslinks. Live imaging of assembled A′ bundles at 60 °C (above *T*_m_; Supplementary Video 2) revealed Brownian-type fluctuations that were arrested after cooling. This suggests that heating destabilizes the crosslinks between thin bundles, reducing *G*_ω_. The heating and subsequent cooling of the A-A′ and A-B-C crosslinked networks (Fig. 3d and Supplementary Fig. 28a) showed similar trends to A′, with *G*′ dropping over tenfold within an increase of only 10 °C from room temperature, well below the melting temperature, suggesting that weak crosslinks between thin bundles are destabilized first, having a significant effect on *G*_ω_. We compared the relative fractions of weak crosslinks (the difference between *G*′ at 25 and 50 °C) in each system (Supplementary Fig. 28b) and found that A-B-C exhibited the smallest change in *G*′, suggesting that A-B-C has more stable crosslinks within large bundles than within networks. Conversely, A-A′ showed the largest difference in *G*′, suggesting that A-A′ crosslinks filaments to form thin bundles and connects bundles into networks. These heating/cooling cycles demonstrate that peptide–DNA-crosslinked networks exhibit thermoreversible properties that allow recursive transitions between different structural and mechanical states.

Overall, we have demonstrated the interplay between the synthetic cytoskeleton architecture and its mechanical properties. Our peptide–DNA design offers materials with tunable mechanical elasticities stored in the structural design, with the ability to reconfigure networks due to the reversibility of DNA crosslinking, akin to native cytoskeletons.

### Spatial localization of cytoskeletal mimetic in cell-sized droplets

**Fig. 4:**
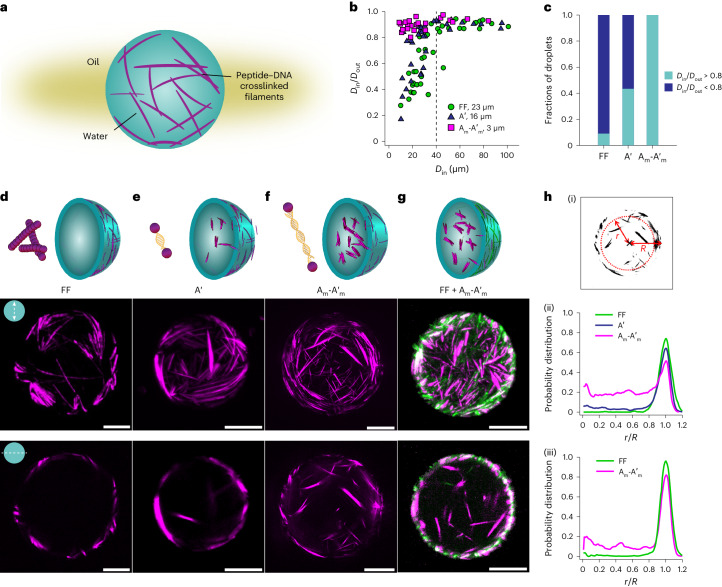
Spatial distribution of peptide–DNA networks in cell-sized confinement. **a**, Schematic of the synthetic cytoskeleton in water-in-oil droplets. **b**, Sphericity (*D*_in_/*D*_out_) versus size distribution (diameter *D*_in_) of droplets containing FF, A′ or A_m_-A′_m_ (0.5 mol% peptide–DNA crosslinker). Droplets with a diameter >40 μm are highly spherical for all cytoskeletal reinforced droplets, while for a diameter <40 μm, droplets deviate from sphericity at different size thresholds (as indicated in the legend and derived by fitting the curves), depending on the cytoskeletal arrangement. **c**, Fractions of spherical (*D*_in_/*D*_out_ > 0.8) and non-spherical (*D*_in_/*D*_out_ < 0.8) droplets containing FF, A′ or A_m_-A′_m_. **d**–**g**, Spatial distribution of cytoskeletal structures inside droplets. Schematics (top) and confocal images (middle and bottom) of spherical droplets (≳40 μm) containing FF (**d**), A′ (**e**) and A_m_-A′_m_ (**f**) stained with Nile red and of droplets of FF + A_m_-A′_m_ stained with both ThT (FF, green channel) and Nile red (A_m_-A′_m_, magenta channel) (**g**). The confocal images show maximum intensity projections (middle) and equatorial *z* slices of the droplets. Scale bars, 20 μm. **h**, Schematic defining the ratio *r*/*R* and explaining the calculation process of a probability distribution using a binarized image of an equatorial *z* slice of a droplet (i) and probability distributions of structures versus droplet diameter ratio *r*/*R* for FF, A′ and A_m_-A′_m_ stained with Nile red (ii) and a mixture of FF (stained with ThT) and A_m_-A′_m_ (stained with Nile red) (iii). *n* = 7 for FF, *n* = 18 for A′, *n* = 9 for A_m_-A′_m_ and *n* = 11 for FF + A_m_-A′_m_. Source data

To demonstrate the utility of peptide–DNA synthetic cytoskeletons, we encapsulated various assemblies within cell-sized confinements to produce a set of synthetic cells with programmed ‘cytoskeletal states’. Peptide (FF) or DNA-crosslinked peptide structures pre-assembled in DMSO–water (A′ and A_m_-A′_m_ at 0.5 mol% crosslinker) were mixed with oil to form membraneless water-in-oil droplets (Fig. 4a). Confocal microscopy revealed the successful encapsulation of the structures in the droplets (diameter < 100 μm; Fig. 4d–f and Supplementary Figs. 29 and 30). The encapsulated crosslinked structures exhibited similar dimension trends to those in the bulk (Supplementary Fig 31). To quantify droplet shape, we calculated the diameters of the maximum inscribed circle (*D*_in_) and circumscribed circle (*D*_out_) of maximum *z* projections and plotted their ratio (*D*_in_/*D*_out_) as a function of droplet size (*D*_in_; Fig. 4b and Supplementary Fig. 32). The *D*_in_/*D*_out_ ratio approaches 1 for spherical droplets and is less than 1 for deformed droplets. For FF and A′, droplets larger than ~40 μm remained spherical (*D*_in_/*D*_out_ ≈ 0.9), while smaller droplets (≲40 μm) tended to deform. In contrast, A_m_-A′_m_ droplets consistently formed spherical shapes (*D*_in_/*D*_out_ ≈ 0.9) across the entire size range. Figure 4c illustrates the fraction of droplets (≲40 μm) exhibiting spherical (*D*_in_/*D*_out_ > 0.8) and non-spherical (*D*_in_/*D*_out_ < 0.8) shapes, demonstrating that droplets with assemblies crosslinked with longer crosslinkers (FF, 0 bp; A′, 8 bps; A_m_-A′_m_, 25 bps) promote spherical shapes. This behaviour can be attributed to the shorter axial length of structures with DNA crosslinkers (Fig. 2 and Supplementary Fig. 16). Longer fibres, such as FF and A′, require a higher bending energy to be encapsulated in confined spaces^50^, leading to the deformation of smaller droplets with higher curvature. Indeed, droplets with shorter sonicated FF filaments promote the formation of spherical droplets (Supplementary Fig. 33).

Next, we investigated the effects of DNA crosslinkers on the spatial localization of structures within spherical droplets (≳40 μm). Confocal spectroscopy revealed FF fibres primarily localized at oil–water interfaces, resembling the natural actin cortex layer, while DNA-crosslinked bundles were found more in the interior of droplets (Fig. 4d–h, Supplementary Fig. 30 and Supplementary Videos 3–5). To evaluate the spatial distributions of structures, we plotted the probability distribution (defined as the circular average of binarized images of one *z* slice) as a function of distance from the centre (*r*) of the droplet, normalized by the radius of the droplet (*R*; Fig. 4h(ii)). For FF, the distribution peaks are observed at the periphery of droplets (*r*/*R* = 1), indicating that peptide fibres localize at the oil–water interface due to the amphiphilic properties of FF, as shown previously for reversed organic solvent droplets in water^30^. Shorter (sonicated) FF filaments are more readily incorporated into droplets, resulting in denser shells (Supplementary Fig. 33 and Supplementary Video 7). With DNA crosslinkers (A′, 8 bps; A_m_-A′_m_, 25 bps), the probability of finding structures in the interior of the droplets (*r*/*R* < 1) increases (Fig. 4e,f,h and Supplementary Fig. 34), probably due to DNA crosslinking. To selectively direct structures towards the cortex and interior, we pre-assembled FF and A_m_-A′_m_ (stained with thioflavin T (ThT) and Nile red, respectively) and mixed them into droplets. Confocal imaging and probability distribution analysis demonstrated that A_m_-A′_m_ structures typically exist in the interior of droplets, whereas FF fibres form elastic shells near the oil–water interface (Fig. 4g,h(iii), Supplementary Fig. 30 and Supplementary Video 6). These results suggest that the degree of bundling tuned by DNA crosslinkers is an effective handle for modulating structure enrichment in the droplet lumen or cortex.

### Confined cytoskeleton organization guides cargo diffusion and release

**Fig. 5:**
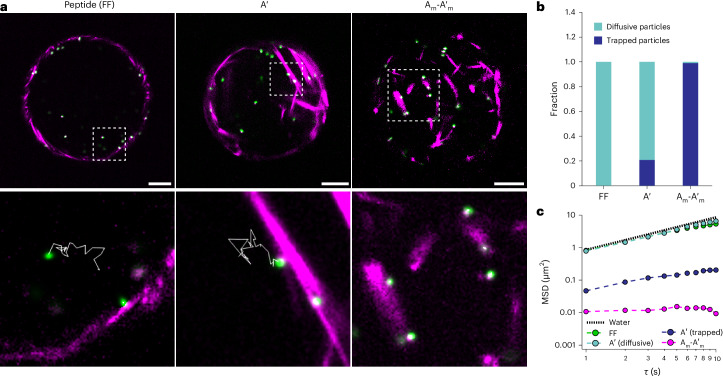
Diffusion of probe particles in peptide and peptide–DNA droplets. **a**, Top row: confocal images of particles in droplets containing FF, A′ or A_m_-A′_m_ (0.5 mol% peptide–DNA crosslinker) from an equatorial view of the droplets (green, 1-μm probe particle; magenta, peptide). Scale bars, 20 μm. Bottom row: magnified images of the white dashed boxes shown in the top row. The white solid lines show the particle trajectory over 30 frames at 1 frame s^−1^. **b**, Fraction of diffusive and trapped particles within droplets. **c**, MSDs of probe particles inside droplets containing FF, A′ or A_m_-A′_m_. *τ* is the lag time. In the case of A′, MSDs were calculated for both trapped and diffusive particles. The black dotted line shows the MSDs predicted for Brownian particles in water. MSDs are plotted on a linear scale in Supplementary Fig. 37. Source data

Cortical cytoskeleton can restrict protein and lipid diffusion and aid their segregation and transport, while cytoplasmic F-actin can affect passive transport^51^. As such, the cytoskeleton can modulate signal transduction and couple mechanical signals to biochemical responses. To probe the effect of DNA-crosslinked peptides on the cortex and intracellular dynamics of our synthetic cells, we incorporated 1 μm polystyrene particles into droplets containing FF, A′ or A_m_-A′_m_ (Fig. 5a). The probe particles freely diffuse through bulk peptide networks (Supplementary Video 8), suggesting minimal interaction with the peptides. Confocal imaging revealed two subpopulations of particles, those trapped in the cortex and those rapidly diffusing within the lumen, as shown by mean square displacement (MSD) analysis (Fig. 5c, Supplementary Video 9 and Supplementary Fig. 37). The FF particles in the interior have a diffusion coefficient (*D*_interior_(FF)) of 0.31 μm^2^ s^−1^, close to the theoretical prediction of Brownian particles in water (*D*_water_ ≈ 0.43 μm^2^ s^−1^), suggesting their free diffusion. As DNA crosslinkers were introduced, some particles were trapped, with the highest portion of particles trapped in bundled networks comprising longer DNA crosslinkers (A_m_-A′_m_; Fig. 5b and Supplementary Videos 10 and 11). These results indicate that the spatial organization of synthetic cytoskeletal structures at the periphery of the droplet or in the lumen guides the static diffusive properties of soluble cargo in confinement and might tune the viscoelasticity of the cell, akin to living cells^52,53^.

**Extended Data Fig. 3:**
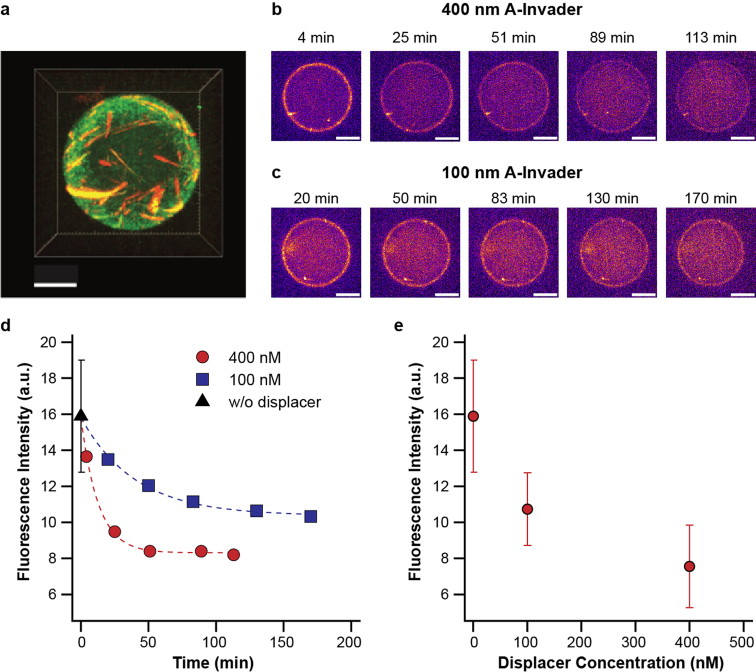
Controlled release of payloads from synthetic cortices through DNA displacement. (**a**) A 3D representation of FITC-A (green) hybridized onto A′ (0.5 mol%) synthetic cortices (red) in droplets. (**b-e**) Confocal time series of the release process of FITC-A from A′ using an invader DNA strand (A-I). (**b, c**) Time series of cross-sections of droplets during the release processes with (b) 400 nM and (c) 100 nM A-I. Images in the green channel (FITC) were falsely-colored after correcting the photobleaching effect and applying the same image contrast (see details in Supplementary Fig. 36). Scale bars: 10 μm. (**d**) Changes of fluorescence intensities over time on the interface of the droplets were plotted (Blue square: 100 nM displacer, Red circle: 400 nM A-I). Black triangle: We plotted an ensemble average of fluorescence intensities on A′ cortices without A-I as 0 min for comparison. The error bar represents the standard deviation (*n* = 8). Dotted lines are fitting results with exponential decay on fluorescence decay curves. Characteristic decay times are estimated to be 12.8 min and 40.2 min for 400 nM and 100 nM displacer, respectively. (**e**) The fluorescence intensities of FITC-A as a function of A-I concentration after displacement from the A′ cortices are plotted. The error bars represent ± SD over *n* = 8, 7, and 6 droplets for 0, 100, and 400 nM displacer. Source data

In addition to its ability to trap particles in the meshwork, native cytoskeleton can also provide transient docking sites for proteins in the membrane or cytoplasm^54^. To demonstrate the functionality of our peptide–DNA cytoskeleton as a polymeric hub to recruit signalling components, we added a strand of A modified with the dye fluorescein isothiocyanate (FITC-A) as a proxy payload that can bind a complementary DNA strand (A′) to our peptide–DNA networks and encapsulate them into droplets. Confocal imaging showed that FITC-A strands are co-localized on peptide–DNA at the cortex, indicating their attachment to A′ fibres (Extended Data Fig. 3a and Supplementary Figs. 35 and 36). To achieve controlled payload release from the peptide–DNA shells, we leveraged toehold-mediated strand displacement by adding a DNA invader (A invader (A-I)) to bind to a single-stranded toehold (comprising five bases; Supplementary Table 1) on FITC-A, eventually ‘peeling it off’ the A′ structure^55^. We introduced a low (100 nM) or high (400 nM) dose of A-I into A′–FITC-A droplets and monitored the release of FITC-A via live confocal imaging (Extended Data Fig. 3b,c, Supplementary Fig. 36 and Methods), plotting the average fluorescence intensity at the cortex over time (Extended Data Fig. 3d). To obtain the initial fluorescence, we calculated the average fluorescence intensity of several droplets in the absence of A-I. Our analysis revealed a decay in the fluorescence intensity and increase in the decay constant with increasing A-I concentration (Extended Data Fig. 3e), demonstrating that DNA strand displacement effectively controls the kinetics of payload release.

Together, these results highlight how the structural tunability afforded by the DNA crosslinkers allows us to mimic the spatiotemporal organization of signals within synthetic cells, which is an essential function of cells. We have shown that the peptide–DNA cytoskeleton can mimic two vital properties of actin self-organization: (1) the trapping and localized confinement of cargo diffusion and (2) the scaffolding and targeted release of bound signals along polymer networks. These two fundamental properties lay the groundwork for connecting the spatial organization of our synthetic cytoskeleton to tuning signalling pathways in the future.

### Shaping synthetic cells with tunable cytoskeleton hierarchy

**Extended Data Fig. 4:**
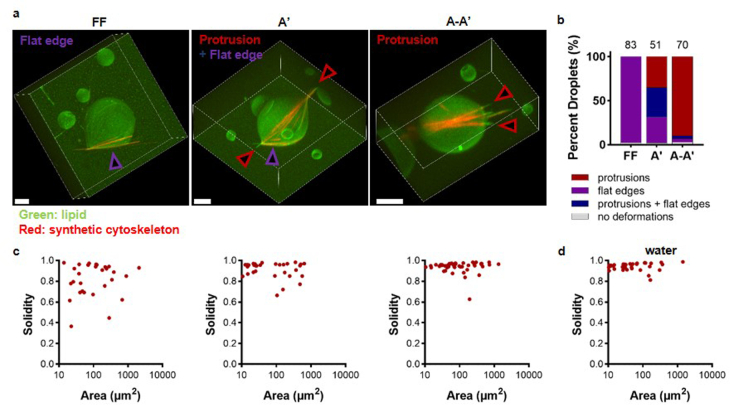
Cell phenotypes with tunable synthetic cytoskeletons. (**a**) 3D views of confocal images of one-step lipid-oil droplets containing FF, A′ and A-A′ at 1 mole % crosslinker (green channel: Atto488-DOPE, red channel: NileRed-stained peptide or peptide–DNA). Scale bars: 5 μm. Images are labeled with the deformation types ‘flat edge’ (purple) and ‘protrusion’ (red). (**b**) Percentage of lipid-oil droplets containing FF (*n* = 83), A′ (*n* = 51) and A-A′ (*n* = 70) qualitatively classified by deformation types. (**c**) Solidity (area÷convex hull) plotted against area, quantified from the lipid channel of droplets containing (c) FF (*n* = 30), A′ (*n* = 33), A-A′ (*n* = 47) and (d) water (*n* = 37). Source data

The spatiotemporal hierarchical organization of actin, facilitated by actin-binding proteins, governs its protrusive behaviour, thereby controlling cell shape^56^. To study the effect of peptide–DNA networks and bundles on lipid-coated droplet shape, we used a one-step approach (Supplementary Fig. 38) and dissolved lipids (1,2-dioleoyl-*sn*-glycero-3-phosphoethanolamine (DOPE) with 10% Atto488-DOPE) in oil, and then added aqueous pre-assembled peptide–DNA structures (FF, A′ or A-A′) to the lipid–oil mixture (Supplementary Fig. 38). DOPE was used as it has previously been shown to increase membrane fluidity and therefore may enhance the propensity for droplet deformation^57,58^. Confocal imaging revealed average lipid droplet diameters of ~15 μm with deformed shapes (Supplementary Figs. 39 and 40). Shape deformation analysis revealed that the FF lipid droplets exhibit more non-protrusive flat-edge deformations, while the addition of DNA crosslinkers induced filopodia-like protrusions (Extended Data Fig. 4 and Supplementary Fig. 39c,d). A′ formed large protruding bundles at the droplet mid-plane and thin bundles at the membrane edge, whereas the larger bundles in A-A′ yielded multiple spiky filopodial protrusions. These results resemble those of reconstituted actin in lipid vesicles, where a short actin crosslinker (fascin) guides the formation of filopodia-like protrusions, while a long crosslinker (α-actinin) impairs them^7,8^. Altogether, the modularity of the actin-mimetic peptide–DNA framework can advance the design of shape-changing synthetic cells.

**Fig. 6:**
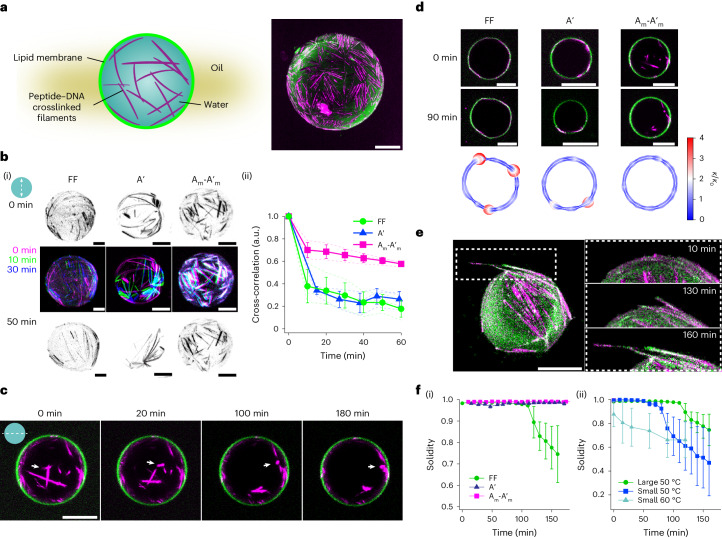
Shaping of synthetic cells using peptide–DNA cytoskeletons. **a**, Schematic (left) and confocal maximum intensity projection (right) of peptide–DNA cytoskeleton (0.5 mol% A′) encapsulated in lipid droplets (green, Atto488-DOPE; magenta: A′ stained with Nile Red). Scale bar, 20 μm. **b**, Cytoskeletal reorganization on the cortex upon heating. (i) Filament organization at 0 min (top) and after 50 min (bottom) of heating and overlaid maximum intensity projections (middle) of the red channel (Nile red, peptide and peptide–DNA structures) from time lapses of lipid droplets containing FF (left), A′ (middle) and A-A′ (right) heated at 50 °C (red, image at 0 min; green, image at 10 min; blue, image at 30 min). Scale bars, 10 μm. (ii) Cross-correlations of binarized maximum *z* projections (red channel) versus time during heating. Data for individual droplets and their averages are depicted as dashed lines and markers, respectively. Data are presented as the mean ± s.d. for *n* = 3 droplets for FF, A′ and A_m_-A′_m_. **c**, Equatorial plane confocal images of lipid droplets containing A_m_-A′_m_ after heating to 50 °C over time, showing that bundles decluster and migrate towards the droplet shell, indicated by the white arrows. Scale bar, 20 μm. **d**, Local curvature analysis of peptide–DNA lipid droplets containing FF, A′ or A_m_-A′_m_ under deformation. The images represent cross-sections of droplets before and after heating at 50 °C for 90 min. The contour traces of the cross-sections of the lipid droplets after heating at 50 °C for 90 min are represented by white solid lines. The ratios of their local curvatures (*κ*) over the global curvatures of the contour obtained by circular fitting (*κ*_0_) are plotted along the contour in the form of circles. Circle sizes and colours reflect the absolute values of *κ*/*κ*_0_. Scale bars, 20 μm. **e**, Maximum intensity projections of a deformed lipid droplet containing FF after heating at 50 °C for 2 h (left). Magnified images (right) of the area highlighted in the left-hand image showing the formation of a protrusion over time (Supplementary Video 13). Scale bar, 20 μm. **f**, Evolution of solidity over time (area ÷ convex hull) for FF, A′ and A_m_-A′_m_ lipid droplets with diameters above 25 μm heated to 50 °C (i) and FF lipid droplets with diameters above (large) and below (small) 25 μm heated to 50 and 60 °C (ii). The evolution of other representative droplets on heating is shown in Supplementary Videos 13–15 and Supplementary Fig. 42. The error bars in **f**(i,ii) represent the mean ± s.d. for *n* = 13 droplets for FF, *n* = 8 droplets for A′ and *n* = 10 droplets for A_m_-A′_m_. Source data

Next, we attempted to generate lipid droplets while preserving the spatial distribution of structures across the cortex and lumen. To do so, we implemented a two-step approach (Supplementary Fig. 38), first forming membraneless water-in-oil droplets with peptides crosslinked by different DNAs and then subsequently adding lipid–oil. Indeed, confocal microscopy revealed the successful lipid coating of droplets while maintaining the integrity and localization of cytoskeletal structures (Fig. 6a) with minimal changes in the droplet shape, regardless of size (Supplementary Fig. 41). Having achieved lipid droplets reinforced with different cytoskeletal networks, we explored their response to environmental stresses, including elevated temperatures. When exposed to stress, living cells drastically modify their cytoskeletal networks^59,60^. It has been shown that stabilizing actin crosslinking can protect cells and attenuate injury^61^. Inspired by this, we explored how distinct spatial organization into the cortex and lumen of droplets will affect their response to heat. Subjecting droplets to 50 °C (Supplementary Videos 12–15 and Supplementary Fig. 42) induced two types of behaviour: (1) the reorganization of cytoskeletal structures (Fig. 6b,c) and (2) the deformation and emergence of filopodia-like protrusions (Fig. 6d–f). The type and extent of these heat-induced behaviours depend on the spatial localization and degree of synthetic cytoskeleton crosslinking.

To evaluate the dynamics of the cortical filaments, we compared the cross-correlations of binarized maximum *z* projections of shells throughout the heating process (Fig. 6b(ii), Supplementary Fig. 42 and Supplementary Video 12). For the weakly crosslinked systems FF and A′, significant filament translocation was observed in the cortex upon heating (Fig. 6b(ii)), while the movement of A_m_-A′_m_ in the cortices was minimal. The spatial correlation of filaments along the *z*-sliced cortex contour (defined by the coordinate *s*), *C*(*s*) (Supplementary Fig. 43), indicates an increased correlation length (*ξ*_c_) for weakly crosslinked droplets (FF and A′), with the overall correlation decreasing upon heating, indicating the clustering of thin bundles (Supplementary Fig. 44) and reduced coverage of the cortex by filaments. In contrast, no change in the *C*(*s*) of A_m_-A′_m_ droplets was observed upon heating, suggesting that DNA base pairing reinforces the shell network. Moreover, large bundles in the lumen of A_m_-A′_m_ droplets were observed to decluster with heating, as shown by time series cross-sectional confocal images (Fig. 6c and Supplementary Fig. 42c). This was further confirmed by the decrease in the probability of large bundles inside the droplets upon heating, as shown by the probability distribution analysis (Supplementary Fig. 42g). This structural reconfiguration is probably stimulated by the melting of weak crosslinks, consistent with the heat-reversible mechanics of bulk networks (Fig. 3). Following declustering, fibres translocate and adhere to the oil–water interface, possibly due to their hydrophobic nature.

The structural reorganization of the peptide–DNA cortex further induces droplet deformations, depending on their size (Supplementary Videos 13–15). For large droplets (≳25 μm), the global sphericity (*D*_in_/*D*_out_) estimated from maximum *z* projections remained unchanged at ~1 (Fig. 6d and Supplementary Fig. 42e) for the first 2 h. However, the heat-induced fibre reorganization caused locally high curvatures of the cortices (Fig. 6d and Supplementary Fig. 45) that resulted in filopodia-like protrusions. The effect is particularly evident in FF droplets, less in A′ and minimal for A_m_-A′_m_. This can be attributed to the varying filament lengths of the different DNA crosslinkers, as FF fibres are the longest and require the highest bending energy for encapsulation in cell-sized confinement. The solidity (measure of compactness) of the FF lipid droplets remained close to 1 for 2 h at 50 °C, indicating a solid and compact structure (Fig. 6f(i)). Prolonged heating sharply reduced the solidity of the FF droplets to ~0.7 (less compact) due to fibre protrusion, minimizing local membrane deformations and releasing the fibre-bending energy^62^. In contrast, the fibres in A′ and A_m_-A′_m_ droplets do not protrude, probably due to their shorter length (lower bending energy of fibres), generating smaller local membrane deformations (Supplementary Fig. 31). For small FF droplets (≲25 µm), which are globally deformed before heating (Supplementary Fig. 42f), fibres protruded earlier in the heating process (Supplementary Video 13) and the solidity analysis revealed enhanced protrusion. This protrusion behaviour was more pronounced at higher temperatures (Fig. 6f(ii)). Smaller A′ droplets exhibited deformations and fibre protrusions upon heating (not observed for larger A′ droplets), as shown by the solidity and circularity *D*_in_/*D*_out_ analyses (Supplementary Fig. 42f and Supplementary Video 14). These enhanced deformations in smaller droplets are probably caused by the higher fibre curvatures resulting from their encapsulation in smaller droplets. For smaller A_m_-A′_m_ droplets, no deformations were observed, probably due to the shorter fibre length (Supplementary Fig. 42f and Supplementary Video 15).

Together, our experiments demonstrate that synthetic cytoskeletal networks constructed with peptide–DNA nanotechnology and confined in lipid droplets can reinforce cells differently and undergo dynamic reorganization on heating, replicating natural membrane–cytoskeleton interactions.

## Conclusions

We have established a tunable synthetic cytoskeleton by integrating DNA nanotechnology with self-assembled peptides in cell-sized confinement. Inspired by actin-associated proteins and leveraging DNA base pairing programmability, we designed peptide–DNA crosslinkers of varying length and geometry. The crosslinkers organize nanofilaments into higher-order tactoid-shaped bundles with DNA-tunable aspect ratios and mechanical properties, recreating cytoskeleton polymorphism in a purely synthetic supramolecular system. Confining peptide–DNA networks within water-in-oil droplets generated cytoskeleton-reinforced synthetic cells in which fibres formed cortex-like shells and large bundles localized within droplets. The spatial organization of the peptide–DNA cytoskeletons, guided by the associated crosslinkers, fine-tuned the passive diffusion of micrometre-sized payloads within the droplets and enabled the targeted recruitment of DNA-modified signals onto complementary DNA anchors. Encapsulating peptide–DNA within lipid-coated droplets generated cell-like deformations and filopodia-like protrusions. Finally, environmental stresses such as heat induced the cytoskeletons to reorganize and reshape the droplets. This modular peptide–DNA platform allows the plug and play of alternative peptide assemblies or more complex DNA designs to yield emerging functional morphologies. Altering polymerization conditions, or combining with additional chemistries, may further tune the internal dynamics of the rigid tactoids towards a more liquid-like behaviour. It can easily interface with the growing activities of DNA-based synthetic cells^11^ through specific DNA inputs, strand displacement reactions^55^ or with native proteins and enzymes to form and reconfigure composite networks^63^. We envision the ability to further fine-tune cell shape by varying the lipid composition to alter the membrane properties of peptide–DNA synthetic cells^56^. The peptide–DNA library can be expanded to interface with aptamer-based logic computing reactions^64,65^, biomolecular condensates^66^, light or pH^67^. We expect designer peptide–DNA architectures to enable the introduction of emergent properties into synthetic cells and enhance their functionality.

*Note added in proof*: During processing of this paper, a related paper by Fu et al. appeared online in *Nature* reporting on the supramolecular formation of tactoids with a ureidopyrimidinone-based system^68^. This is in line with our findings, and further amplifies the power of supramolecular polymerization to produce emergent structures and function.

## Methods

### Materials

All chemicals were used without further purification. Rink amide 4-methylbenzhydrylamine hydrochloride resin and Fmoc-Lys(azide)-OH were purchased from Chem-Impex. Fmoc-(PEG)_2_-OH (PEG, polyethylene glycol) was purchased from PurePEG. Fmoc-Phe-OH, *N*,*N*′-diisopropylcarbodiimide, trifluoroacetic acid (TFA), triisopropylsilane (TIPS) and triethylammonium acetate (TEAA) were purchased from Sigma-Aldrich. Ethyl cyano(hydroxyimino)acetate (Oxyma Pure) and piperidine were purchased from VWR. Dibenzocyclooctyne-sulfo-*N*-hydroxysuccinimidyl ester (DIBAC-sulfo-NHS) was purchased from Sigma-Aldrich; it was dissolved in dry DMSO to a final concentration of 100 mM and stored at −20 °C before use. All DNA strands (including amine-modified strands) were purchased from IDT; they were dissolved in water to a final concentration of 1 mM or 100 μM and stored at −20 °C before use. Fmoc-FF-OH and Fmoc-Phe-Phe-PEG_2_-Lys(FITC)-NH_2_ were purchased from Bachem.

### Peptide synthesis

Fmoc-FF-(PEG)_2_-K_az_-NH_2_ was synthesized by automated Fmoc solid-phase peptide synthesis (Liberty Blue, CEM) on rink amide 4-methylbenzhydrylamine resin (100–200 mesh, 0.77 mmol g^−1^). The peptide was cleaved from the resin in a solution of 95% TFA, 2.5% TIPS and 2.5% deionized H_2_O. The cleaved peptide solution was concentrated and resuspended in a 3:1 mixture of acetonitrile and 0.1% TFA–H_2_O. The solution was purified by reversed-phase HPLC (Shimadzu UFLC, Ultra C18 5 µm, 100 mm × 10 mm column) using a gradient of 0.1% TFA–H_2_O (solvent A) and acetonitrile (solvent B). The purity of the peptide was confirmed by electrospray ionization mass spectrometry (ESI-MS; Q-exactive HF-X).

### Peptide–DNA synthesis

Amine-modified oligonucleotides (1 mM or 100 μM in H_2_O; Supplementary Data 1) were diluted in 20 mM sodium phosphate buffer (pH 8.5). DIBAC-sulfo-NHS (100 mM in DMSO) was added to the solution in excess, and the mixture allowed to react for 2 h with vigorous shaking at room temperature. The solution was reactivated with DIBAC-sulfo-NHS and gently shaken overnight at 4 °C. The solution was then exchanged with 100 mM sodium phosphate buffer (pH 7.5). Fmoc-FF-(PEG)_2_-K_az_-NH_2_ was dissolved in DMSO to a final concentration of 2 mM and added in a 2:1 molar ratio to the DNA–DIBAC solution. Water and DMSO were added to bring the reaction mixture to 20 mM phosphate buffer (pH 7.5) and 20% DMSO. The reaction mixture was then shaken overnight at room temperature. The solution was exchanged with 50 mM TEAA buffer (pH 7.0), concentrated and purified by reversed-phase HPLC (Inspire C18 5 µm, 250 mm × 4.6 mm column) with a 5–80% gradient of 50 mM TEAA buffer (pH 7.0) in H_2_O (solvent A) and 90% acetonitrile in water with 50 mM TEAA buffer (pH 7.0; solvent B). The identity and purity of the peptide–DNAs were confirmed by ESI-MS (ThermoScientific Q Exactive HF-X) and matrix-assisted laser desorption/ionization MS (AB Sciex 5800 MALDI-TOF/TOF). The peptide–DNAs were quantified by absorbance measurements at 260 nm, lyophilized in aliquots of 0.5–2 nmol and stored at −20 °C.

### Peptide and peptide–DNA assembly

We assembled the materials in either 1% DMSO–water or water. For assemblies in DMSO–water (DMSO switch method), we prepared a concentrated stock solution of 10 wt% Fmoc-FF-OH (Bachem) in DMSO, and then diluted it to 0.1 wt% in 10 mM potassium phosphate buffer (pH 7.5) with 150 mM NaCl. For co-assemblies of peptide and peptide–DNA, the peptide solution was used immediately to dissolve the required amount of lyophilized peptide–DNA. The solutions were then heated at 95 °C for 25 min, while mixing (by snapping) around every 8 min, followed by annealing (on a thermocycler or rheology plate) from 80 to 25 °C at a cooling rate of −1 °C min^−1^. For assemblies in water, Fmoc-FF-OH was dissolved in 10 mM potassium phosphate buffer (pH 7.5) with 150 mM NaCl and horn-sonicated on ice three times for 5 s each. For peptide–DNA co-assemblies in water, Fmoc-FF-OH was dissolved in 10 mM phosphate buffer (pH 7.5) with 150 mM NaCl and then the peptide solution was used to solubilize the lyophilized peptide–DNA. The solutions were horn-sonicated on ice three times for 5 s each to mix the monomers and then annealed from 95 to 25 °C at a cooling rate of −1 °C min^−1^. All samples were stored at either 4 °C or room temperature and used up to 1 month after assembly, checking for the absence of larger aggregates by eye and for consistency between samples by microscopy.

### CD spectroscopy

CD measurements were collected on a Chirascan Plus spectropolarimeter using a 1 mm path length cuvette under a controlled temperature. Peptide and peptide–DNA samples prepared in DMSO–water (typically at 1.87 mM, 0.1 wt%) were diluted 1:3 with water for measurement. Spectra were recorded in the range of 220–320 nm.

### Transmission electron microscopy

First, 10 μl of sample was spotted onto a carbon-coated 300 mesh copper grid (Electron Microscopy Sciences). After 5 min, the sample was wicked from the grid. The grid was then washed twice with water and stained twice with 2% uranyl acetate for 20 s. The samples were imaged on a FEI Tecnai T12 microscope at 120 kV. Images were analysed and processed with minor adjustments for brightness and contrast using ImageJ software (National Institutes of Health). Alignment fraction analysis was performed using the OrientationJ Analysis plugin on ImageJ.

### Confocal microscopy of peptides and peptide–DNA assemblies (DMSO switch method)

A Nile red stock solution (10 mM in DMSO) was diluted with 10 mM potassium phosphate buffer (pH 7.5) containing 150 mM NaCl to 20 µM and peptide and peptide–DNA structures were assembled as described above. The samples were then sandwiched between two coverslips using strips of double-sided sticky tape and sealed with mineral oil to prevent evaporation. The sample chambers were annealed from 80 °C (or 60 °C) to 25 °C at a cooling rate of −1 °C min^−1^ unless stated otherwise.

Samples were imaged with either an Andor XD spinning disk confocal microscope with a CSU-X1 Yokogawa head or a Zeiss LSM 880 scanning microscope. In the case of the Andor XD spinning disk microscope, the system was equipped with a complementary metal–oxide–semiconductor (CMOS) camera (Hamamatsu Flash4v2 sCMOS). The samples were excited with a 488 or 561 nm laser and imaged with a ×20 or ×60 objective (Olympus 20X/0.75 UPlan S-APO and 60X/1.20 Water UPlan S-APO). *z* Stacks were obtained with steps of either 0.4 or 1 μm. For the heating experiments on the annealed A′ sample (Supplementary Video 2), the sample chambers were heated at 60 °C on the heating plate for 10 min and then placed in the microscope at room temperature to monitor the evolution of the samples.

We found that the samples were inhomogeneously illuminated in the case of the Andor XD spinning disk microscope. To correct the effect of inhomogeneous illumination, an image of the homogeneous dye solution was collected and then normalized by the average pixel intensity of the image after background subtraction to create a map of normalization factors for each pixel. The effect of inhomogeneous illumination was corrected by subtracting the background of the sample images and dividing them by the map of normalization factors.

In the case of the Zeiss LSM 880 scanning microscope, the microscope was equipped with ×20, ×40 and ×63 objectives (Zeiss 20X/0.8 Plan-Apochromat, 40X/1.30 Plan-Neofluar and 63X/1.4 Plan-Apochromat). The samples were excited with a 488 or 561 nm laser for green and red fluorescence, respectively. *z* Stacks were obtained with steps of 0.5–1 μm, depending on the sample.

### Acridine orange confocal microscopy

To examine the local effect of DNA binding on bundle formation, we stained the peptide–DNA materials with acridine orange. As acridine orange emits red fluorescence when bound to single-stranded DNA (ssDNA; *λ*_ex_ = 457 nm and *λ*_em_ = 638 nm) and green fluorescence when bound to double-stranded DNA (dsDNA; *λ*_ex_ = 502 nm and *λ*_em_ = 522 nm), it allowed us to estimate the degree of DNA hybridization localized in the bundles by measuring the fluorescence intensity in these two spectral regions. To prepare samples for confocal microscopy, 1.2 μl of 50 μM acridine orange solution was added to 3 μl peptide–DNA solutions. The samples were incubated at room temperature for at least 3 h. The samples were then sandwiched between two coverslips using strips of double-sided sticky tape and the chambers were sealed using nail polish. All of the experiments were performed at room temperature. Fluorescence images of the peptide–DNA bundles were recorded using the Zeiss 880 confocal microscope with the ×63 objective. Fluorescence excitation was performed with the 488 nm laser. The excitation light intensity was kept constant throughout the measurement. To collect the green and red fluorescence separately, the emission ranges of the detector were set to 490–553 and 623–680 nm, respectively. Although acridine orange is known as a nucleic acid binding dye, we found that it also binds to Fmoc-FF, emitting both green and red fluorescence (Supplementary Fig. 17). To see the effect of DNA binding, we subtracted the effect of Fmoc-FF from images and obtained information on DNA base pairing. Assuming that the fluorescence intensities of each channel are a linear combination of the fluorescence sources, we defined *I*_green_ = *I*_dsDNA_ + *I*_green,FF_ and *I*_red_ = *I*_ssDNA_ + *I*_red,FF_, where *I*_green_ and *I*_red_ are the fluorescence intensities of the green and red channels of each pixel, respectively, *I*_dsDNA_ and *I*_ssDNA_ are the fluorescence intensities of dsDNA (green channel) and ssDNA (red channel), respectively, and *I*_green,FF_ and *I*_red,FF_ are the background green and red fluorescence intensities generated by non-specific peptide–acridine orange interactions, respectively. To subtract the contributions of the peptide, we defined the ratio of the peptide fluorescence intensities of the green and red channels, *β* = *I*_green,FF_/*I*_red,FF_, obtained from imaging the peptide fibres (Supplementary Fig. 17) and derived *I*_green_ − *βI*_red_ = *I*_dsDNA_ − *βI*_ssDNA_, which relates *I*_green_ − *βI*_red_ to the difference in the local concentrations of dsDNA and ssDNA.

### Rheology measurements on peptide and peptide–DNA assemblies

Rheology experiments were performed using a commercial rheometer (AR-G2, TA Instruments) in oscillatory mode using a 40-mm aluminium parallel plate. Peptide and peptide–DNA solutions were prepared using the DMSO switch method as described above and placed on the rheometer plate at 80 °C (or 60 °C). We applied a thin layer of low-viscosity mineral oil around the sample to minimize evaporation. The annealing process from 80 °C (or 60 °C) to 25 °C at a cooling rate of −1 °C min^−1^ was monitored at a frequency of 1 Hz with 0.5% strain. Frequency sweep measurements were collected on annealed samples from 0.1 to 50 Hz with 0.5% strain, which is in the linear viscoelastic range of peptide–DNA, as shown by strain sweep measurements (Supplementary Fig. 23). To examine the temperature dependence of the rheological properties, temperature sweep experiments were performed at a frequency of 1 Hz with 0.5% strain. For A′, the temperature was changed from 25 °C to 40 °C back to 25 °C. This procedure was repeated twice. Then the temperature was changed from 25 °C to 50 °C back to 25 °C. For A-A′, the temperature was changed from 25 °C to 50 °C back to 25 °C. For A-B-C, the temperature was changed from 25 °C to 40 °C back to 25 °C and then from 25 °C to 50 °C back to 25 °C.

### Peptide and peptide–DNA stabilized water-in-oil droplet formation

First, 2 μl assembled peptide or peptide–DNA fibre (0.5 mol% DNA, peptide stained with Nile red or ThT) solution was added together with 10–20 μl mineral oil into a test tube. The tube was then flicked until cloudy (ten times) and left to stand at room temperature for 5 min. The sample was flicked again ten times until cloudy (cloudiness is an important observation for determining whether the solution has been properly mixed to form an emulsion) and immediately mounted on a coverslip using strips of double-sided sticky tape as a spacer, with another coverslip positioned on top for confocal imaging. Coverslips for imaging were siliconized (SurfaSil, Thermo Fisher) before mounting the sample to avoid droplet attachment to the glass surface. For the two-colour droplets in Fig. 4, FF and DNA-crosslinked structures were separately assembled with either ThT or Nile red (dyes capable of staining both peptides and DNA assemblies) to distinguish between structures in the cortex and lumen.

### Microsphere diffusion in droplets

First, 1-μm green microspheres with surface carboxy groups (F8823, Invitrogen) were mixed with solutions of assembled fibres and then mixed with mineral oil as described above. The resultant solutions were introduced into the sample chamber, which consisted of two coverslips sandwiched together. To assess the distribution of probe particles within the droplets, *z* stacks of droplets were acquired using the Andor XD spinning disk confocal microscope, capturing both green and red channels. The dynamics of particles at the equator plane of the droplets were then recorded with a frame rate of 1 frame s^−1^ for 100 s.

### Conjugation of fluorescent dye to DNA

Amine-modified oligonucleotide, A-NH_2_, was dissolved in 50 mM MES buffer (pH 6; M8250, Sigma) at a concentration of 0.2 mM. Then, 2 μl of a 10 mg ml^−1^ solution of FITC in DMSO was added to 20 μl of the A-NH_2_ solution. Next, 20 μl of 8 mg ml^−1^ 1-ethyl-3-[3-(dimethylamino)propyl]carbodiimide was added to the mixture, which was then incubated on a shaker overnight at room temperature. Unreacted FITC was removed by centrifugation using an Amicon Ultra-0.5 3K centrifugal filter unit (UFC5003, Sigma) at 10,000*g* more than 10 times for 30 min each.

### Displacement of FITC-A from A′ filaments in droplets

To hybridize FITC-A to A′ networks, 1 μl of a 50-fold diluted FITC-A solution was added to 3 μl of assembled A′ fibre solution and then incubated for at least 30 min (for DNA sequences, see Supplementary Data 1). The resultant fibres were encapsulated in water-in-oil droplets as described in the previous section. *z* Stacks of droplets with a *z* spacing of 1 μm were acquired with an Andor XD spinning disk confocal microscope equipped with the ×60 objective.

For the controlled release of FITC-A from A′ fibres, an A-I strand was added to the solution of A′ fibres hybridized with FITC-A. We tested two concentrations of A-I, 100 and 400 nM. The resultant DNA–fibre mixtures were encapsulated in water-in-oil droplets. *z* Stacks of droplets with a size of ~30 μm were acquired over time with a *z* spacing of 1 μm to observe the release process.

Continuous imaging of the same droplets during the release process may result in photobleaching, which can lead to an artificial reduction in fluorescence intensity. This effect was taken into consideration as follows. A solution of A′ fibres hybridized with FITC-A was encapsulated in water-in-oil droplets. *z* Stacks of droplets with a size of ~30 μm, similar in size to those imaged for the release process, were acquired five times with the same laser intensity and *z* spacing as in the release process. The acquired images (Supplementary Fig. 36) were used to quantify the photobleaching effect, which was corrected for in the images presented in Extended Data Fig. 3 (Supplementary Fig. 36).

### Lipid–oil mixture

DOPE (Avanti Polar) and Atto488-DOPE (Sigma-Aldrich) were dissolved in chloroform in glass vials at concentrations of 5 and 1 mg ml^−1^, respectively. These stock solutions were stored at −20 °C. DOPE was used because of its unsaturated fatty acid chains, which increase membrane fluidity, thus enhancing the potential for membrane deformation^57,58^. Next, 45 μl of the DOPE–chloroform solution was mixed with 25 μl of the Atto488-DOPE–chloroform solution. The total weight of lipids was 250 μg with a weight ratio of DOPE/Atto488-DOPE of 9:1. The chloroform in the lipid solution was evaporated under vacuum for at least 2 h. The thin lipid film formed at the bottom of the vial was dissolved in 1 ml mineral oil and sonicated in a water bath for 1 h to ensure homogeneous dissolution.

### Formation of lipid-stabilized water-in-oil droplets

To form lipid-stabilized water-in-oil droplets, we encapsulated the peptide or peptide–DNA structures into lipid–oil droplets using two methods. In the first method (one step), 10 μl of the DOPE–Atto488-DOPE oil solution (as described above) was placed in a 200-μl plastic tube. Then, 2 μl of the aqueous solution (assembled peptide, peptide–DNA or water) was layered on top of the lipid–oil solution. The tube was then flicked ten times (until turbid), left to stand at room temperature for 5 min and then flicked again ten times until turbid. The sample was immediately mounted between two siliconized coverslips using double-sided sticky tape as a spacer for imaging, as described above. In the second method (two steps), the fibre solutions were first mixed with mineral oil without the lipids to prepare droplets, as described above. Then, 2 μl of the oil–water mixture was placed on the coverslip and covered with another coverslip using double-sided sticky tape strips as spacers. Then, the lipid–oil solution was introduced from the side of the chamber.

Samples were imaged with the Andor XD spinning disk confocal microscope or the Zeiss 880 microscope, as described above. The samples were excited with 488 and 561 nm lasers for green and red fluorescence, respectively. For the heating experiments on droplets, the sample chambers composed of double-sided sticky tape strips sandwiched between two coverslips were adhered to glass slides, placed on the heating stage of the Zeiss 880 microscope and heated to 50 or 60 °C. To monitor the evolution of the samples, *z*-stack images were collected about every 10 min when heating to 50 °C and at 15, 30, 60, 90 and 120 min when heating to 60 °C.

### Bending stiffness measurement of FF fibres using fluorescence microscopy

We used the DMSO switch method to assemble 0.1 wt% Fmoc-FF fibres, which we then briefly sonicated in a water bath to reduce the fibre length to estimate their bending stiffness. The assembled FF fibres were stained with 50 μM ThT and then mixed with a concentrated sucrose solution to slow the dynamics of the fibres. The final sucrose concentration was 50 wt%. Using an Olympus IX-81 inverted microscope with ×100 magnification (Olympus UPLFLN ×100/1.3 objective) and a charge-coupled device (CCD) camera (Hamamatsu ORCA-R2 cooled CCD camera), green fluorescence images of the FF fibres were captured at a frame rate of 2.3 frames s^−1^. The imaged FF fibres had a length of 5–7 μm.

The bending stiffness of the FF fibres can be determined from the amplitude of shape fluctuations, as shown in previous studies on biopolymers, carbon nanotubes and DNA nanotubes^69–71^. The coordinates (*x*_*i*_, *y*_*i*_) of the backbones of the FF fibres were extracted from each image using the FIESTA plugin in MATLAB^72^ and used in further data analysis. The contours were quantified by the transverse deflection *u*(*s*,*t*) with contour length variable *s* and time *t* and then expressed as the sum of orthogonal dynamic eigenmodes, *y*_*q*_(*s*): $u\left(s,t\right)={\sum }_{q}{a}_{q}(t){y}_{q}(s)$ with the wavenumber *q* = *α*_*k*_/*L* = (*k* + ½)/*L*, where *a*_*q*_(*t*) is the variance of the amplitude, *k* = 1,2,3… is the mode number and *L* is the fibre length. For the free-end boundary condition, the eigenfunctions *y*_*q*_(*s*) are given by^73^

$${y}_{q}(s)=\frac{1}{\sqrt{L}}\left\{\frac{\cosh \left[q\left(s-L/2\right)\right]}{\cosh \left({\alpha }_{k}/2\right)}+\frac{\cos \left[q\left(s-L/2\right)\right]}{\cos \left({\alpha }_{k}/2\right)}\right\}{\rm{if}}\;{k\; {\rm{odd}}}$$

$${y}_{q}\left(s\right)=\frac{1}{\sqrt{L}}\left\{\frac{\sinh \left[q\left(s-\frac{L}{2}\right)\right]}{\sinh \left(\frac{{\alpha }_{k}}{2}\right)}+\frac{\sin \left[q\left(s-\frac{L}{2}\right)\right]}{\sin \left(\frac{{\alpha }_{k}}{2}\right)}\right\}{\rm{if}}\;k\;{\rm{even}}.$$

The bending stiffness of FF fibres can be estimated by analysing the *q* dependence of the variance of the amplitude *a*_*q*_(*t*). In this study, we evaluated the amplitude *a*_*q*_(*t*) based on the local tangent angle of the filament *θ*(*s*_*i*_) = tan^−1^[(*y*_*i*+1_ − *y*_*i*_)/(*x*_*i*+1_ − *x*_*i*_)] via integration by parts, ${a}_{q}(t)={\int }_{0}^{L}{dsu}(s,t){y}_{q}(s)=-{\int }_{0}^{L}{ds}\theta (s,t){\tilde{y}}_{q}(s)$, based on the following approximation, ∂*u*(*s,t*)/∂*s* ≈ *θ*(*s,t*)^74^. ${\tilde{y}}_{q}(s)$ represents the integral of *y*_*q*_(*s*), which is given by

$${{{\tilde{y}}}}_{q}(s)=\frac{\sqrt{L}}{{\alpha }_{k}}\left\{\frac{\sinh \left[q\left(s-L/2\right)\right]}{\cosh \left({\alpha }_{k}/2\right)}+\frac{\sin \left[q\left(s-L/2\right)\right]}{\cos \left({\alpha }_{k}/2\right)}\right\}{\rm{if}}\;k\;{\rm{odd}}$$

$${{{\tilde{y}}}}_{q}(s)=\frac{\sqrt{L}}{{\alpha }_{k}}\left\{\frac{\cosh \left[q\left(s-L/2\right)\right]}{\sinh \left({\alpha }_{k}/2\right)}+\frac{\cos \left[q\left(s-L/2\right)\right]}{\sin \left({\alpha }_{k}/2\right)}\right\}{\rm{if}}\;k\;{\rm{even}}.$$

## Online content

Any methods, additional references, Nature Portfolio reporting summaries, source data, extended data, supplementary information, acknowledgements, peer review information; details of author contributions and competing interests; and statements of data and code availability are available at 10.1038/s41557-024-01509-w.

### Supplementary information

Supplementary Information Supplementary Discussions 1 and 2, Figs. 1–45 and Tables 1 and 2.

Supplementary Data 1 Sequences of oligonucleotides used in this study.

Supplementary Video 1 Confocal microscopy time lapse of peptide fibres for bending stiffness calculations.

Supplementary Video 2 Confocal microscopy time lapse of peptide–DNA at elevated temperatures. A′ was heated to 60 °C, then imaged on a stage at room temperature. The video was taken up to 10 min after the sample had started to cool. The duration of each movie was 30 s.

Supplementary Video 3 Confocal microscopy of the FF droplet through *z* slices. Confocal microscopy images of a droplet containing FF, shown as a progression through the *z* planes of the droplet.

Supplementary Video 4 Confocal microscopy of the A′ droplet through *z* slices. Confocal microscopy images of a droplet containing A′, shown as a progression through the *z* planes of the droplet.

Supplementary Video 5 Confocal microscopy of the A_m_-A′_m_ droplet through *z* slices. Confocal microscopy images of a droplet containing A_m_-A′_m_, shown as a progression through the *z* planes of the droplet.

Supplementary Video 6 Confocal microscopy of the FF + A_m_-A′_m_ droplet through z slices. Confocal microscopy images of a droplet containing FF and A_m_-A′_m_, shown as a progression through the *z* planes of the droplet.

Supplementary Video 7 Confocal microscopy of sonicated FF in droplets through *z* slices. Confocal microscopy images of a droplet containing FF sonicated for 0, 10 or 60 s before encapsulation, shown as a progression through the *z* planes of the droplet.

Supplementary Video 8 Particles diffusing freely in bulk FF networks. Confocal microscopy time lapse of beads (1 μm, green microspheres, carboxylic acid functionalized) mixed with peptide filaments. The duration of the video is 100 s.

Supplementary Video 9 Particle dynamics within an FF droplet. Confocal microscopy time lapse of beads (1 μm, green microspheres, carboxylic acid functionalized) in FF droplets. The duration of the video is 100 s.

Supplementary Video 10 Particle dynamics within an A′ droplet. Confocal microscopy time lapse of beads (1 μm, green microspheres, carboxylic acid functionalized) in A′ droplets. The duration of the video is 100 s.

Supplementary Video 11 Particle dynamics within an A_m_-A′_m_ droplet. Confocal microscopy time lapse of beads (1 μm, green microspheres, carboxylic acid functionalized) in A_m_-A′_m_ droplets. The duration of the video is 100 s.

Supplementary Video 12 Filament dynamics in heated peptide and peptide–DNA droplets. Confocal microscopy time lapses of droplets containing FF, A′ and A_m_-A′_m_ during heating to 50 °C over time. The videos for the FF, A′ and A_m_-A′_m_ droplets last for 160, 180 and 180 min, respectively.

Supplementary Video 13 Heating droplets containing FF. Confocal microscopy time lapses of droplets containing FF during heating to 50 °C over time, with insets showing regions of interest. The duration of the video is 160 min.

Supplementary Video 14 Heating droplets containing A′. Confocal microscopy time lapses of droplets containing A′ during heating to 50 °C over time, with insets showing regions of interest. The duration of the video is 180 min.

Supplementary Video 15 Heating droplets containing A_m_-A′_m_. Confocal microscopy time lapses of droplets containing A_m_-A′_m_ during heating to 50 °C over time, with insets showing regions of interest. The duration of the video is 180 min.

### Source data

Source Data Figs. 2–6 and Extended Data Figs. 1–4 Statistical source data.

## Data Availability

The data in this study are available in the paper and supporting files. Additional formats of the data are available from the corresponding author upon request. Source data are provided with this paper.
