# Supplementary material for: Designer peptide–DNA cytoskeletons regulate the function of synthetic cells
Source: Nat Chem. 2024 Apr 23;16(8):1229–39. doi: 10.1038/s41557-024-01509-w (PMC11322001; doi:10.1038/s41557-024-01509-w)
Supplement: Supplementary file 1 — Supplementary Discussions 1 and 2, Figs. 1–45 and Tables 1 and 2. [file 41557_2024_1509_MOESM1_ESM.pdf]

nature chemistry

Article

https://doi.org/10.1038/s41557-024-01509-w

Designer peptide–DNA cytoskeletons regulate the function of synthetic cells

In the format provided by the authors and unedited

Table of Contents

Supplementary Figure 1. Synthesis of azide-modified peptide
Supplementary Figure 2. Peptide-DNA synthesis scheme
Supplementary Figure 3. Purification and characterization of the peptide-DNA library
Supplementary Figure 4. Bending stiffness measurement of FmocFF fibers prepared by the DMSO switch method
Supplementary Table 1. DNA sequences
Supplementary Figure 5. Library of peptide-DNA crosslinkers
Supplementary Figure 6. DNA duplex melting temperatures
Supplementary Table 2. DNA-crosslinker parameters
Supplementary Discussion 1. Peptide-DNA Material Design
Supplementary Figure 7. Wide-field views of confocal images
Supplementary Figure 8. Aspect ratios and widths of all bundles in samples
Supplementary Figure 9. Scheme describing tactoid-like shape of the assembled structures
Supplementary Figure 10. Intensity line scans from confocal microscopy images of large bundles
Supplementary Figure 11. Widths of structures at varying DNA-crosslinker concentrations
Supplementary Figure 12. Mesh sizes of A-A' networks at varying DNA-crosslinker concentration
Supplementary Figure 13. Peptide/peptide-DNA assemblies of A_m-A'_m
Supplementary Figure 14. Fraction of peptide-DNA structures above 1 μm with various crosslinkers
Supplementary Figure 15. Comparing length between peptide and peptide-DNA structures in DMSO/water and water
Supplementary Figure 16. Peptide-DNA bundles in water
Supplementary Figure 17. Peptide fibers without DNA crosslinkers stained with acridine orange
Supplementary Figure 18. Width, percent coverage and fraction of fiber alignment within bundles
Supplementary Discussion 2. Peptide-DNA bundle formation in water: crosslinker density, filament alignment
Supplementary Figure 19. Scheme for assembling spindles or bundles using solvent conditions
Supplementary Figure 20. Confocal microscopy of sonicated bundles from A-A' in water
Supplementary Figure 21. Spindle assembly guided by DNA hybridization
Supplementary Figure 22. Complex shear moduli of peptide and peptide-DNA materials
Supplementary Figure 23. Strain sweeps for peptide-DNA gels
Supplementary Figure 24. Schematic of thin-tactoid networks
Supplementary Figure 25. Mesh size quantification
Supplementary Figure 26. Shear modulus of A' during heat-cool cycles
Supplementary Figure 27. Loss moduli from heat-cool cycles of peptide-DNA
Supplementary Figure 28. Analysis of peptide-DNA mechanics during heating from 25 to 50°C
Supplementary Figure 29. Wide-field confocal images of water-in-oil droplets containing FF cortex and free FITC
Supplementary Figure 30. Confocal images of water-in-oil droplets including peptide-DNA
Supplementary Figure 31. Length distributions of peptide-DNA materials within droplets
Supplementary Figure 32. Scheme of shape deformation analysis, D_{in}/D_{out}
Supplementary Figure 33. Varying length of peptide filaments within droplets via sonication
Supplementary Figure 34. Probability distribution of structures across the radius of droplets smaller than ~ 40 μm
Supplementary Figure 35. Droplets containing FF and A-FITC
Supplementary Figure 36. Photobleaching correction for DNA-triggered release of payload
Supplementary Figure 37. MSDs of probe particles inside droplets with FF, A' and A_m-A'_m
Supplementary Figure 38. Illustrations of the lipid-droplet fabrication methods
Supplementary Figure 39. Area, circularity and solidity of one-step lipid-droplets
Supplementary Figure 40. Percentage of one-step lipid-droplets containing structures or deformations
Supplementary Figure 41. Lipid-coated two-step FF droplets
Supplementary Figure 42. Confocal microscopy of heated lipid-coated droplets
Supplementary Figure 43. Correlation analysis along the contour of droplets
Supplementary Figure 44. The effect of heat on filament distributions at the oil-water boundaries of large spherical lipid droplets with peptide-DNA
Supplementary Figure 45. Local curvature analysis of peptide-DNA lipid-droplets (two-step fabrication) before and after 90 min heating at 50°C

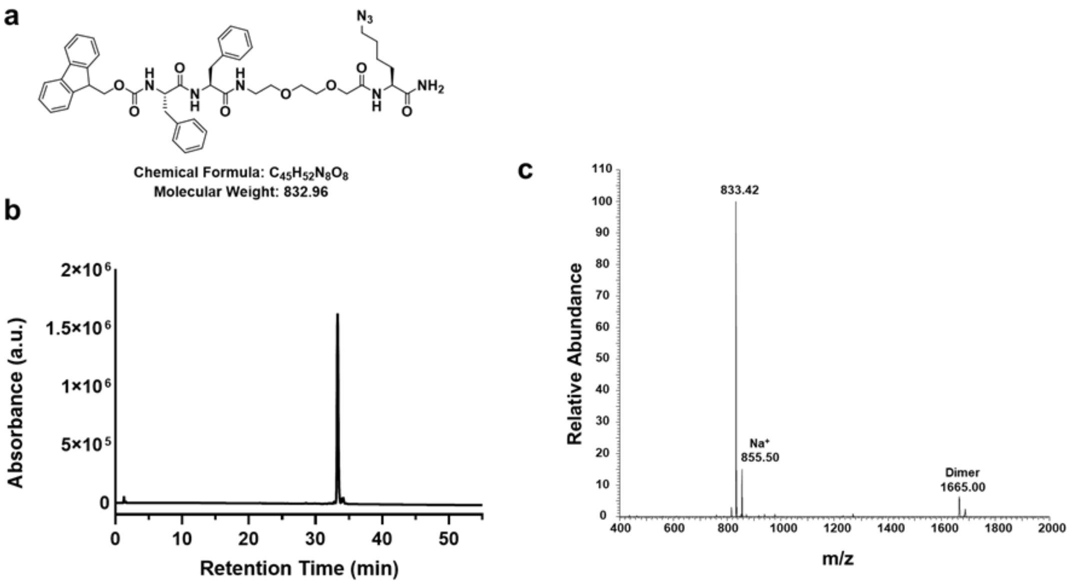

Supplementary Figure 1. *Structure and characterization of an azide-modified Fmoc-FF-PEG$_2$ peptide.* (A) Chemical structure and expected mass of peptide. (B) Analytical HPLC (monitoring peptide absorbance at 214 nm) and (C) ESI-MS confirmed the peptide identity and high purity.

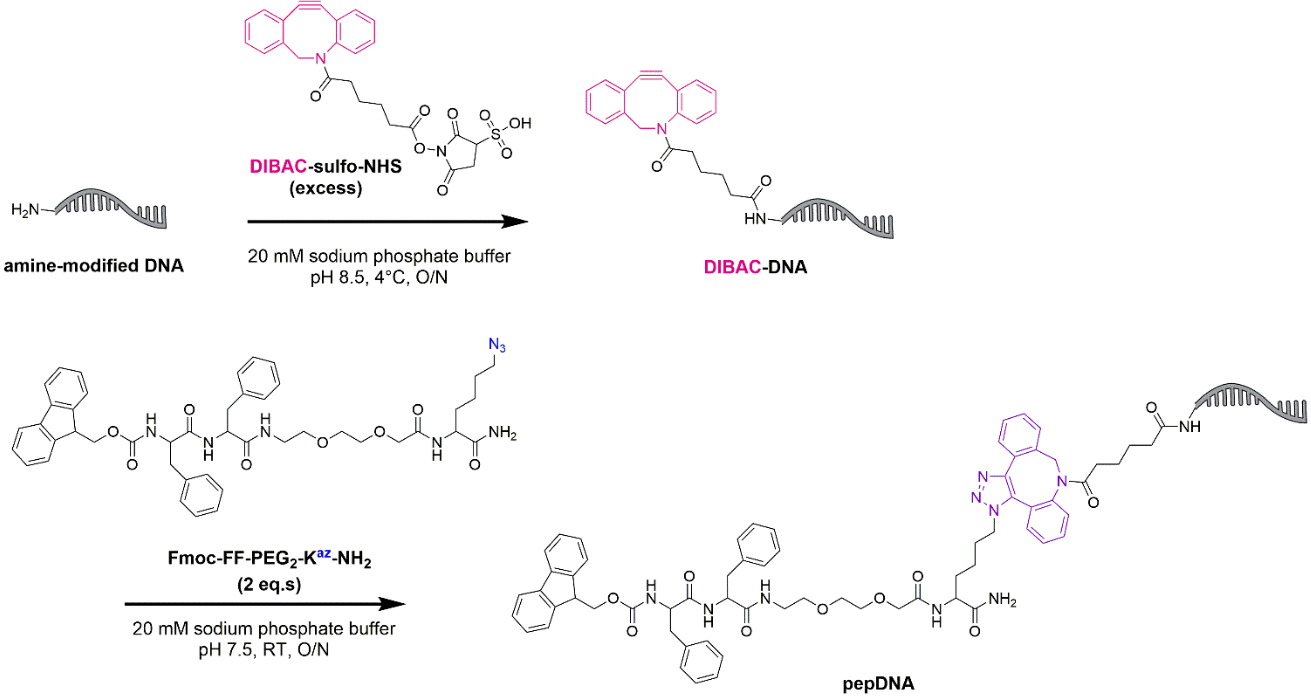

Supplementary Figure 2. Peptide-DNA synthesis scheme.

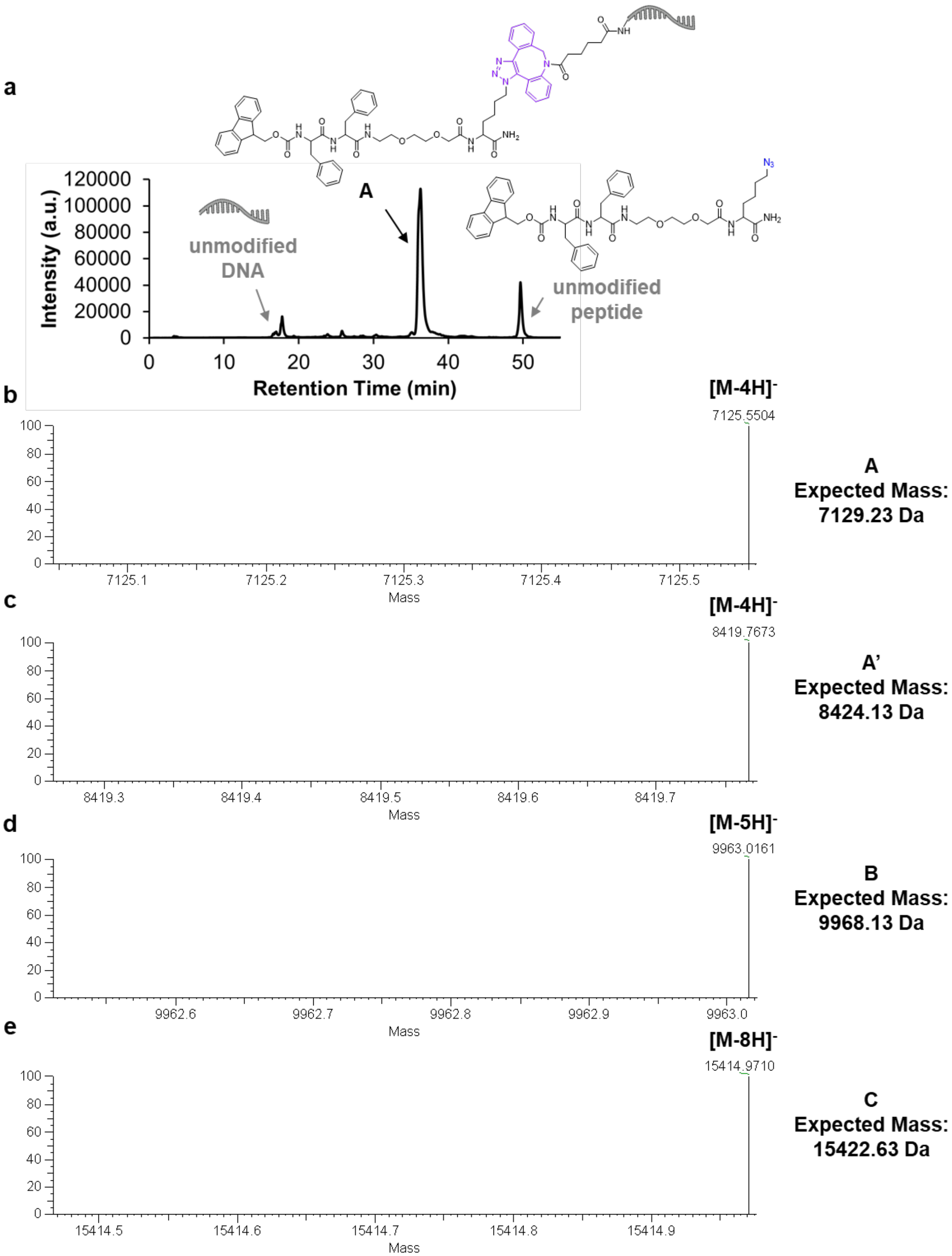

a
120000
100000
80000
60000
40000
20000
0
Intensity (a.u.)
unmodified DNA
A
unmodified peptide
0
10
20
30
40
50
Retention Time (min)
b
[M-4H]-
7125.5504
7125.1
7125.2
7125.3
7125.4
7125.5
Mass
A
Expected Mass:
7129.23 Da
c
[M-4H]-
8419.7673
8419.3
8419.4
8419.5
8419.6
8419.7
Mass
A'
Expected Mass:
8424.13 Da
d
[M-5H]-
9963.0161
9962.6
9962.7
9962.8
9962.9
9963.0
Mass
B
Expected Mass:
9968.13 Da
e
[M-8H]-
15414.9710
15414.5
15414.6
15414.7
15414.8
15414.9
Mass
C
Expected Mass:
15422.63 Da

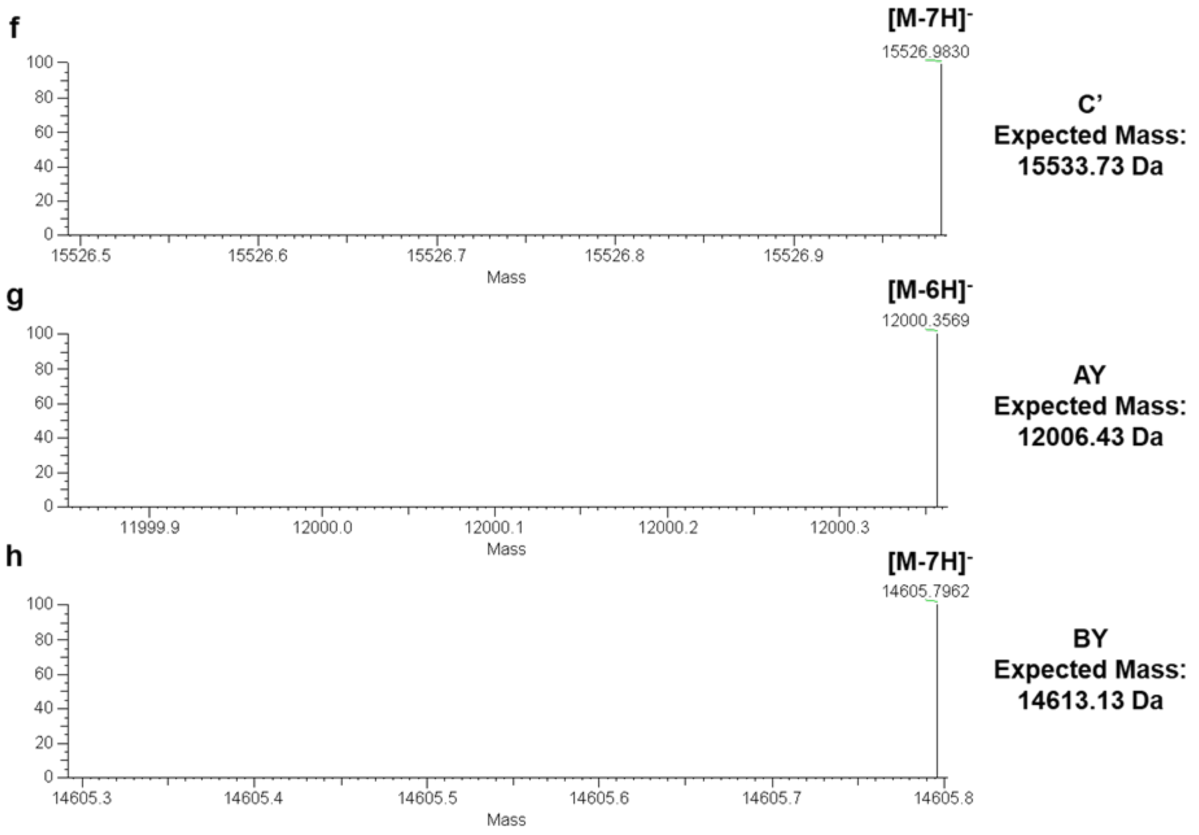

Supplementary Figure 3. *Purification and characterization of the peptide-DNA library.* (a) HPLC spectra of the reaction mixture (monitoring DNA absorbance at 260 nm). Unreacted DNA elutes at 19 min, the peptide-DNA product elutes at 32-35 min, and the unreacted aromatic peptide elutes at 50 min. The peptide-DNAs were then isolated as highly pure materials, with no unmodified DNA or free peptide. (b-h) The identity of the peptide-DNA was confirmed using deconvoluted spectra (no threshold limit applied) from high- resolution ESI-MS (Thermo Scientific Q Exactive HF-X).

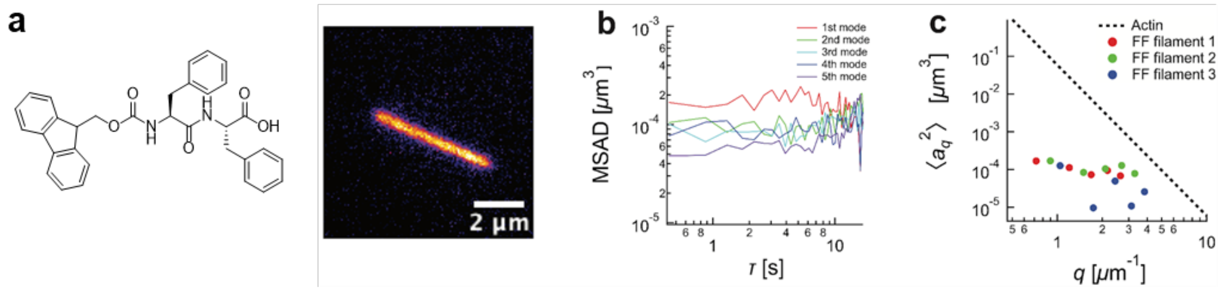

Supplementary Figure 4. *Bending stiffness measurement of FmocFF fibers prepared by the DMSO switch method.* (a) Chemical structure and a confocal microscopy image of assembled fibers (Fmoc-FF-OH). Fibers were stained with ThT in 50 wt% sucrose solution for analysis (the corresponding movie is shown in Movie S1.) (b) The mean-square amplitude differences (MSAD) of the first five bending modes of a 5 µm fiber are plotted as a function of lag time τ: $\langle \Delta a_q(\tau)^2 \rangle = 1/2 \langle \{a_q(t+\tau) - a_q(t)\}^2 \rangle$(See details in the Materials and Methods section). At long lag times, the shape of fibers will be uncorrelated with their initial shapes, resulting in $\langle \Delta a_q(\tau \rightarrow \infty)^2 \rangle = \langle a_q(0)^2 \rangle$. Figure (b) demonstrates that the MSADs exhibit plateaus at ~ 5 s. (c) The amplitude variances $\langle a_q(0)^2 \rangle$ of three FF fibers, extracted from the plateaus of (b), are plotted as a function of the mode number q as circles. In the case of thermal equilibrium, the equipartition theorem predicts $\langle a_q(0)^2 \rangle = 1/(l_p q^4)$ with the thermal persistence length $l_p = \kappa_B / k_B T$, where k_B and T are defined as the Boltzmann constant and temperature, respectively. For comparison, we plotted the expected amplitude variations for actin filaments as the dotted line (l_p~17 µm).[1] We found that the amplitude variance of FF fibers was independent of the wavenumber q and greatly suppressed compared to actin filaments. These findings suggest that the bending stiffness of FF fibers is larger than that of actin filaments. Consequently, a noise floor dominates the thermal bends, resulting in q-independent amplitude variance as previously reported.[2,3]

Supplementary Table 1. DNA Sequences

Name	Sequence (5' to 3')	# bases	GC Content (%)
**A-NH_2**	CTC AGT GGA CAG CCT TTT T– NH_2	19	47.4
**A' -NH_2**	GGC TGT CCA CTG AGA GTC ATG AC– NH_2	23	56.5
**NH_2-B**	NH_2 – TTT TTG TTC TGG AGC GTT GGA CGA AAC T	28	42.9
**NH_2-C**	NH_2 – CA GTA CAG TTT CGT CCA ACG CTC CAG AAC TGA GGC TGT CCA CTG AG	46	54.3
**NH_2-C'**	NH_2 – CTC AGT GGA CAG CCT CAG TTC TGG AGC GTT GGA CGA AAC TGT ACT G	46	54.3
**NH_2-AY**	NH_2 – TTT TTC TCA GTG GAC AGC CTT TTC ATG CGG ATC CA	35	45.7
**NH_2-BY**	NH_2 – TTT TTT GGA TCC GCA TGA TTG TTC TGG AGC GTT GGA CGA AAC T	43	44.2
**NH_2-A_m**	NH_2 – AAA AAA AAA AAA AAA AAA AAA AAA A	25	0
**NH_2-A'_m**	NH_2 – TTT TTT TTT TTT TTT TTT TTT TTT T	25	0
**A-I**	AAA AAG GCT GTC CAC TGA G	19	47.4

*AY and BY adapted from[4]

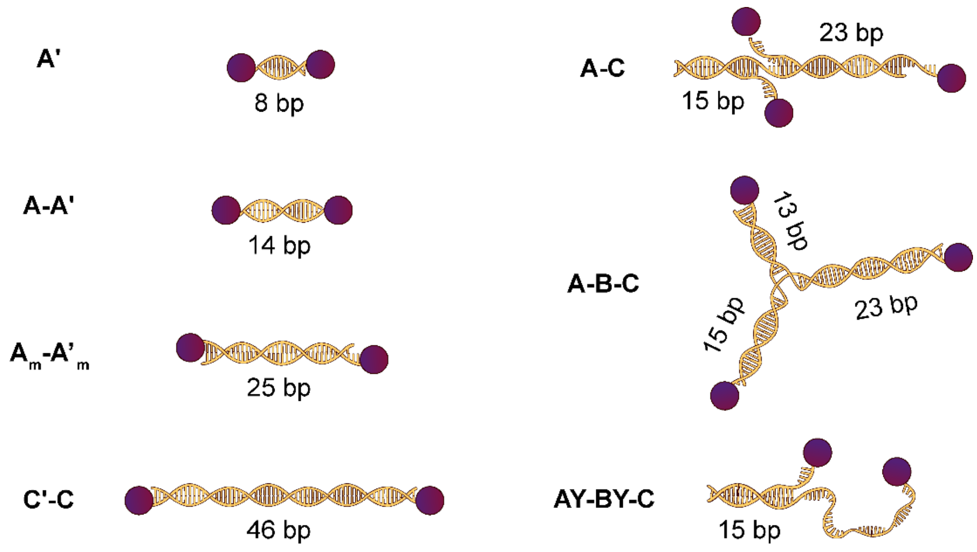

Supplementary Figure 5. *Library of peptide-DNA crosslinkers.* Schemes of peptide-DNA crosslinkers, notated with the length in base-pairs (bp) of the double-stranded DNA regions.

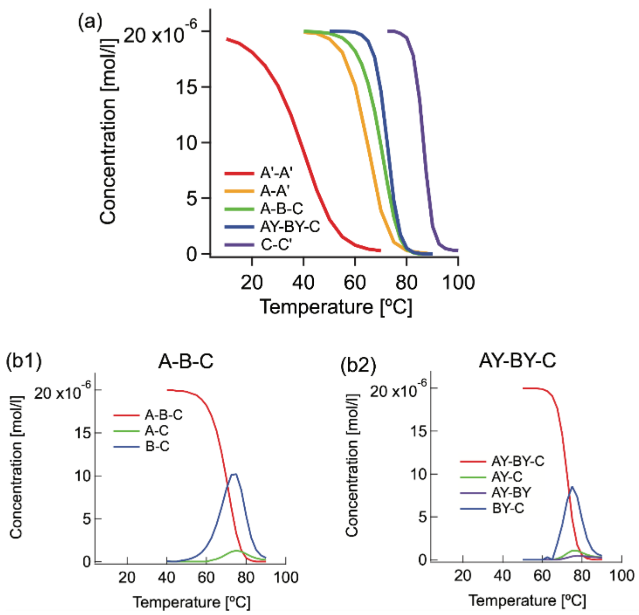

Supplementary Figure 6. *DNA duplex melting temperatures.* (a) Concentration of hybridized DNA constructs at various temperatures during a heat ramp of A'-A', A-A', A-B-C, AY-BY-C, and C'-C simulated by NuPack 4.0.[5] (b) Concentrations of partial duplexes forming within mixtures of three strands: (b1) A-C, B-C and A-B-C, and (b2) AY-BY, AY-C, BY-C, and AY-BY-C. We input 20 µM of each DNA strand in 150 mM Na^+. Melting temperatures (T_m) were taken from the inflection points of the decreasing sigmoidal curves and summarized in **Supplementary Table 2**.

Supplementary Table 2. DNA-crosslinker parameters.

DNA-crosslinker	Length (bps)	Tm (°C)	Length between peptides (nm)	Valency	Relative flexibility	Average aspect ratio
A'	8	40	3	2	Rigid	55.5
A-A'	14	64	5	2	Rigid	21.7
A-B-C	38	74	2, 8	3	Flexible	26.4
AY-BY-C	41	78	8, 11, 11	3	Rigid	14.8
A_m-A'_m	25	65	9	2	Rigid/ flexible	4.9
C'-C	46	87	16	2	rigid	n/a
A-C	15	68	5	2	flexible	n/a

*Average aspect ratios of all bundles, prepared in DMSO/water

---

Supplementary Discussion 1. *Peptide-DNA Material Design*

DNA crosslinkers: DNA crosslinks offer tunability and programmability. Generally, a larger number of base-pairs enhances the hybridization energy of the construct. The melting temperatures for the hybridized DNA constructs used in this manuscript span from 40 (A') – 78°C (AY-BY-C), which corresponds to a wide range of crosslinker hybridization strengths (**Supplementary Table 2**). We also explored a random sequence (A-A') versus a polyT-polyA sequence (A_m-A'_m) with a similar melting temperature (but a longer duplex). This design allows us to study if the sequence composition plays a role for forming bundled structures. We also explored multivalent crosslinkers, extending beyond the simple DNA duplex design. One DNA construct is a 'classic' Y-shaped DNA (AY-BY-C), which has been shown to hybridize into a rigid, planar Y junction.[4] The other trivalent DNA crosslinker (A-B-C) can adopt configurations other than planar due to single-stranded DNA overhangs, and is therefore expected to form more flexible junctions. While both trivalent crosslinkers have very similar melting temperatures (A-B-C: 74°C, AY-BY-C: 78°C), they have different geometries that afford rigid or flexible crosslinker properties.

Peptide: The following properties of peptides are useful for cytoskeletal mimetic materials.

1. **Filaments (dimensions):** Self-assembled peptide filaments can be as thin (~10 nm width) and as long (up to ~10 microns) as cytoskeletal filaments, within aqueous environments.
2. **Supramolecular:** Actin filaments can polymerize and depolymerize, actively changing lengths. The transient binding of synthetic supramolecular fibers allows for artificial recreation of the dynamics of cytoskeletal filaments.

3. **Simple (dipeptide):** Small molecules are easier to synthesize and process compared to reconstituted full proteins.

Concentrations, Solvents, Salts, and Preparation:

Peptide concentration: The Fmoc-FF dipeptide can form gels with similar mechanical properties to actin, at low weight percentages.

Physiological pH and salt: The assemblies in this study were prepared at physiological pH (7.5) and salt (150 mM NaCl) conditions to demonstrate their utility in biological contexts.

DMSO concentration: This volume percent of DMSO is within the acceptable range (below 20%) to use for biological applications. It is also the appropriate amount for assembling diphenylalanine gels with storage moduli at ~10 Pa, similar to actin networks.

Annealing: Annealing provides optimized conditions for promoting DNA hybridization.

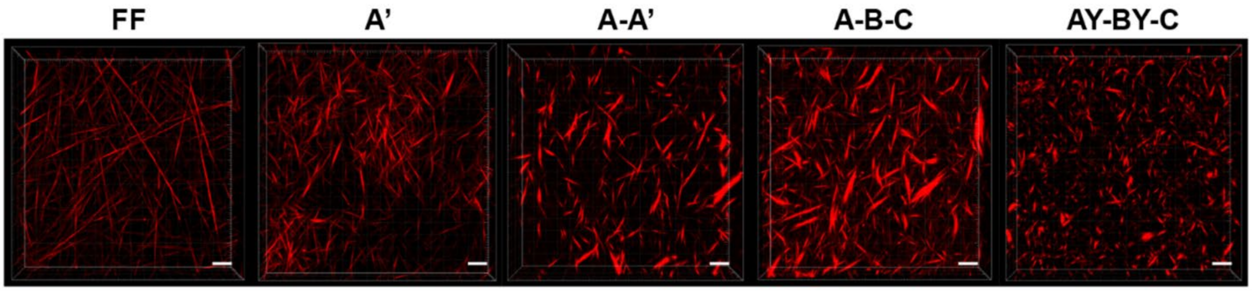

Supplementary Figure 7. Wide-field 3D confocal images of DNA-crosslinked peptide gels stained with Nile Red. Scale bars: 30 μm.

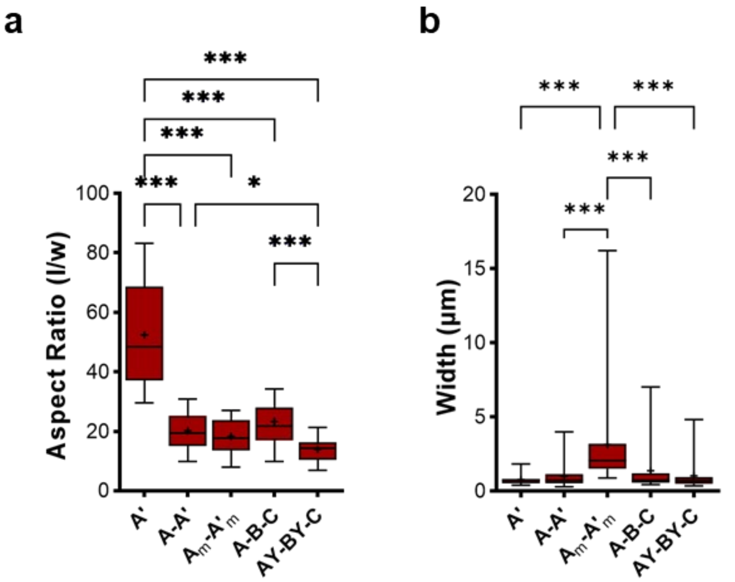

Supplementary Figure 8. *Aspect ratios and widths of bundles formed with peptide-DNA crosslinkers.* Box-and-whisker plots of aspect ratio (a) and width (b) of peptide-DNA assemblies from maximum intensity confocal images (*n*=34 structures for A', *n*=53 for A-A', *n*=47 for A_m-A'_m, *n*=43 for A-B-C, *n*=54 for AY-BY-C). Center line is median, cross is the mean, the box extends to the 25th and 75th percentiles, and whiskers extend to the minimum and maximum values. Statistics were performed using a Kruskal-Wallis test with a Dunn's multiple comparisons test (**** $p = 2.202 \times 10^{-9}$ for A' vs. A-A', 1.083×10^{-11} for A' vs. A_m-A'_m, 7.981×10^{-6} for A' vs. A-B-C, $<1 \times 10^{-15}$ for A' vs. AY-BY-C, 2.074×10^{-5} for AY-BY-C vs. A-B-C; ** $p = 0.00249$ for A-A' vs. AY-BY-C; non-significant $p = 0.122$ for A_m-A'_m vs. AY-BY-C, 0.259 for A_m-A'_m vs. A-B-C, >1.00 for A-A' vs A_m-A'_m, >1.00 for A-A' vs. A-B-C.

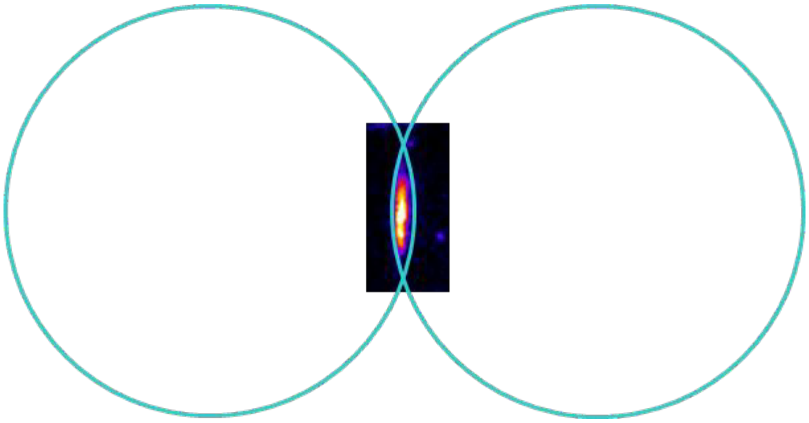

Supplementary Figure 9. *Scheme describing tactoid-like shape of the assembled structures in Fig. 2.* Tactoid shape depicted by the overlap region of two circles, overlaid onto an AY-BY-C structure.

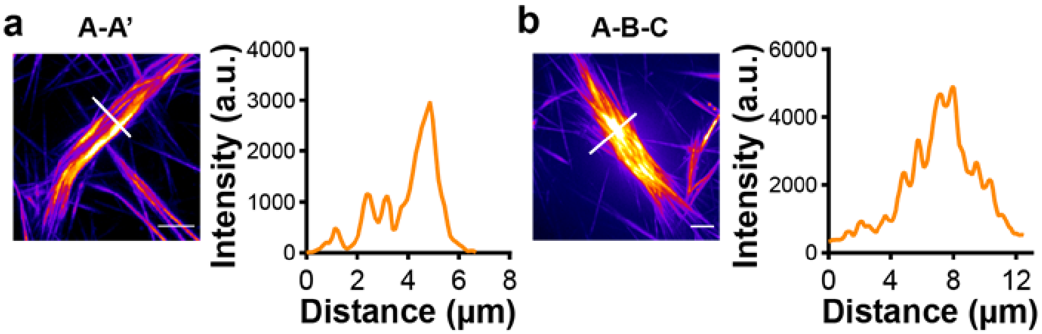

Supplementary Figure 10. Intensity line scans from confocal microscopy images of (a) A-A' and (b) A- B-C large-bundles, stained with Nile Red. Scale bars: 5 µm.

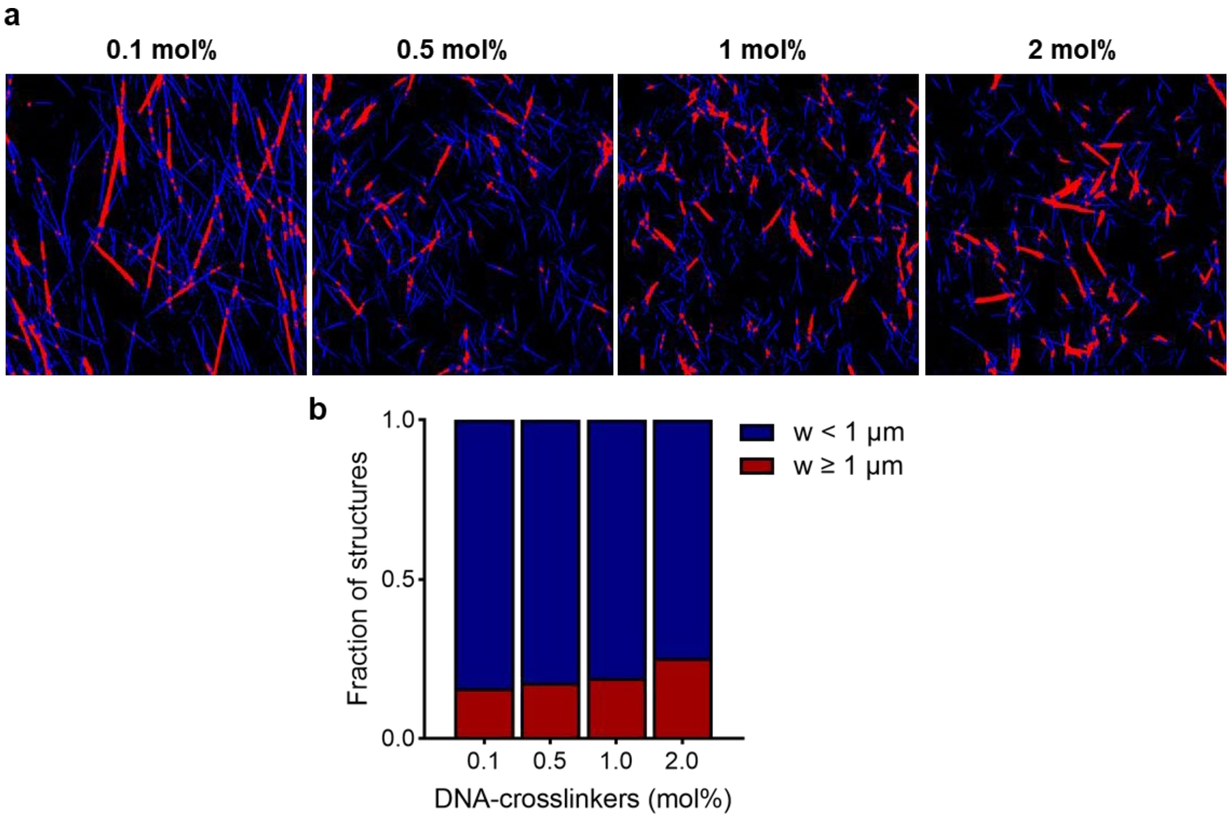

Supplementary Figure 11. *Widths of structures at varying DNA-crosslinker concentrations.* (a) Images from **Extended Fig. 1a** were processed using the Local Thickness ImageJ plugin to quantify and classify structures by width (from binarized confocal microscopy images) into two categories: those with a width below 1 µm (blue) and those with a width equal to or above 1 µm (red). (b) Fraction of structures with widths equal to or above (red) or below (blue) 1 µm. The number of each structure is counted based on panel (a) through visual inspection. The number of structures analyzed for each sample is n = 259, 324, 397 and 269, respectively.

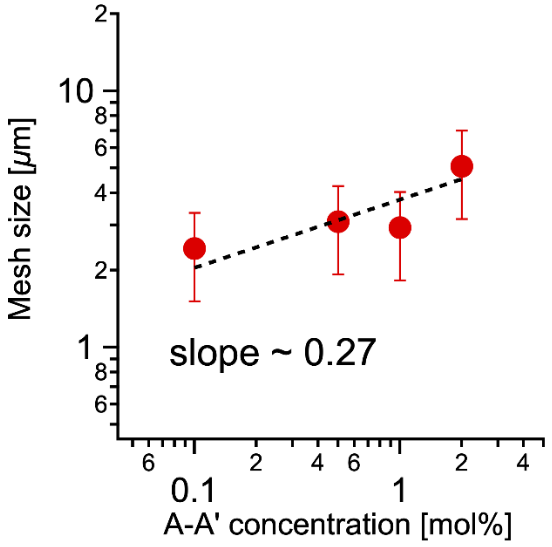

Supplementary Figure 12. *Mesh sizes of A-A' networks plotted against the crosslinker concentration.* A black dotted line is used as a visual representation of the power-law function with a slope of ~ -0.27. To measure the mesh size of the peptide networks, we binarized maximum z- projections of 3D images (40 μm in z). Histograms of the distance between fibers along *x* axis were fitted with an exponential function $P(l) = P_0\exp(-\, l/\xi)$, where *P*0 and ξ denote as the normalization constant and the mesh size, respectively.[6] From each image, we evaluate over 10,000 distances between fibers along the x-axis to determine one mesh size for each image. A minimum of *n*=3 images/locations were analyzed for each crosslinker to obtain their average mesh size and standard deviations.

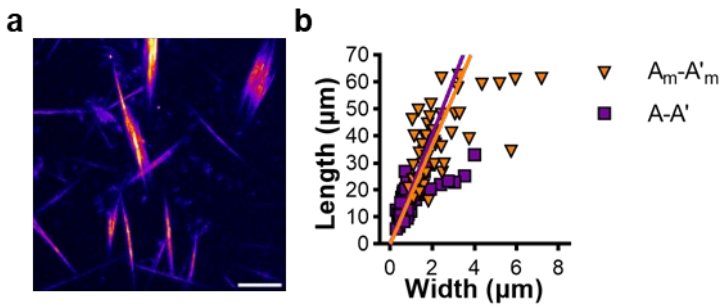

Supplementary Figure 13. *Assemblies with the DNA-crosslinker A_m-A'_m.* (a) Maximum intensity projection of z-stacked confocal image of A_m-A'_m. Scale bar: 20 μm. (b) Lengths of all bundles in A_m-A'_m (orange) compared to A-A' (purple, also shown in **Fig. 2**). A weighted linear regression (1/y^2 was applied (solid lines), showing that bundles in A_m-A'_m trend with a similar aspect ratio to A-A' bundles.

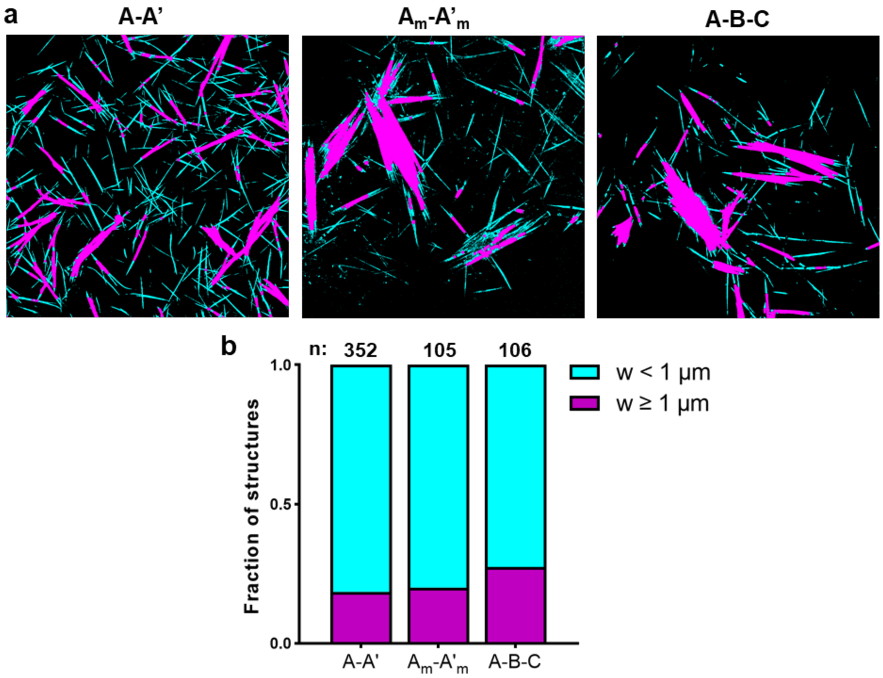

Supplementary Figure 14. *Fraction of peptide-DNA structures above 1 µm with various crosslinkers.* (a) Threshold confocal microscopy images using the Local Thickness ImageJ plugin for A-A’ (left), A_m-A'_m (middle) and A-B-C (right). Images thresholded and classified by width of structures, binned into two categories: peptide-DNA structures with width below 1 µm (cyan) and above or equal to 1 µm (magenta). (b) Fraction of structures with widths equal to or above (magenta) or below (cyan) 1 µm for A-A’ (0.185), A_m-A'_m (0.200) and A-B-C (0.274). Further information regarding the calculation methods for these fractions is available in the caption of **Supplementary Fig. 11**.

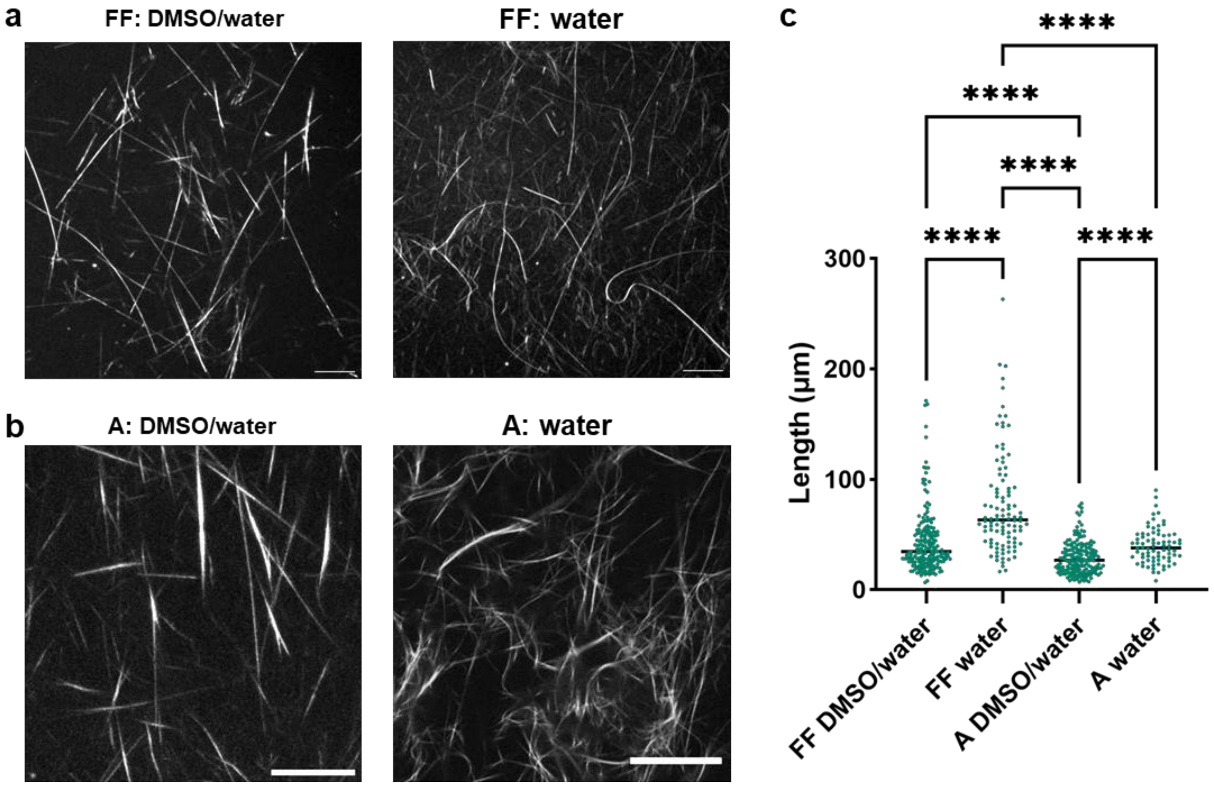

Supplementary Figure 15. *Comparing length between peptide and peptide-DNA structures in DMSO/water and water.* (a) Confocal images (maximum intensity projection) of peptide (FF) prepared in DMSO/water and water. Scale bars: 30 μm. (b) Confocal images (maximum intensity projection) of A (1 mol%) in DMSO/water and water. Scale bars: 15 μm. (c) Length of peptide structures (*n*=193 filaments for FF DMSO/water, *n*=102 filaments for FF water, *n*=167 filaments for A DMSO/water, *n*=77 filaments for FF water). Median length values are 35 μm for FF in DMSO/water, 63 μm for FF in water, 26 μm for A in DMSO/water, and 38 μm for A in water. Statistical analysis using a Kruskal-Wallis t-test and a Dunn's multiple comparisons test (**** $p = 8.717 \times 10^{-7}$ for FF DMSO/water vs. A DMSO/water, $p = 3.290 \times 10^{-13}$ for FF DMSO/water vs. FFwater, $p = 4.011 \times 10^{-5}$ for A DMSO/water vs. A water, $p = 8.588 \times 10^{-8}$ for FF water vs. A water, $p < 1 \times 10^{-15}$ for A DMSO/water vs. FF water, non-significant $p > 1.00$ for FF DMSO/water vs A water). The plot shows individual points and median (black line).

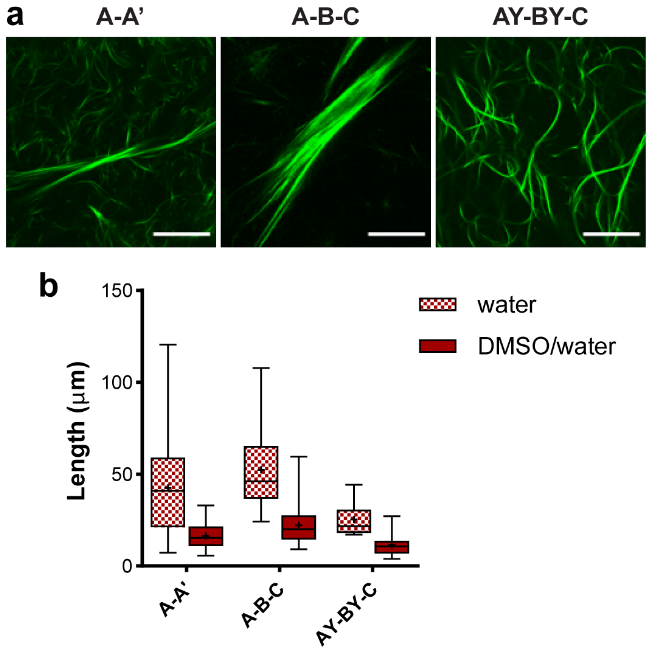

Supplementary Figure 16. *Peptide-DNA bundles in water.* (a) Confocal images of peptide-DNAs (A- A', A-B-C, AY-BY-C) assembled in water and stained with acridine orange. Scale bar: 15 µm. (b) Box-and-whisker plot of length of peptide-DNA structures in DMSO/water compared to water. Center line is median, cross is the mean, the box extends to the 25th and 75th percentiles, and whiskers extend to the minimum and maximum values. (water: *n*=19 for A-A', *n*=13 for A-B-C, *n*=9 for AY-BY-C; DMSO/water: *n*=53 for A-A', *n*=43 for A-B-C, *n*=54 for AY-BY-C).

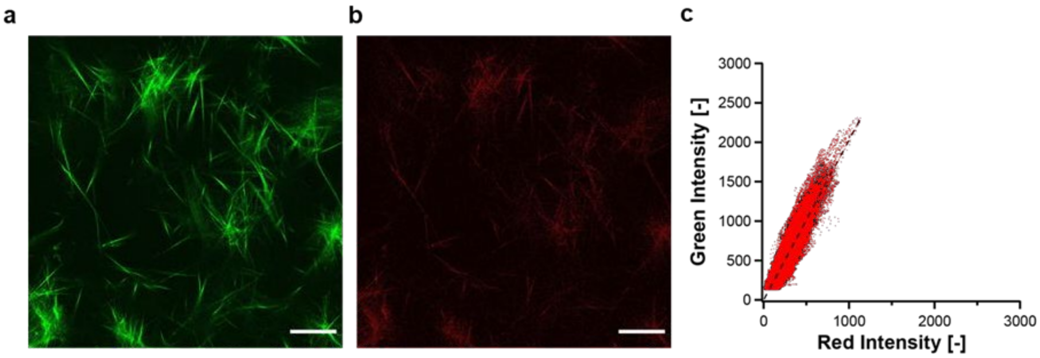

Supplementary Figure 17. *Peptide fibers without DNA crosslinkers stained with acridine orange.* (a) confocal microscopy images of green channel and (b) red channel. (c) Relation of pixel intensities between the green and red channels. Scale bars: 20 µm. In **Extended Fig. 2**, we estimate the degree of DNA hybridization localized in the bundles by measuring fluorescence intensities in the two spectral regions (green and red) of AO. Although AO is known as a nucleic acid binding dye, we found that AO also binds to Fmoc-FF and emits both green and red fluorescence. We plotted the fluorescence intensities on each pixel in both the green and red channels and found a strong correlation between them. We determined a correlation coefficient $\beta = I_{\text{green,FF}}/I_{\text{red,FF}}$ by performing a linear fit to this plot.

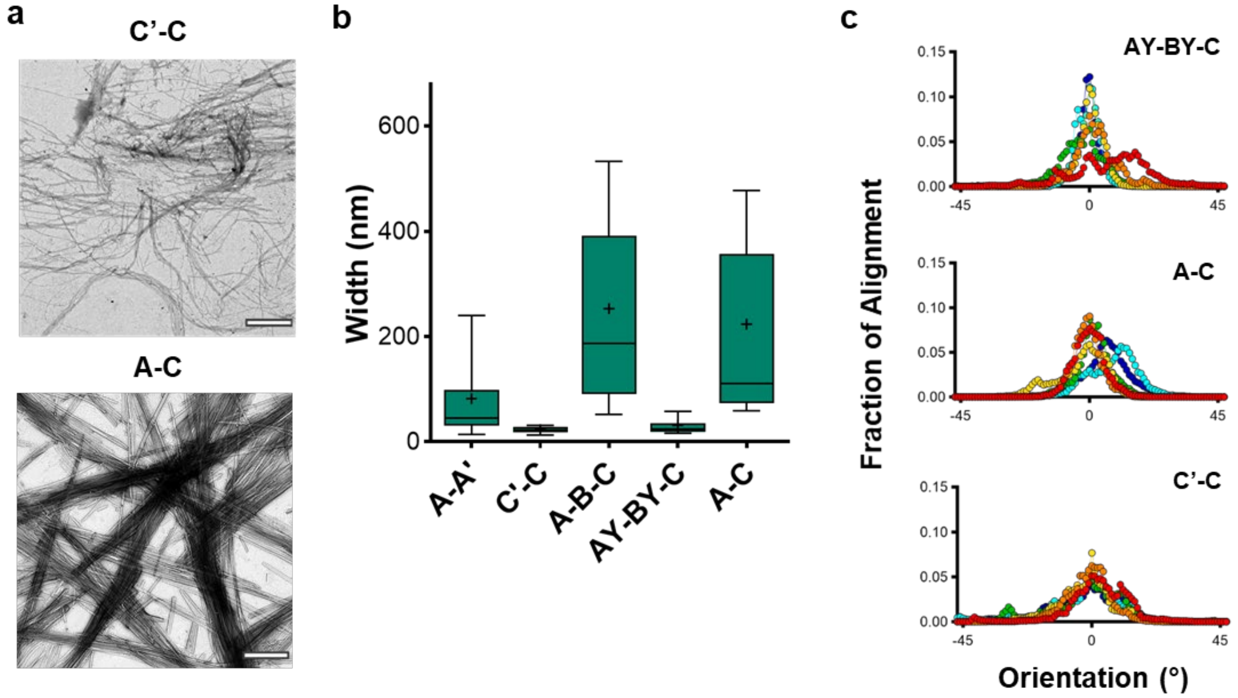

Supplementary Figure 18. *Width and fraction of fiber alignment within bundles.* (a) TEM images of C'- C and A-C. Scale bars: 1 µm. (b) Box-and-whisker plots of width of peptide-DNA assemblies from TEM images (*n*=50 structures for C'-C and A-A', *n*=49 for A-C, *n*=30 for A-B-C, *n*=99 for AY-BY-C). Center line is median, cross is the mean, the box extends to the 25th and 75th percentiles, and whiskers extend to the 10th and 90th percentiles. (c) Histograms of the relative alignment angles of fibers within bundles from TEM images (*n* = 6 bundles).

Supplementary Discussion 2. *Peptide-DNA bundle formation in water: crosslinker density and filament alignment*

As crosslinking density was shown to be important for bundle dimensions of actin,[7] we evaluate it in the various structural states of our cytoskeletal mimetics. To do this, we stain crosslinked architectures with acridine orange (AO) (**Supplementary Fig. 16**). As AO fluoresces red when bound to ssDNA and green when bound to dsDNA, it allows us to estimate the degree of DNA hybridization within bundles from fluorescence intensities in these two spectral regions (green channel: I_{green}, red channel: I_{red}) (**Extended Fig. 2a-f**). We quantify I_{green}-βI_{red} values, which correspond to the difference of fluorescence intensities of dsDNA and ssDNA (I_{dsDNA} and I_{ssDNA}), I_{green}-βI_{red}=I_{dsDNA}-βI_{ssDNA}, accounting for the background non-specific peptide-AO interactions with the average ratio between green and red channels, β=$I_{green,FF}/I_{red,FF}$ (**Supplementary Fig. 17**, see Methods and SI). Thus, high I_{dsDNA}- βI_{ssDNA} values should indicate high crosslinker density. We generate color maps and histograms of I_{dsDNA}-βI_{ssDNA} values from the confocal images (**Extended Fig. 2b**). The map of I_{dsDNA}-βI_{ssDNA} for A shows negative values (no dsDNA), in line with ssDNA-peptide fibers (**Extended Fig. 2c,f**). All DNA-crosslinked architectures show positive average values of I_{dsDNA}-βI_{ssDNA}, confirming the presence of DNA hybridization (**Extended Fig. 2f**).

We observe dsDNA-rich regions in large structures and ssDNA-rich regions in thinner filamentous structures in the image insets of A-A' (**Extended Fig. 2c,d**). To evaluate whether crosslinker density correlates with bundle width, we plot the bundle width distribution for A-A' against I_{dsDNA}-βI_{ssDNA} values from the long axis of the bundles (**Extended Fig. 2e**). Interestingly, the I_{dsDNA}-βI_{ssDNA} increases with bundle width. In addition, AY-BY-C generates structures with the highest amount of hybridization, possibly because of more double-stranded arms within the Y-shaped junction. These results show that larger bundles are enriched with dsDNA, and that crosslinker density can be tuned by the DNA junction design.

TEM shows (**Extended Fig. 2g**), that the bivalent A-A' crosslinker drives a higher degree of filament ordering within bundles than the trivalent A-B-C crosslinker (**Extended Fig. 2h**). Additionally, the A-C crosslinker, consisting of a short 15 bp-duplex (similar to A-A' 14 bp) but with a single-stranded DNA (ssDNA) overhang (31 bases), also reduces the alignment degree of filaments within bundles (**Supplementary Fig. 18**). Further, we test a bivalent crosslinker with a longer duplex (C-C': 46 bp vs. A-A': 14 bp). The longest crosslinker (C- C') yielded thin filament networks rather than bundles (**Supplementary Fig. 18**), recapitulating the behavior of actin crosslinkers, where shorter antiparallel protein crosslinkers promote tight bundle formation, while longer rigid crosslinkers procure looser filamentous networks.[1]. The most rigid, trivalent AY-BY-C crosslinker yields very thin (2-3 fibers) and long bundles (97.4 μm average) with a high degree of alignment (**Extended Fig. 2g, Supplementary Figs. 16,18**).

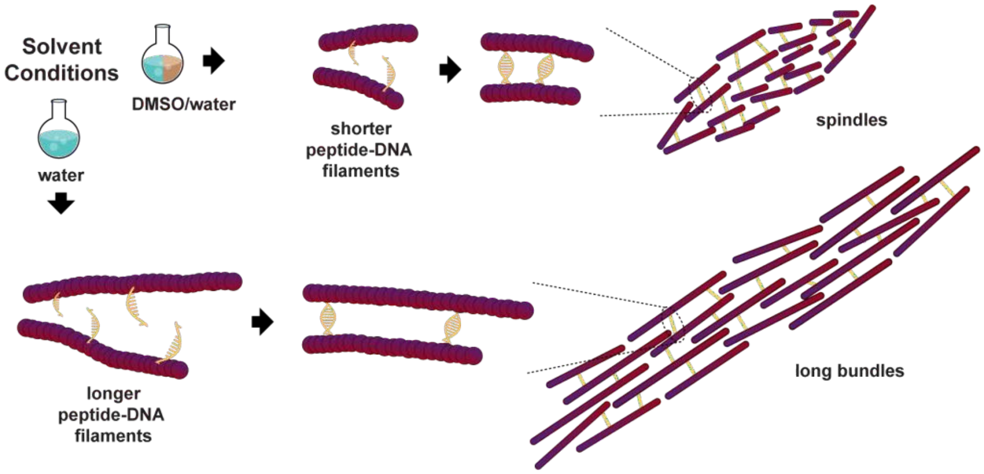

Supplementary Figure 19.. *Assembly pathway for spindles versus bundle formation.* The morphology of DNA-bundled peptides is highly dependent on filament length, which can be guided by the solvent. Long peptide-DNA filaments (water assembly) give rise to long bundles, while short filaments (DMSO/water) result in spindle-shaped bundles. Vastly different structural states can be accessed through varying filament length (through solvent as shown in **Extended Fig. 2**, or sonication shown in **Supplementary Fig. 20** below), concentration (**Extended Fig. 1**), and crosslinking (main **Fig. 2**).

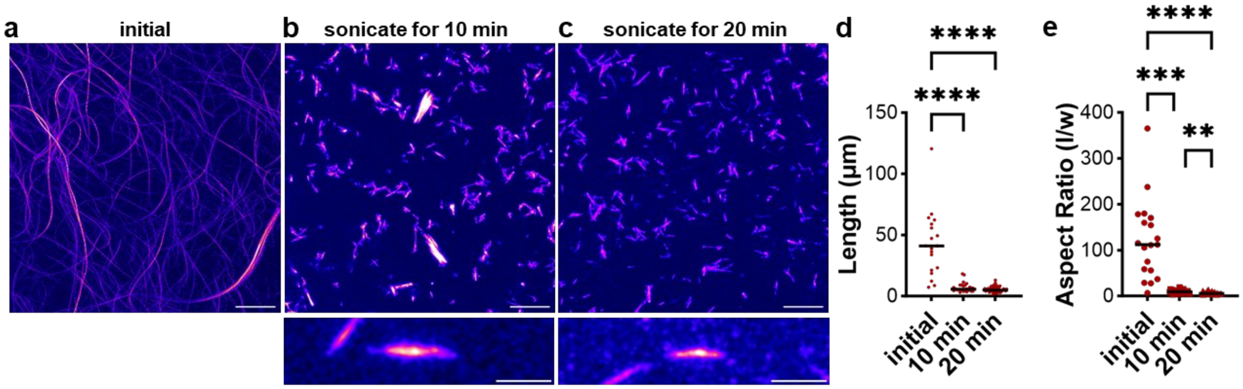

Supplementary Figure 20. *Confocal microscopy of sonicated bundles from A-A' in water.* Maximum intensity projections of confocal microscopy images of A-A' prepared by annealing in water (a), followed by bath sonication for 10 min (b) or 20 min (c). Scale bars: 20 μm. Below: insets of corresponding tactoids. Scale bars: 5 μm. All samples were stained with acridine orange. Length (d) and aspect ratio (e) quantification of structures before and after sonication for 10 or 20 min (n = 19 for initial, n = 28 for 10 min, n = 54 for 20 min). Line is the median. Statistical analysis using a Kruskal-Wallis t-test and a Dunn's multiple comparisons test ((d): **** $p = 8.155 \times 10^{-6}$ for initial vs. 10 min, $p = 7.504 \times 10^{-11}$ for initial vs. 20 min, non-significant $p = 0.293$ for 10 min vs. 20 min; (e): *** $p = 6.134 \times 10^{-4}$ for initial vs. 10 min, **** $p = 1.801 \times 10^{-12}$ for initial vs. 20 min, ** $p = 0.00135$ for 10 min vs. 20 min).

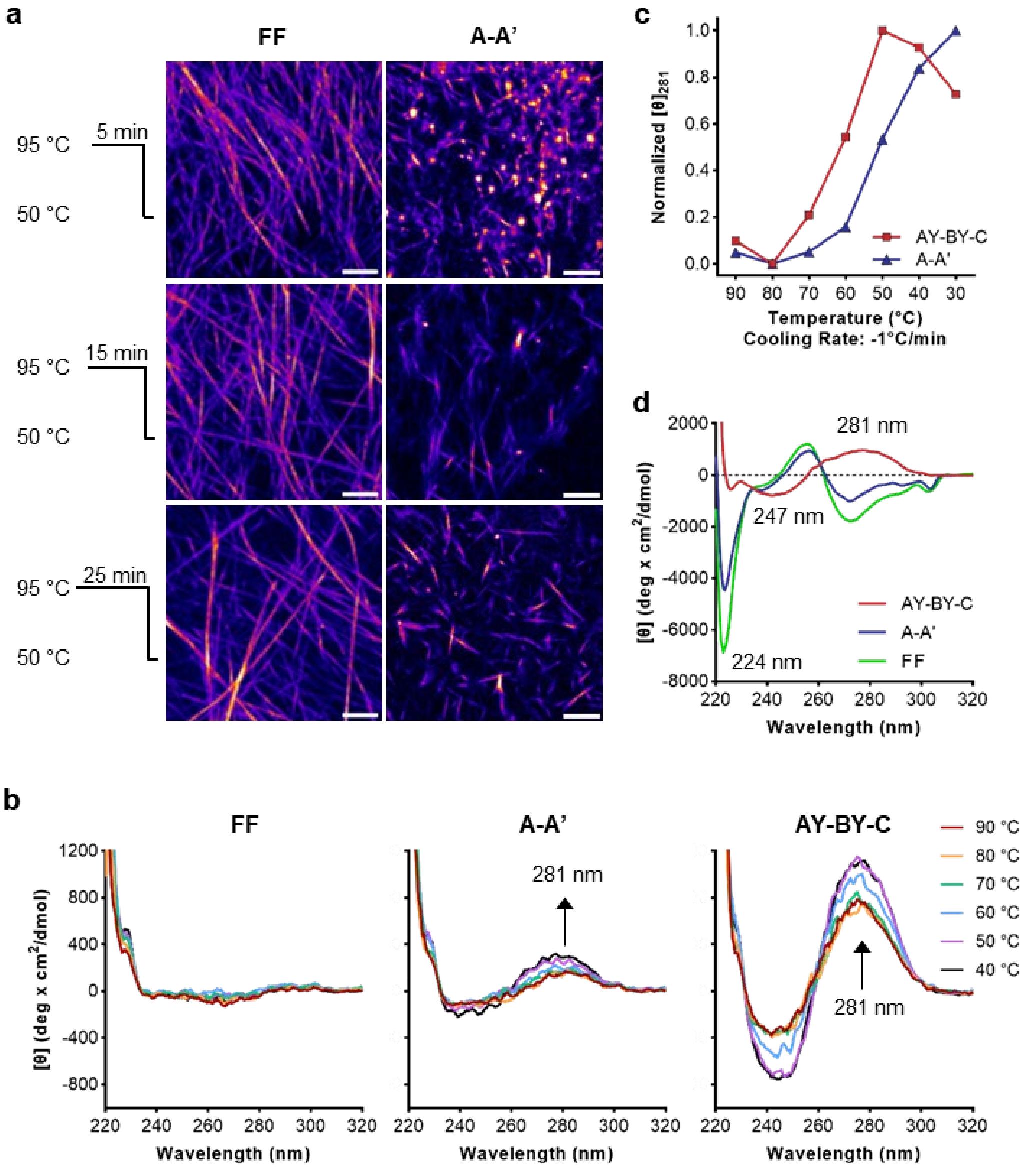

Supplementary Figure 21. *Spindle assembly guided by DNA hybridization.* (a) Maximum intensity projection confocal images of FF and A-A’ at elevated temperatures. Scale bars: 20 µm. Samples were heated to 95°C for 5 (top), 15 (middle) and 25 (bottom) minutes, followed by imaging at 50°C. (b) Circular dichroism of FF, A-A’ and AY-BY-C cooling from 90°C to 40°C. Positive CD signals at 281 nm for A-A’ and AY-BY-C increase during cooling, indicating DNA hybridization. (c) Normalized CD signals monitoring the change in the dsDNA 281 nm signal (that correspond with the respective melting temperatures) for A- A’ and AY-BY-C during cooling to room temperature. (d) CD spectra of FF, A-A’ and AY- BY-C assemblies after annealing to room temperature. All spectra show a negative signal at 224 nm, indicating β-sheet assembly. The β-sheet signal is more dominant for A-A’, while the DNA signal is stronger for AY-BY-C (negative signal at 247 nm and positive signal at 281 nm) due to the increased number of base-pairs.

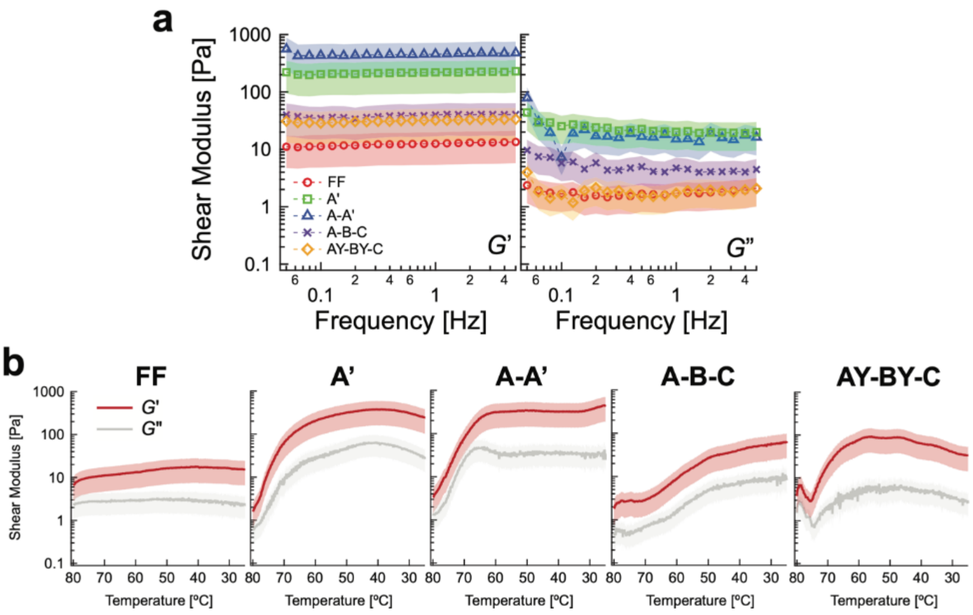

Supplementary Figure 22. The viscoelastic properties of peptide and peptide-DNA materials. Complex shear moduli are plotted (a) against various frequencies and (b) during the annealing process at 1 Hz. The error bars for (a) and (b) (reflected as the thickness of each curve) represent the estimated standard deviations based on five measurements conducted on FF gels and applied to all conditions.

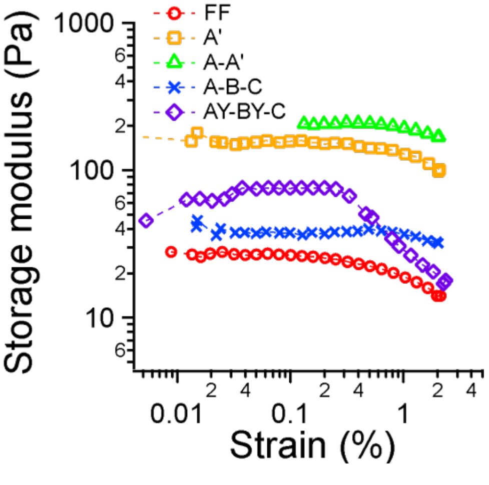

Supplementary Figure 23. *Strain sweeps for peptide-DNA gels.* To determine a linear viscoelastic regime of peptide-DNA materials, we performed strain sweep measurements on FF, A', A-A', A-B-C, and AY-BY-C. All the gels showed an almost linear viscoelastic regime up to 1.0% strain (frequency: 1.0 Hz).

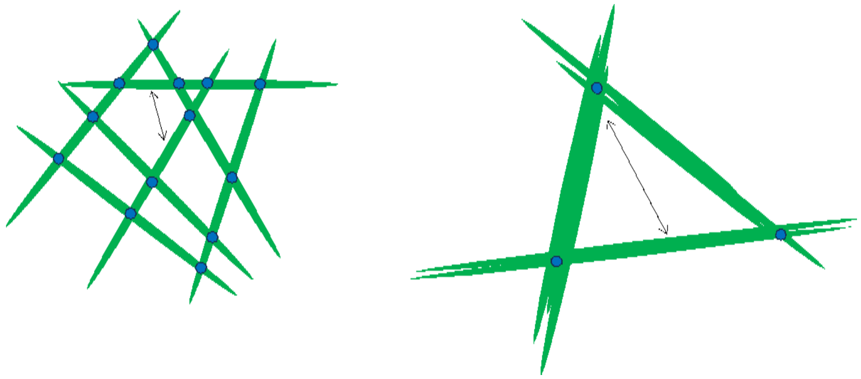

Supplementary Figure 24. *Schematic illustration of different bundled networks and their relative mesh size.* The elasticity of peptide-DNA bundled networks largely depend on the mesh size *ξ,* as suggested previously for other biopolymer networks.[8–11] In the case of bivalent DNA crosslinkers, thin-bundles are tightly crosslinked with small *ξ* as depicted in the left panel, leading to higher elasticity. Conversely, trivalent DNA crosslinkers promote formation of large-bundled networks with larger *ξ,* as depicted in the right panel, resulting in lower elasticity (**Fig. 2, 3, Supplementary Fig. 25)**.

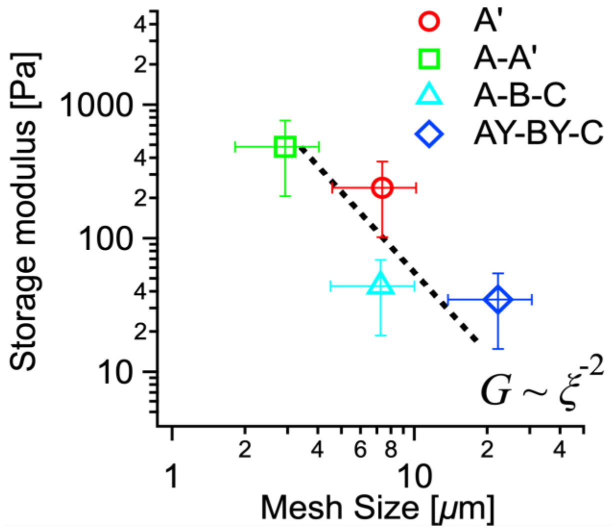

Supplementary Figure 25. *Mesh size quantification.* Storage moduli are plotted as a function of mesh size measured from confocal microscopy for A', A-A', A-B-C and AY-BY-C prepared at 1 mol% DNA-crosslinker. A black dotted line is used as a visual representation of the power-law function with a slope of ~ -2. Further information detailing the calculation method for the mesh size is available in the caption of **Supplementary Fig. 12**. From each image, we evaluate over 10,000 distances between fibers along the x-axis to determine one mesh size for each image. A minimum of n=4 images/locations were analyzed for each crosslinker to obtain their average mesh size and standard deviations. Based on these results, we can draw a quick comparison between peptide-DNA networks and actin bundle networks. Previous studies on actin bundle networks[8] have suggested that the elasticity of bundle networks can be described by the equation $G_0 \sim \kappa^2/(k_B T \xi^2 l_c^3)$, where k_B, T, and ξ represent the Boltzmann constant, temperature, and mesh size, respectively. According to a previous study on actin bundle networks crosslinked with scruin[9], the elastic modulus of these networks falls within the range of 10 to 100 Pa, with a mesh size ranging from 1 to 10 µm. These values are comparable to the findings of our study. Given that the bending stiffness κ in our system is greater than that of actin bundles (**Supplementary Fig. 4**), we can assume that the distance between crosslinkers, l_c, in the peptide-DNA networks is also larger than that in actin networks.

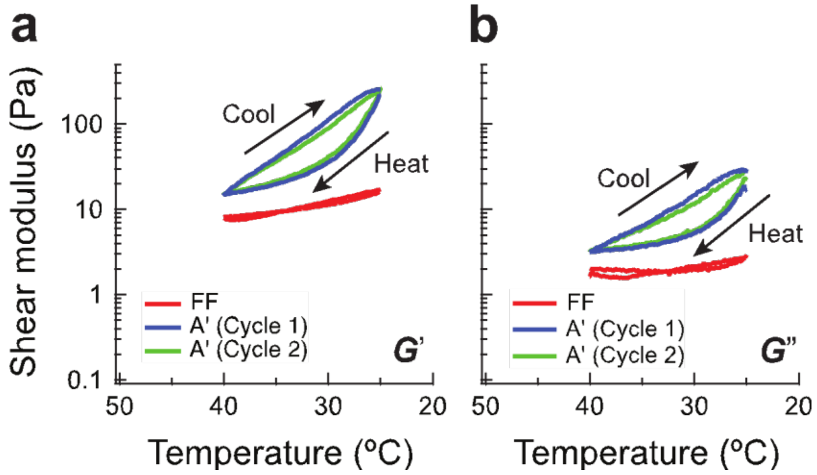

Supplementary Figure 26. *Shear modulus of FF and A' during heat-cool cycles.* Heat-cool cycles for FF and A' (25°C - 40°C - 25°C). Storage modulus (a) and loss modulus (b) are shown. FF shows minimal reversible mechanics at these temperatures, while DNA crosslinked networks are highly reversible.

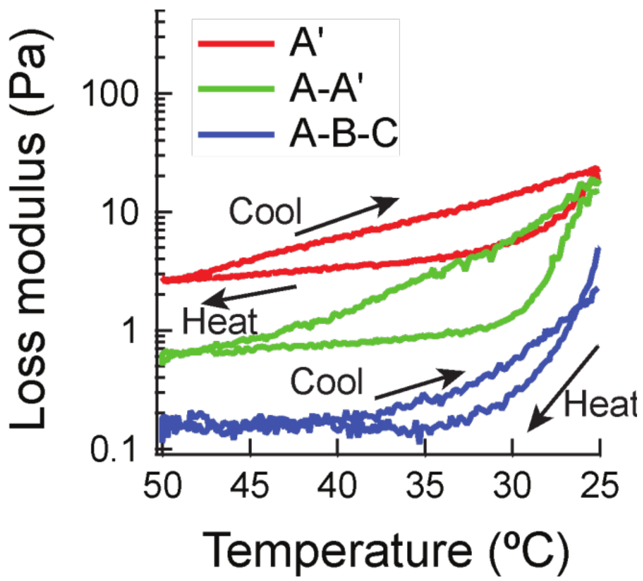

Supplementary Figure 27. *Loss moduli from heat-cool cycles of peptide-DNA.* Heat-cool cycles for peptide-DNA materials (25°C - 50°C - 25°C). The storage moduli from these heat-cool cycles are shown in **Fig. 3d**.

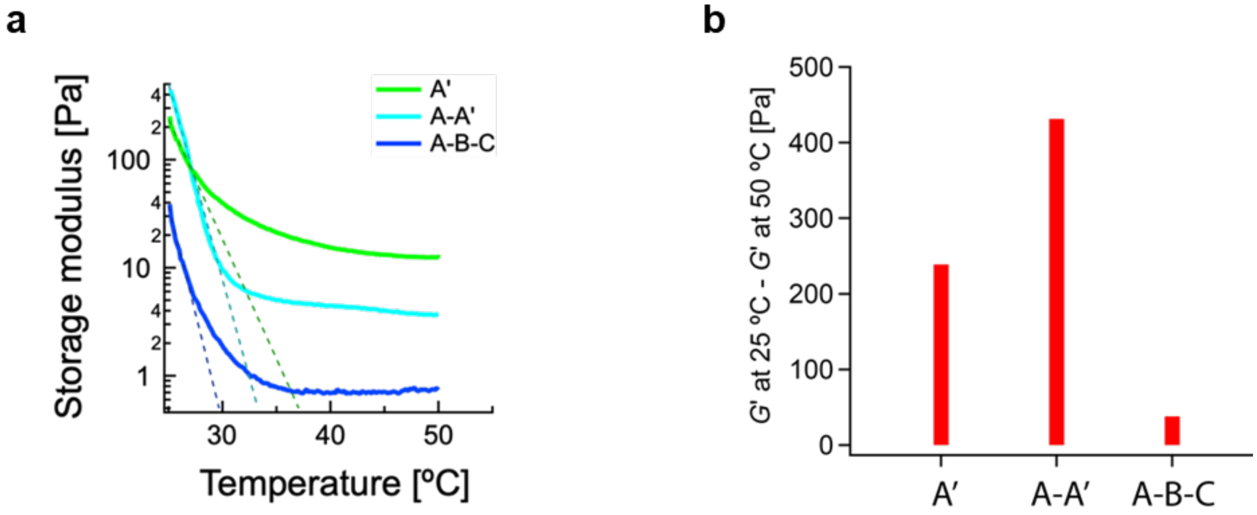

Supplementary Figure 28. *Analysis of mechanical properties of peptide-DNA materials during heating from 25 to 50°C.* (a) Fitting analysis of elasticity decay curves during heating from 25°C to 50°C. Exponential fitting on storage modulus (*G*') phenomenologically reproduced initial decays of the heating process, where T and T_0 are temperatures during the heating cycle and at the starting point of cooling, respectively. The parameter "*a*" represents the dissociation rate of weak crosslinks (A': 0.51 [K^{-1}], A-A': 0.84 [K^{-1}], A-B-C: 0.92 [K^{-1}]). (b) Difference of the storage moduli of peptide-DNA materials during heating from 25°C to 50°C.

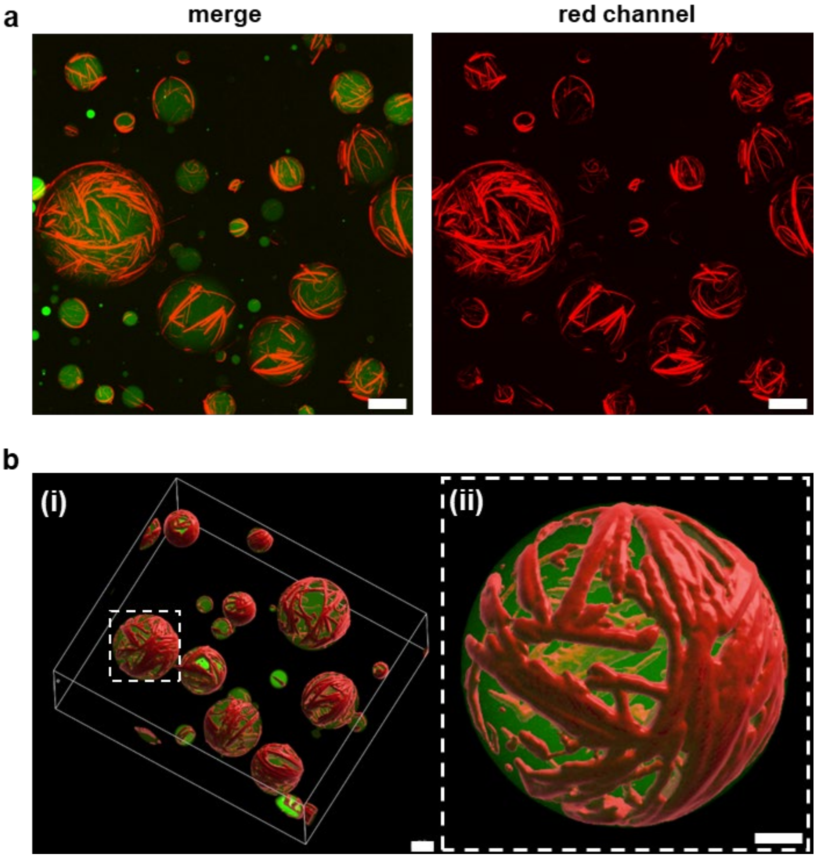

Supplementary Figure 29. *Wide-field confocal images of water-in-oil droplets containing FF cortex and free FITC dye.* (a) Maximum intensity projection confocal images of FF stained with Nile Red (red channel) and 10 μM of free FITC (green channel) within water-in-oil droplets. Left: merged green and red channel image; right: view of only the red channel. Scale bars: 20 μm. (b) 3D views of droplets with the red channel shown as a surface representation to visualize the synthetic cortex. Scale bars: (i) 10 μm, (ii) 5 μm.

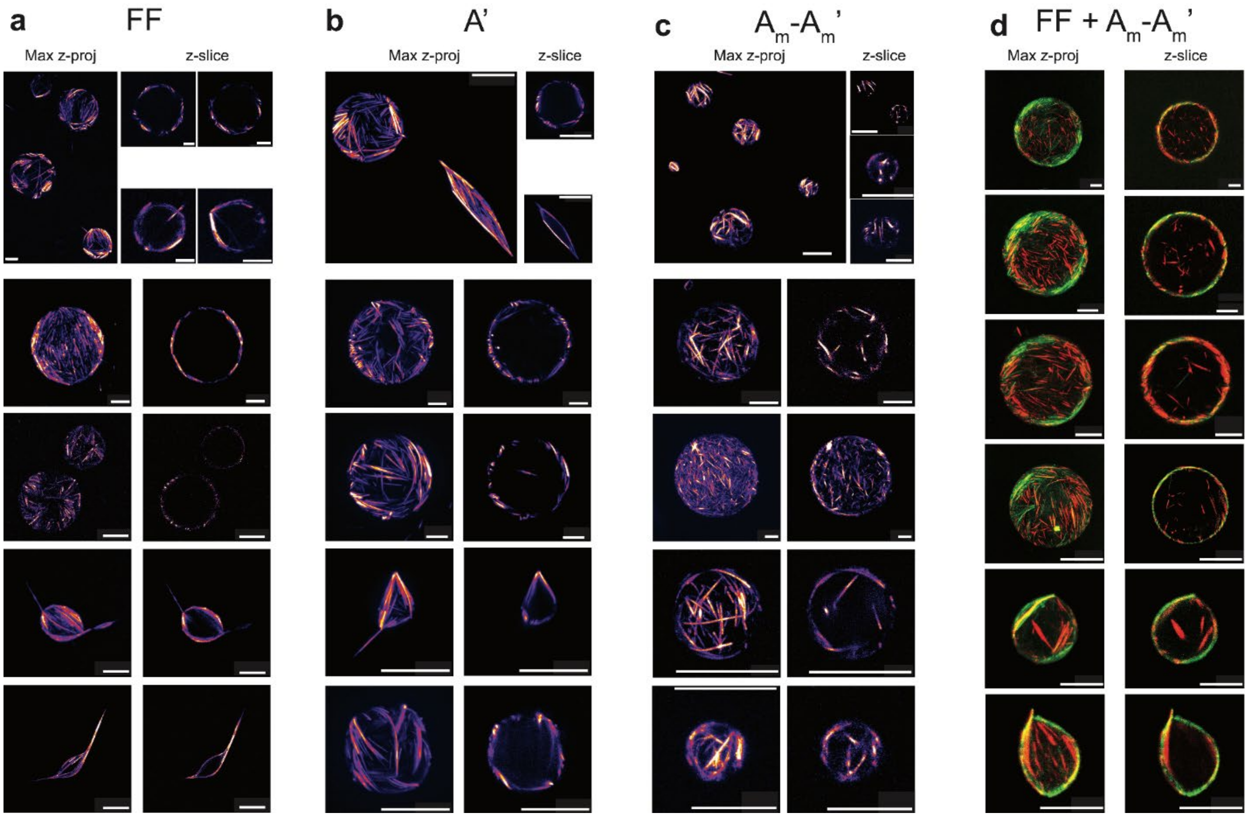

Supplementary Figure 30. *Confocal images of representative water-in-oil droplets including peptide-DNA.* (a) FF (b) A’ (c) A_m-A'_m (d) FF+A_m-A'_m. The left side of each panel displays the maximum intensity projections of z-stacked images, showing the view of the whole droplet. The right side of each panel presents a z-slice through the middle of the droplet, showing the equatorial views of the droplets. Scale bars: 20 µm.

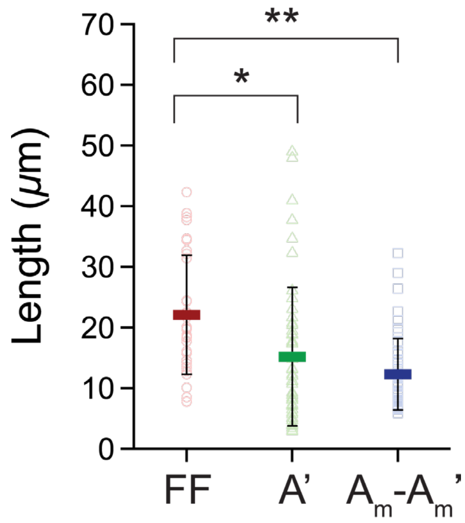

Supplementary Figure 31. *Length distributions of peptide-DNA materials within droplets.* Length of structures within droplets from maximum intensity projection of confocal images are plotted (*n*: 37 filamentous structures for FF, 51 for A', 51 for Am-A'm). Bars represent mean, error bars represent ± SD. Statistics were performed using a two-sided t-test ($p^* < 4.4 \times 10^{-3}$, $p^{**} < 1.4 \times 10^{-7}$).

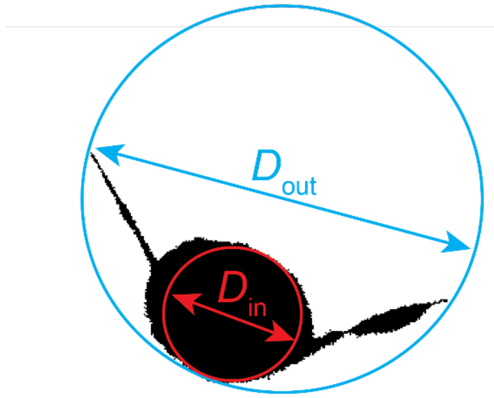

Supplementary Figure 32. *Scheme of shape deformation analysis, D_{in}/D_{out}.* A thresholded image of a droplet from confocal (black), overlayed with the maximum circle size that fits inside the thresholded area (red) and the circle that traces around the outer edge of the area (blue). This measure is used to analyze "sphericity" of droplets.

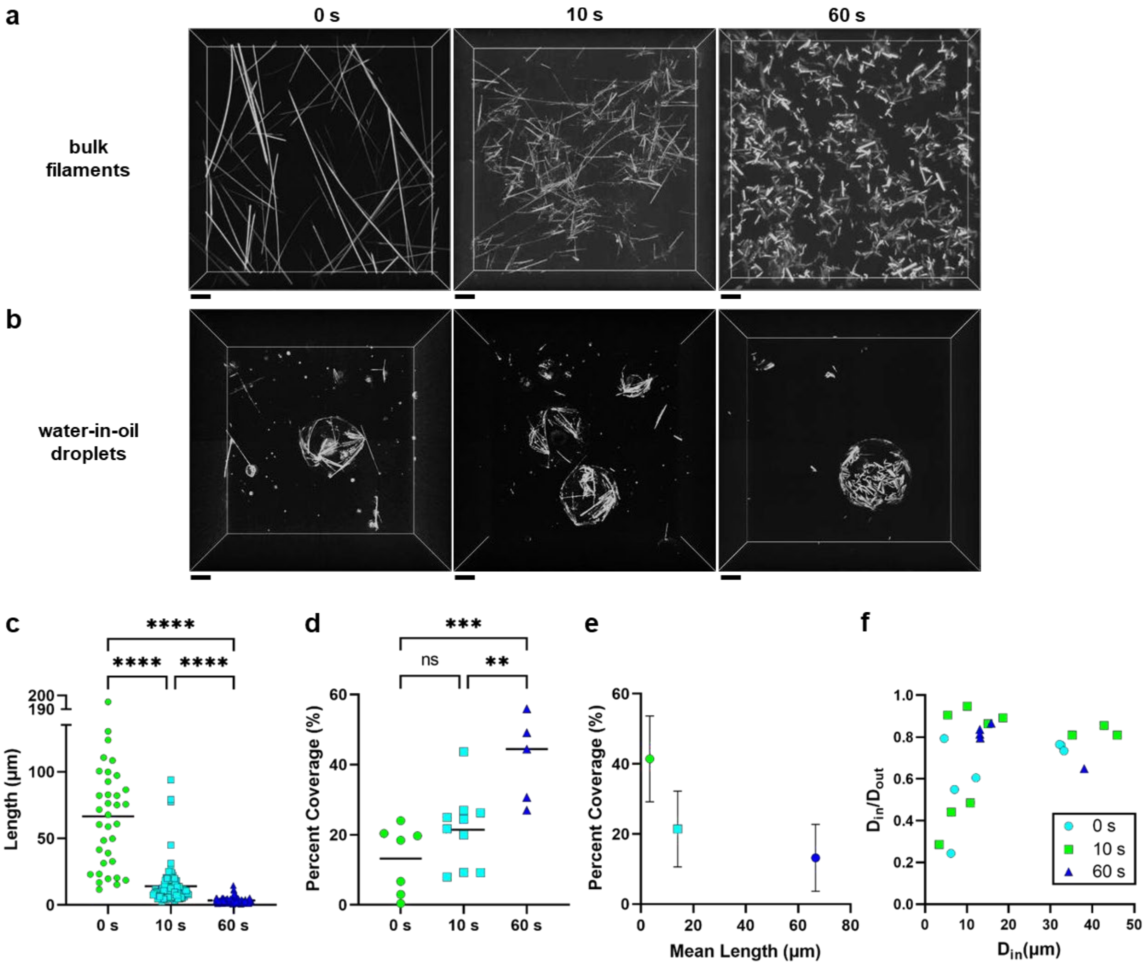

Supplementary Figure 33. *Varying length of peptide filaments within droplets via sonication.* Maximum intensity z-projection confocal images of FF (DMSO/water) assembled with ThT and sonicated to varying lengths (0 s, 10 s, 60 s from left to right, respectively) (a) in the bulk and (b) in water-in-oil droplets. Scale bars: 10 µm. (c) FF filament length quantified from the bulk shown in (a) (*n*: 35 filaments for 0 s, 100 for 10 s, 98 for 60 s). Mean values (black lines): 67 µm for 0 s, 14 µm for 10 s, 3.4 µm for 60 s. Statistics were performed using a Kruskal-Wallis test with a Dunn's multiple comparisons test (****$p = 2.702 \times 10^{-6}$ for 0 s, ****$p < 1 \times 10^{-15}$ for 10 and 60 s). (d-f) Analysis of droplets were performed by thresholding maximum intensity z-projections in two ways: for filaments within droplets and for droplet area (*n*: 7 droplets for 0 s, 10 for 10 s, 5 for 60 s). (d) Percent area of droplets covered by filaments for droplets prepared with varied filament sonication. Mean values (black lines): 13% for 0 s, 21% for 10 s, 41% for 60 s. (e) Percent area (mean and standard deviation) of droplets (*n*: 7 droplets for 0 s, 10 for 10 s, 5 for 60 s) covered by filaments plotted against the mean filament length from (c) (*n*: 35 filaments for 0 s, 100 for 10 s, 98 for 60 s). (f) Sphericity (D_{in}/D_{out}) plotted against size distribution (diameter, D_{in}) of droplets prepared with varied filament sonication, showing that longer filaments deform larger droplets. Statistics were performed using an ordinary one-way ANOVA test with Tukey's multiple comparisons test (*** $p = 0.0007$, ** $p \leq 0.0082$, ns = 0.2926).

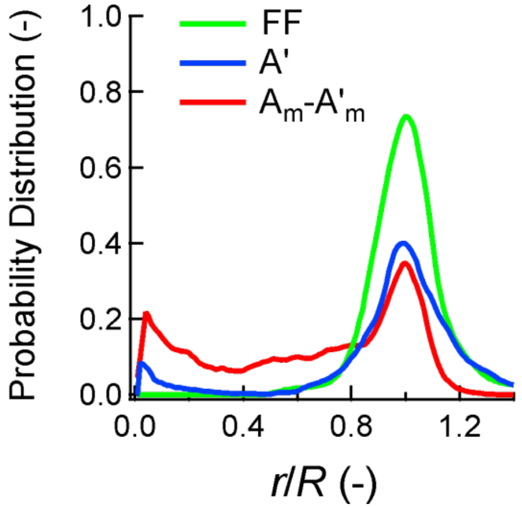

Supplementary Figure 34. The probability distribution of structures across the diameter of droplets smaller than ~40 µm. (n = 10 for FF, n = 12 for A', n = 14 for A_m-A'_m). Due to the non- spherical shapes of smaller droplets, we define R as the radius of the maximum inscribed circle within the cross-section of the droplets. (The probability distribution of structures across the diameter of droplets larger than ~40 µm is shown in main **Fig. 4h(ii)**).

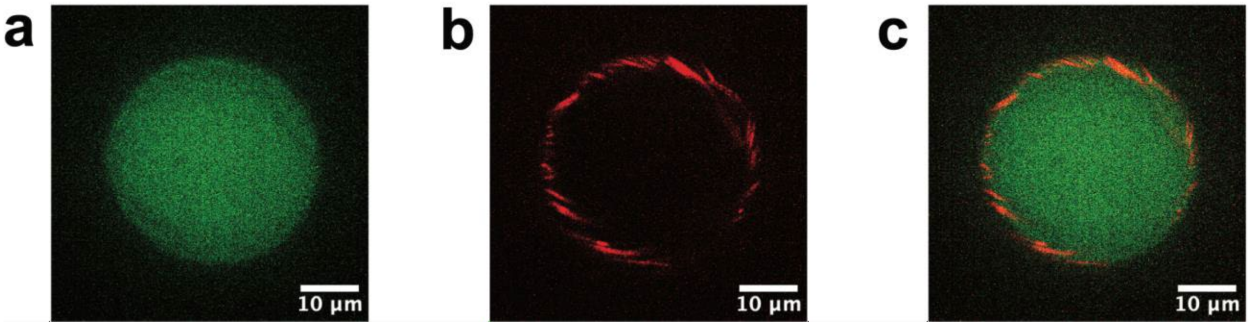

Supplementary Figure 35. *Water-in-oil droplets containing FF and 20 µM A-FITC.* (a) Green channel: FITC, (b) Red channel: Nile Red, (c) merged channels.

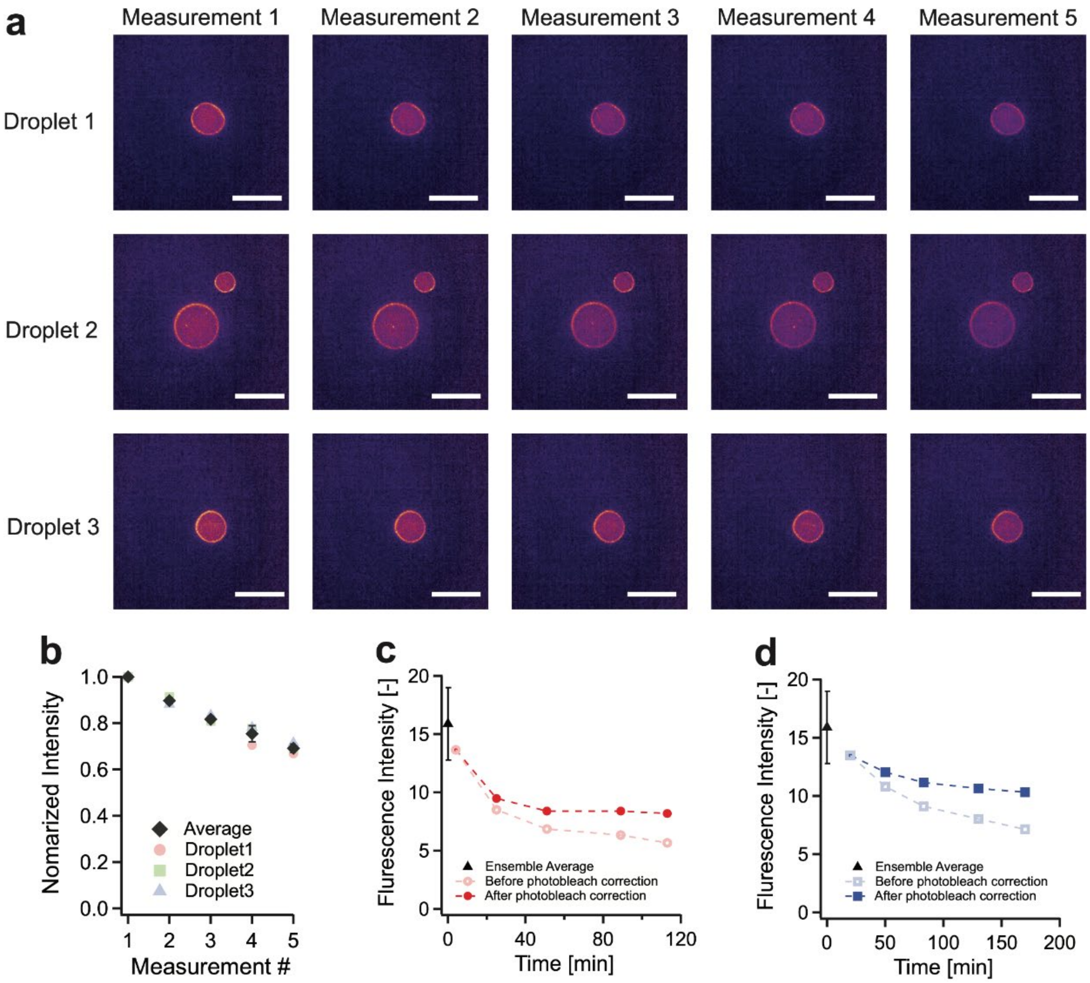

Supplementary Figure 36. *Photobleaching correction for DNA-triggered release of payloads from synthetic cortexes*. (a) The photobleaching process of FITC was monitored in z-sections of 3D *z*-stacks at equator planes of droplets containing A' fibers conjugated with A-FITC. Imaging was done under the same laser intensity and *z* spacing used for the release experiments shown in **Extended Fig. 3**. Green fluorescence images are falsely-colored for visualization purposes. Scale bars: 20 µm. (b) FITC fluorescence intensities along the contour of the shell, normalized by dividing the fluorescence intensity of each subsequent image by that of the first image. (c,d) The time course of fluorescence intensities during the release process was measured using (c) 100 nM and (d) 400 nM A-invader (A-I) strand, before and after correcting for the photobleaching effect. n=8 droplets were analyzed for 0 uM A-I, used as an initial time point (t=0). Error bars represent ± SD.

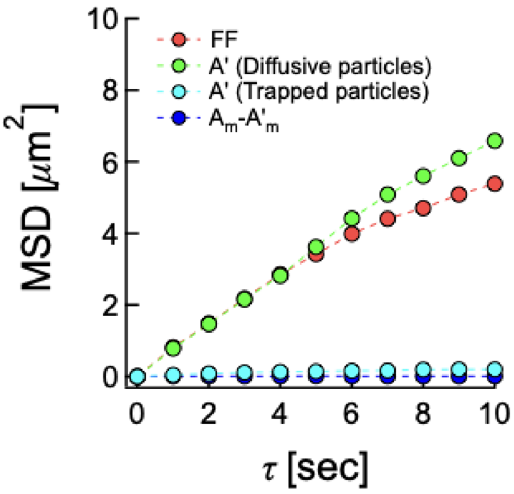

Supplementary Figure 37. *MSDs of probe particles inside droplets with FF, A', and A_m-A'_m on a linear time scale*. Data from Fig. 5c re-plotted on a linear time scale.

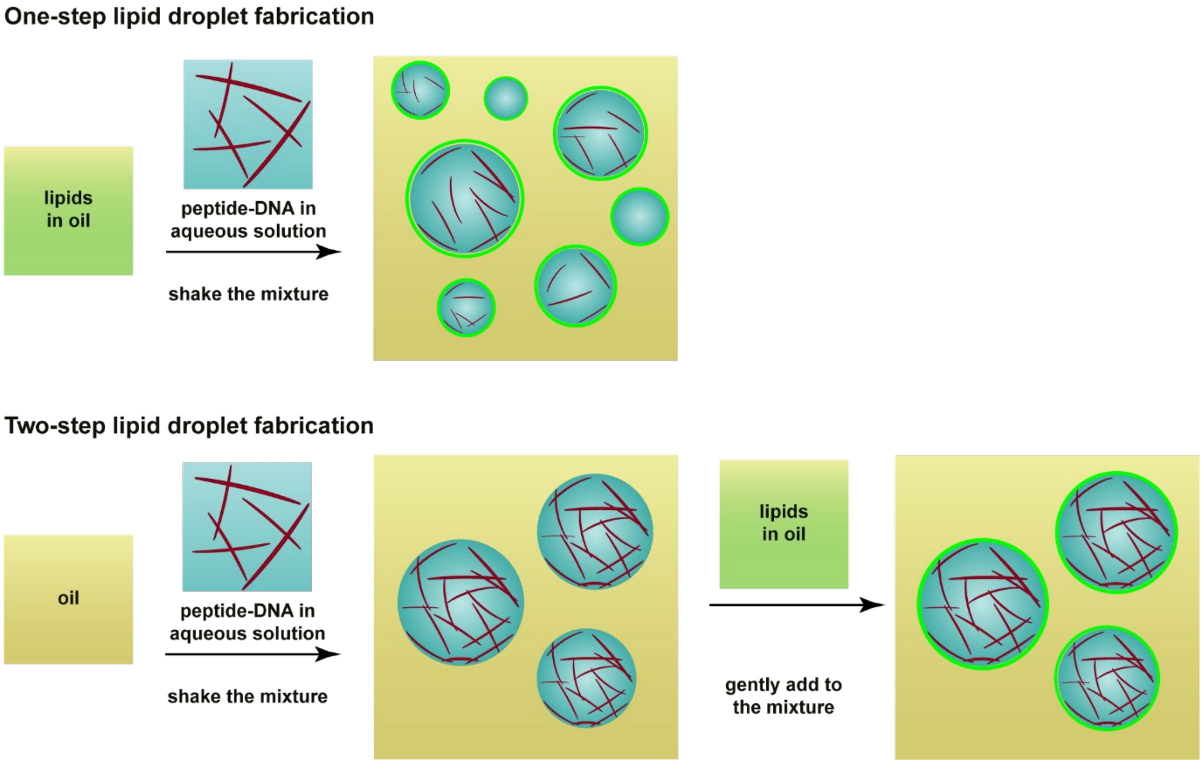

Supplementary Figure 38. *Illustration of the lipid droplet fabrication methods.*

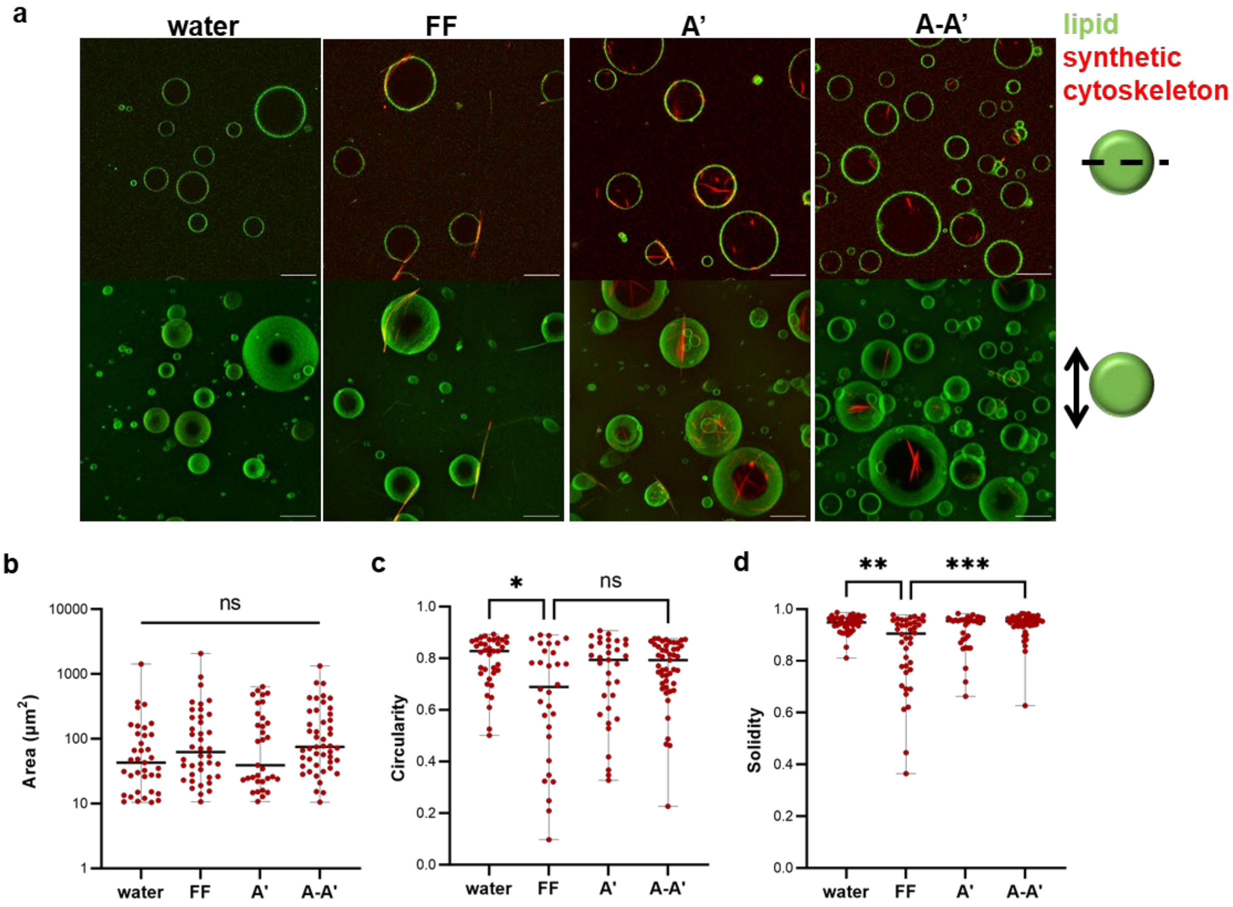

Supplementary Figure 39. *Area, circularity and solidity of one-step lipid-oil droplets.* (a) Wide-field confocal images of one-step lipid-oil droplets containing water, FF, A' and A-A' (from left to right), shown as one z-slice (top) and a maximum intensity projection of a z-stack (bottom). Scale bars: 20 µm. Analysis of (b) area, (c) circularity (4π(area÷perimeter^2)) and (d) solidity (area÷convex hull) from maximum intensity projections of z-stack confocal images of droplets containing water, FF, A' and A-A' encased in lipid-oil droplets (number of droplets analyzed: 37 for water, 30 for FF, 33 for A', 47 for A-A'). (b-d) Horizontal black line: median, vertical bars: range. Statistics performed using Kruskal-Wallis t-tests and Dunn's multiple comparisons test ((b): non-significant $p = 0.129$ for water vs. FF, 0.071 for water vs. A-A', $p > 1.000$ for water vs. A', FF vs. A', FF vs. A-A', A' vs. A-A'; (c): * $p = 0.0284$ for water vs. FF, non-significant $p = 0.9074$ for water vs. A', $p = 0.9886$ for FF vs. A', $p = 0.5574$ FF vs. A-A', $p > 0.9999$ for water vs. A-A' and A- vs. A-A'; (d): ** $p = 0.0027$ for water vs. FF, *** $p = 0.0008$ for FF vs. A-A', non-significant $p = 0.1131$ for FF vs. A', $p > 0.9999$ for water vs. A', water vs. A-A', A' vs. A-A'). Circularity and solidity measurements show increased deformations in FF droplets.

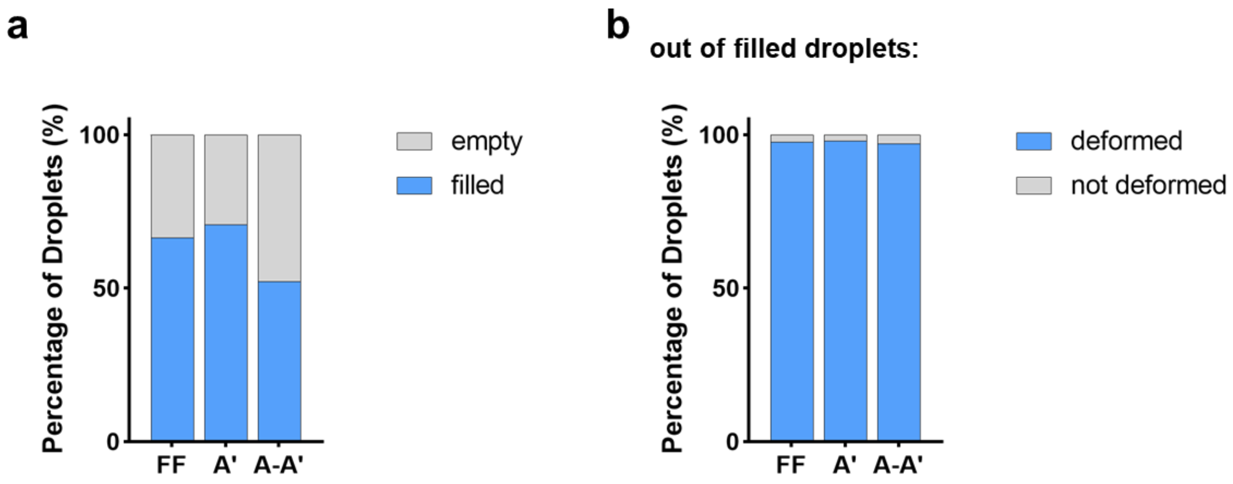

Supplementary Figure 40. *Percentage of one-step lipid droplets encapsulating structures or presenting deformations*. (a) Percentage of lipid droplets containing ("filled") or not containing ("empty") structures. Number of droplets analyzed: *n* = 125 for FF, *n* = 72 for A', *n* = 134 for A-A'. (b) Percentage of filled lipid-droplets exhibiting deformations (showing qualitatively a protrusion, a flat edge, or both), out of the number of filled droplets in each sample. Number of filled droplets analyzed: *n* = 83 for FF, *n* = 51 for A', *n* = 70 for A-A'.

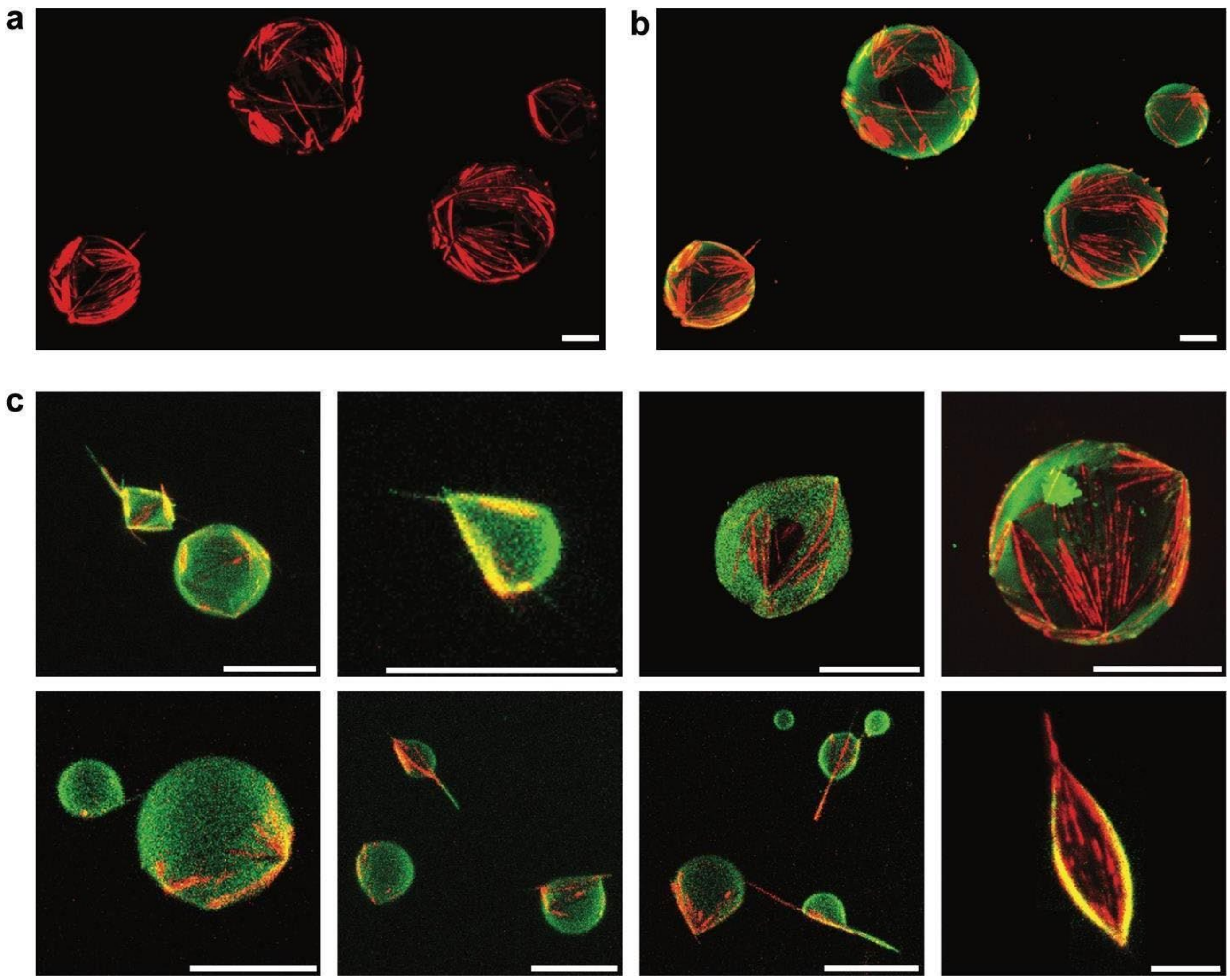

Supplementary Figure 41. *Lipid-coated two-step FF droplets.* (a,b) Maximum z-projections of FF shells (a) before and (b) after coating with DOPE. (Green: Atto 488 DOPE, Red: FF stained with Nile Red) (c) Representative maximum z-projections of non-spherical droplets coated with DOPE/Atto488-DOPE are shown. Generally, FF lipid droplets smaller than ~30 μm tend to exhibit non-spherical shapes. Scale bars: 20 μm.

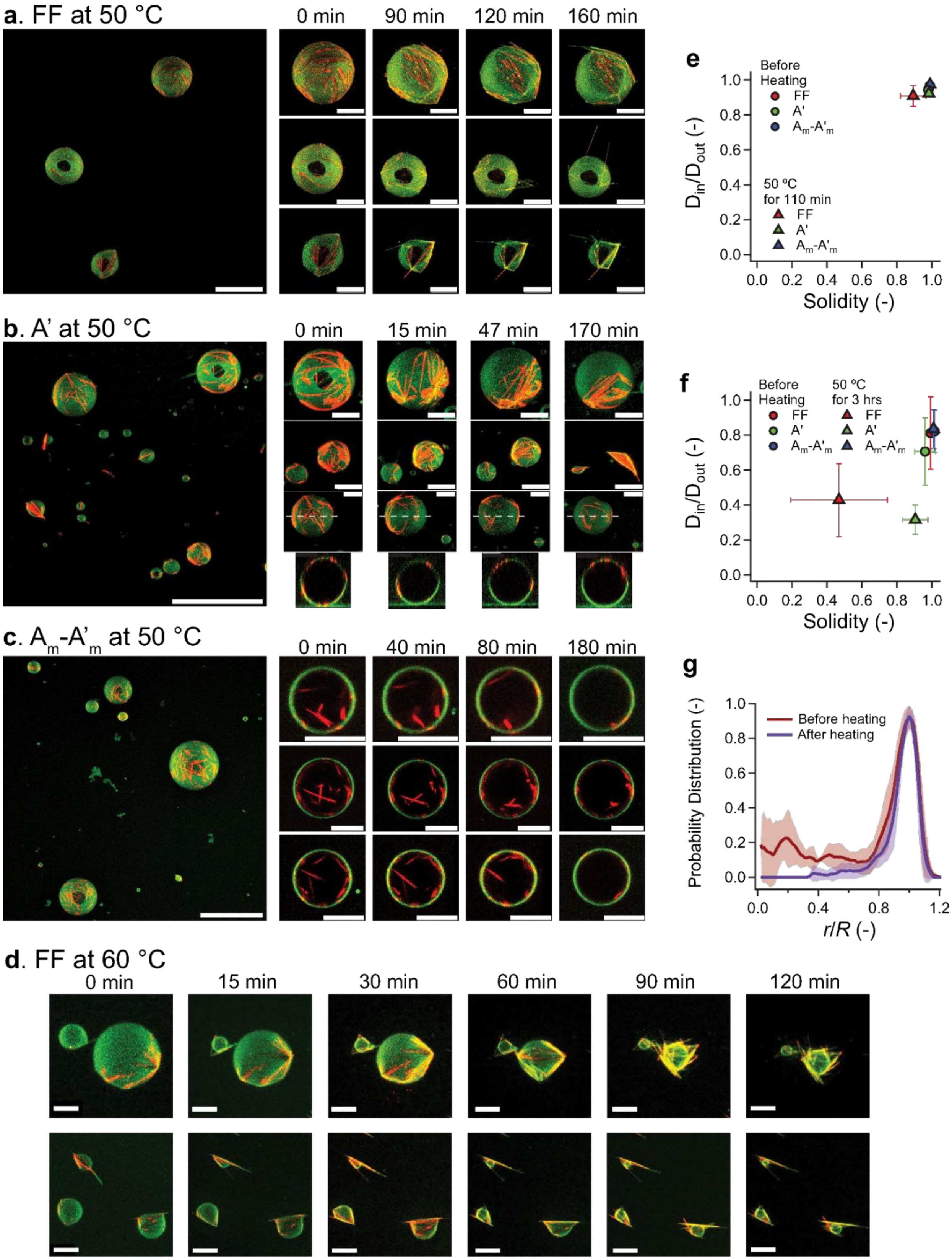

a. FF at 50 °C
0 min
90 min
120 min
160 min
b. A' at 50 °C
0 min
15 min
47 min
170 min
c. A_m-A'_m at 50 °C
0 min
40 min
80 min
180 min
d. FF at 60 °C
0 min
15 min
30 min
60 min
90 min
120 min
e
Before Heating
FF
A'
A_m-A'_m
50 °C for 110 min
FF
A'
A_m-A'_m
D_{in}/D_{out} (-)
Solidity (-)
f
Before Heating
FF
A'
A_m-A'_m
50 °C for 3 hrs
FF
A'
A_m-A'_m
D_{in}/D_{out} (-)
Solidity (-)
g
Before heating
After heating
Probability Distribution (-)
r/R (-)

Supplementary Figure 42. *Confocal microscopy of lipid-coated droplets upon heating* (Red: NileRed, Green: DOPE/Atto488-DOPE) (a) Heating process of FF droplets at 50 °C. Representative maximum z-projections of (a1) a large field of view and (a2) time series of selected individual droplets from (a1). The entire time series is shown in **Supplementary Video 13**. Scale bars: 50 μm and 20 μm for (a1) and (a2), respectively. (b) Heating process of A' droplets at 50 °C. Representative maximum z-projections of (b1) a large field of view and (b2) time series of zoomed-in views. Scale bars: 50 μm and 20 μm for (b1) and (b2), respectively. The entire time series is shown in **Supplementary Video 14**. To show the clustering of A' fibers on the shell of the droplet, we took slices perpendicular to the xy plane, shown with white dotted lines. The corresponding sliced images are shown in the bottom row of (b2). Scale bars: 50 μm and 10 μm for (b1) and (b2), respectively. (c) Heating process of A_m- A'_m droplets at 50 °C. Representative maximum z-projections of (c1) a large field of view (c1) and (c2) time series of cross-sections of selected droplets from (c1). Scale bars: 50 μm and 20 μm for (c1) and (c2), respectively. The entire time series is shown in **Supplementary Video 15**. (d) Maximum z-projections from confocal microscopy of FF deformed droplets < 20 μm upon heating to 60°C over time. Each row corresponds to a different field of view. (e) Global shape changes of large spherical droplets > 25 μm before and after 110 min of heating at 50°C. The mean of the circularity (D_{in}/D_{out}) was plotted against the solidity. (f) Global shape changes of large spherical droplets > 25 μm before and after 3 hours of heating at 50°C. The mean of D_{in}/D_{out} was plotted against the solidity. (g) The effect of heat on the radial spatial distribution of peptide-DNA structures within lipid-droplets. The probability distribution of structures across the diameter of A_m-A'_m droplets before and after heating to 50 °C for 3 hours are plotted as red and purple solid lines, respectively. The error bars in panels e, f, and g represent ± SD from the heating process of 13 droplets for FF, 8 droplets for A', and 10 droplets for A_m-A'_m.

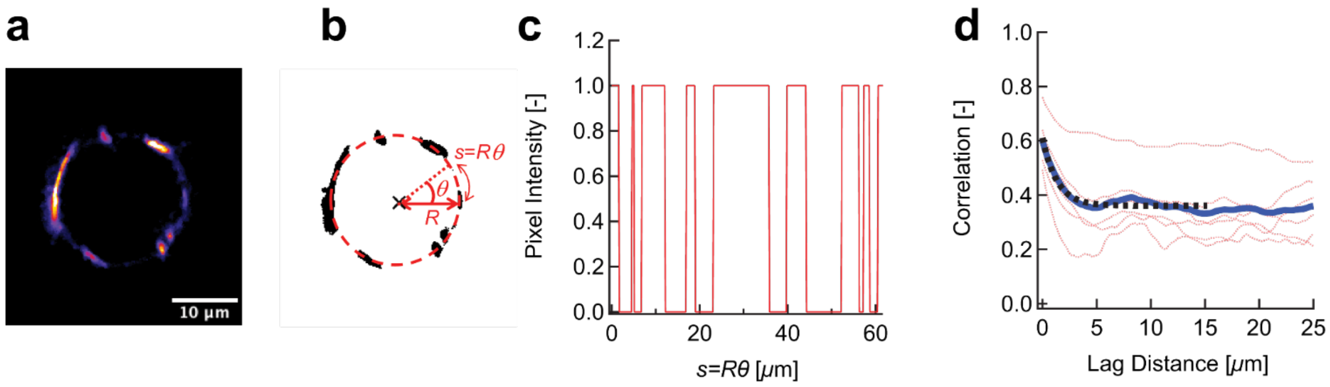

Supplementary Figure 43. *Correlation analysis along the contour of droplets.* (a) A single z-slice of A' droplet without lipids. The image is falsely-colored for visualization purposes. (b) Thresholded image of (a). The coordinate *s*=*Rθ* is defined as the contour length along the oil-water interface, where *R* and *θ* represent the radius of the oil-water interface and the angle, respectively. (c) Pixel intensities of the binarized image along the oil-water interface. (d) The spatial correlation of pixel intensities along the contour. Individual and averaged correlation curves are shown as red dotted lines and a blue solid line, respectively. A black dotted line denotes a fitting result with an exponential function.

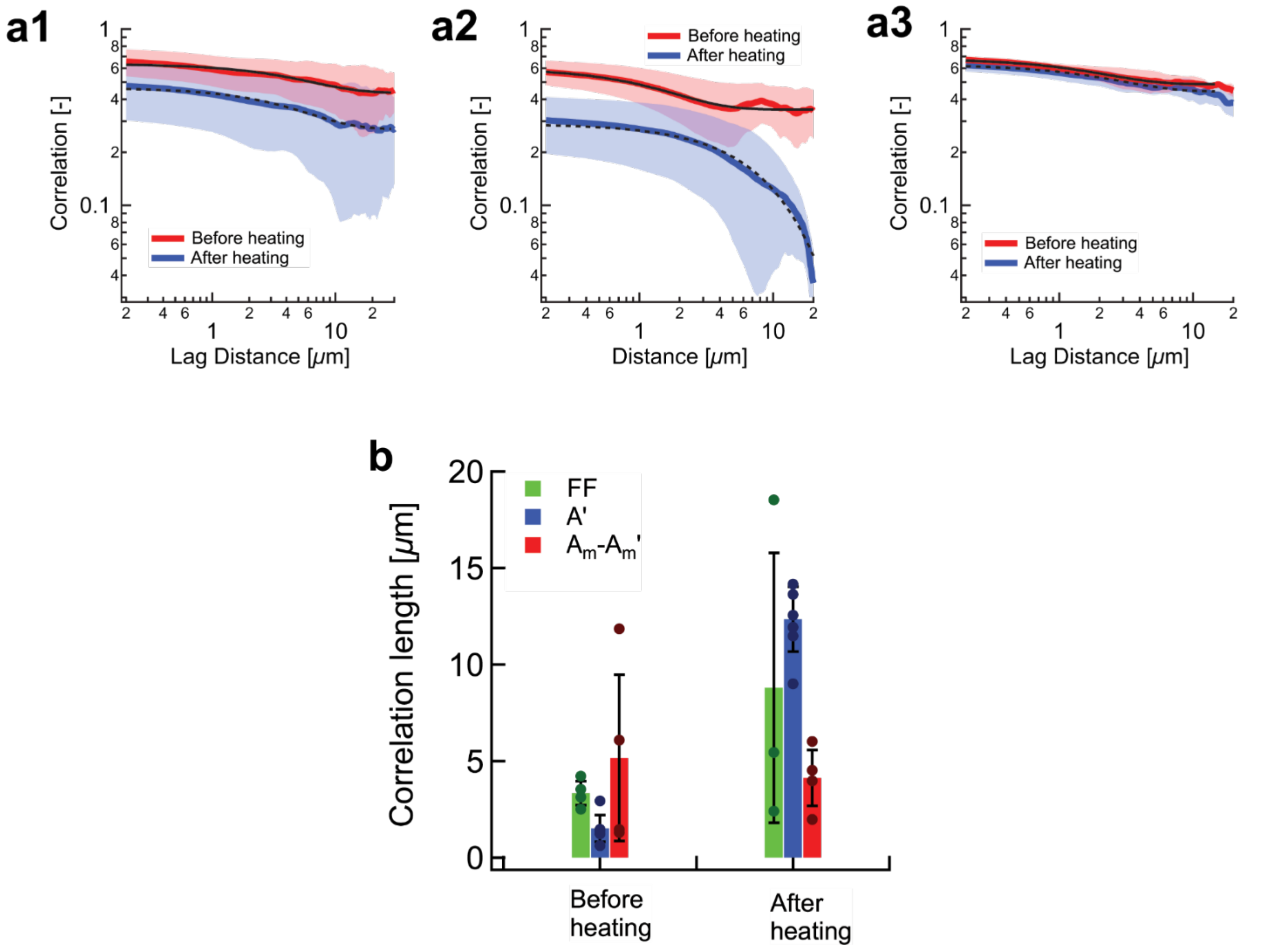

Supplementary Figure 44. *The effect of heat on filament distributions at the oil-water boundaries of large spherical lipid droplets with peptide-DNA.* (a) Spatial correlation of fibers along the contour of oil-water boundaries for (a1) FF, (a2) A', and (a3) A_m-A'_m lipid droplets before and after heating. Black lines are fitting results with an exponential function. (b) Comparing the effect of heat on correlation lengths across the different droplets. Individual values and the mean of correlation lengths of fibers along the contour of oil-water boundaries are represented by markers and bar plots, respectively. Error bands/bars both in (a) and (b) represent ± SD of n=4, 6, and 4 correlation functions for FF, A', and A_m-A'_m, respectively.

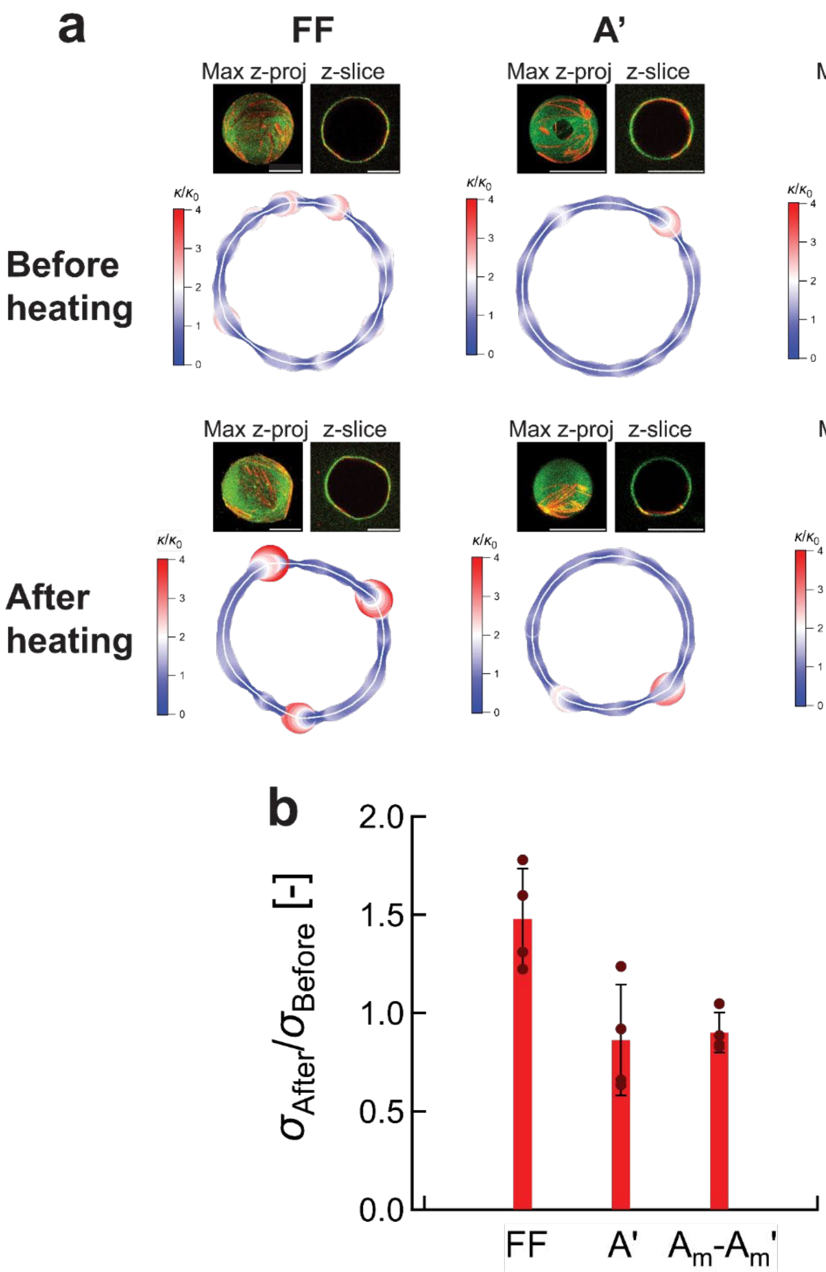

Supplementary Figure 45. *Local curvature analysis of peptide-DNA lipid droplets (two-step fabrication) before and after 90 min heating at 50°C.* (a) We traced the contour of droplets at *z*-slices with the JFilament plugin in ImageJ.[12] Their local curvatures, denoted as 'κ', were evaluated using the referencePathFrenet object in MATLAB. Additionally, we determined their global curvatures by fitting the contours with a circle, defined as 'κ_0'. Along the contour (represented by white solid lines), we show the normalized curvatures (κ/κ_0) before and after heating. The sizes of the circles correspond to the absolute values of κ/κ_0. Contour maps and confocal microscopy images of droplets before (top) and after (bottom) heating are shown (the "after heating" contour maps are also shown in **Fig. 6**. (b) We evaluated the ratio of the standard deviations of κ/κ_0 values before and after heating, which we denoted as 'σ_{before}' and 'σ_{after}', respectively. Individual values of $\sigma_{after}/\sigma_{before}$ are represented by markers. Bar plots represent the mean of $\sigma_{after}/\sigma_{before}$, with error bars indicating ± SD of 4 cross-sections.

Supplementary Video Captions

Supplementary Video 1. *Confocal microscopy time lapse of peptide fibers for bending stiffness calculations*.

Supplementary Video 2. *Confocal microscopy time lapse of peptide-DNA at elevated temperatures*. A' was heated up to 60 °C, then imaged on a room-temperature stage. The video was taken up to 10 min after the sample started cooling down. The duration of each movie was 30 sec.

Supplementary Video 3. *Confocal microscopy of FF droplet through z slices*. Confocal microscopy images of a droplet containing FF, shown as a progression throughout the z-planes of the droplet.

Supplementary Video 4. *Confocal microscopy of A' droplet through z slices*. Confocal microscopy images of a droplet containing A', shown as a progression throughout the z-planes of the droplet.

Supplementary Video 5. *Confocal microscopy of A_m-A'_m droplet through z slices*. Confocal microscopy images of a droplet containing A_m-A'_m, shown as a progression throughout the z-planes of the droplet.

Supplementary Video 6. *Confocal microscopy of FF/A_m-A'_m droplet through z slices*. Confocal microscopy images of a droplet containing FF and A_m-A'_m, shown as a progression throughout the z- planes of the droplet.

Supplementary Video 7. *Confocal microscopy of sonicated FF in droplets through z slices.* Confocal microscopy images of a droplet containing FF sonicated for 0, 10 or 60 s before encapsulation, shown as a progression throughout the z-planes of the droplet.

Supplementary Video 8. *Particles diffusing freely in bulk FF networks.* Confocal microscopy time lapse of beads (1 µm, green microspheres, carboxylic acid functionalized) mixed with peptide filaments. The duration of the movie was 100 sec.

Supplementary Video 9. *Particle dynamics within an FF droplet.* Confocal microscopy time lapse of beads (1 µm, green microspheres, carboxylic acid functionalized) in FF droplets. The duration of the movie was 100 sec.

Supplementary Video 10. *Particle dynamics within an A' droplet.* Confocal microscopy time lapse of beads (1 µm, green microspheres, carboxylic acid functionalized) in A' droplets. The duration of the movie was 100 sec.

Supplementary Video 11. *Particle dynamics within an A_m-A'_m droplet.* Confocal microscopy time lapse of beads (1 µm, green microspheres, carboxylic acid functionalized) in A_m-A'_m droplets. The duration of the movie was 100 sec.

Supplementary Video 12. *Filament dynamics in heated peptide and peptide-DNA droplets*. Confocal microscopy time lapses of droplets containing FF, A' and A_m-A'_m during heating to 50°C over time. The duration of the movies for FF, A', and A_m-A'_m droplets was 160, 180, and 180 min, respectively.

Supplementary Video 13. *Heating droplets containing FF*. Confocal microscopy time lapses of droplets containing FF during heating to 50°C over time, with insets showing regions of interest. The duration of the movie was 160 min.

Supplementary Video 14. *Heating droplets containing A'*. Confocal microscopy time lapses of droplets containing A' during heating to 50°C over time, with insets showing regions of interest. The duration of the movie was 180 min.

Supplementary Video 15. *Heating droplets containing A_m-A'_m*. Confocal microscopy time lapses of droplets containing A_m-A'_m during heating to 50°C over time, with insets showing regions of interest. The duration of the movie was 180 min.

REFERENCES

1. Gittes, F., Mickey, B., Nettleton, J. & Howard, J. Flexural rigidity of microtubules and actin filaments measured from thermal fluctuations in shape. *J. Cell Biol.* **120**, 923–934 (1993).

2. Fakhri, N., Tsyboulski, D. A., Cognet, L., Weisman, R. B. & Pasquali, M. Diameter-dependent bending dynamics of single-walled carbon nanotubes in liquids. *Proc. Natl. Acad. Sci.* **106**, 14219–14223 (2009).
3. Brangwynne, C. P. *et al.* Bending Dynamics of Fluctuating Biopolymers Probed by Automated High-Resolution Filament Tracking. *Biophys. J.* **93**, 346–359 (2007).
4. Li, Y. *et al.* Controlled assembly of dendrimer-like DNA. *Nat. Mater.* **3**, 38–42 (2004).
5. Zadeh, J. N. *et al.* NUPACK: Analysis and design of nucleic acid systems. *J. Comput. Chem.* **32**, 170–173 (2011).
6. Bendix, P. M. *et al.* A Quantitative Analysis of Contractility in Active Cytoskeletal Protein Networks. *Biophys. J.* **94**, 3126–3136 (2008).
7. Weirich, K. L. *et al.* Liquid behavior of cross-linked actin bundles. *Proc. Natl. Acad. Sci.* **114**, 2131–2136 (2017).
8. Gardel, M. L. *et al.* Elastic Behavior of Cross-Linked and Bundled Actin Networks. *Science* **304**, 1301–1305 (2004).
9. Shin, J. H., Gardel, M. L., Mahadevan, L., Matsudaira, P. & Weitz, D. A. Relating microstructure to rheology of a bundled and cross-linked F-actin network *in vitro*. *Proc. Natl. Acad. Sci.* **101**, 9636–9641 (2004).
10. Francis, M. L. *et al.* Non-monotonic dependence of stiffness on actin crosslinking in cytoskeleton composites. *Soft Matter* **15**, 9056–9065 (2019).
11. Fitzpatrick, R. *et al.* Synergistic Interactions Between DNA and Actin Trigger Emergent Viscoelastic Behavior. *Phys. Rev. Lett.* **121**, 257801 (2018).
12. Smith, M. B. *et al.* Segmentation and tracking of cytoskeletal filaments using open active contours. *Cytoskeleton* **67**, 693–705 (2010).
